# Comparative genomic analysis of the ‘pseudofungus’ *Hyphochytrium catenoides*

**DOI:** 10.1098/rsob.170184

**Published:** 2018-01-10

**Authors:** Guy Leonard, Aurélie Labarre, David S. Milner, Adam Monier, Darren Soanes, Jeremy G. Wideman, Finlay Maguire, Sam Stevens, Divya Sain, Xavier Grau-Bové, Arnau Sebé-Pedrós, Jason E. Stajich, Konrad Paszkiewicz, Matthew W. Brown, Neil Hall, Bill Wickstead, Thomas A. Richards

**Affiliations:** 1 Living Systems Institute, Department of Biosciences, University of Exeter, Exeter EX4 4QD, UK; 2 Department of Plant Pathology and Microbiology, Institute for Integrative Genome Biology, University of California, Riverside, CA 92506, USA; 3 Institute of Evolutionary Biology, CSIC-UPF, Barcelona, Catalonia, Spain; 4 Weizman Institute of Science, Rehovot, Israel; 5 Department of Biological Sciences, Mississippi State University, Mississippi State, MS 39762, USA; 6 Institute for Genomics, Biocomputing and Biotechnology, Mississippi State University, Mississippi State, MS 39762, USA; 7 School of Biological Sciences, University of East Anglia, Norwich Research Park, Norwich NR4 7TJ, UK; 8 School of Life Sciences, University of Nottingham, Nottingham NG7 2UH, UK

**Keywords:** polarized filamentous growth, large DNA virus, oomycete parasitic traits, secondary plastid endosymbiosis

## Abstract

Eukaryotic microbes have three primary mechanisms for obtaining nutrients and energy: phagotrophy, photosynthesis and osmotrophy. Traits associated with the latter two functions arose independently multiple times in the eukaryotes. The Fungi successfully coupled osmotrophy with filamentous growth, and similar traits are also manifested in the Pseudofungi (oomycetes and hyphochytriomycetes). Both the Fungi and the Pseudofungi encompass a diversity of plant and animal parasites. Genome-sequencing efforts have focused on host-associated microbes (mutualistic symbionts or parasites), providing limited comparisons with free-living relatives. Here we report the first draft genome sequence of a hyphochytriomycete ‘pseudofungus’; *Hyphochytrium catenoides*. Using phylogenomic approaches, we identify genes of recent viral ancestry, with related viral derived genes also present on the genomes of oomycetes, suggesting a complex history of viral coevolution and integration across the Pseudofungi. *H. catenoides* has a complex life cycle involving diverse filamentous structures and a flagellated zoospore with a single anterior tinselate flagellum. We use genome comparisons, drug sensitivity analysis and high-throughput culture arrays to investigate the ancestry of oomycete/pseudofungal characteristics, demonstrating that many of the genetic features associated with parasitic traits evolved specifically within the oomycete radiation. Comparative genomics also identified differences in the repertoire of genes associated with filamentous growth between the Fungi and the Pseudofungi, including differences in vesicle trafficking systems, cell-wall synthesis pathways and motor protein repertoire, demonstrating that unique cellular systems underpinned the convergent evolution of filamentous osmotrophic growth in these two eukaryotic groups.

## 1. Introduction

Stramenopiles [1] (also known as heterokonts [2]) are a highly diverse branch of protists that encompass a multitude of biological forms including: huge multicellular kelps (seaweeds), abundant marine micro-algae and a variety of microbial parasites, some of which (e.g. oomycetes) feed and grow like fungi and cause important diseases of animals, algae and plants [3,4]. The stramenopiles are a phylogenetically robust group (e.g. [5]) defined by the presence of two motile flagella, a ‘standard’ smooth posterior flagellum and a ‘tinselate’ anterior flagellum with a tripartite rigid tubular mastigoneme (hairs) [2]. However, secondary flagellum loss has occurred during the radiation of this group, for example in the hyphochytrids like *Hyphochytrium catenoides* [6], which have lost a smooth posterior flagellum but retained a tinselate anterior flagellum.

Environmental sequencing, specifically of marine environments (e.g. [7]), has increased the known phylogenetic diversity of the stramenopiles, suggesting that this group is one of the most diverse higher-level groups within the eukaryotes [8]. Representatives of these groups remain uncultured with little gene/genome sampling. Furthermore, genome-sequencing efforts in the stramenopiles have largely focused on photosynthetic algae (e.g. [9,10]) or oomycete parasites (e.g. [11,12]), leaving the diversity of heterotrophic free-living stramenopiles undersampled. Here, we describe the sequencing and comparative genomic analysis of *H. catenoides* (ATCC 18719) originally isolated by D. J. Barr from pine tree pollen in Arizona, USA (however, we note that there is no direct reference in ATCC that accompanies this culture [13]). We propose this organism and associated genome data as a tool to investigate the evolution of stramenopile characteristics and for the purpose of comparing and contrasting the evolution of traits between free-living and parasitic Pseudofungi.

**Figure 1.**
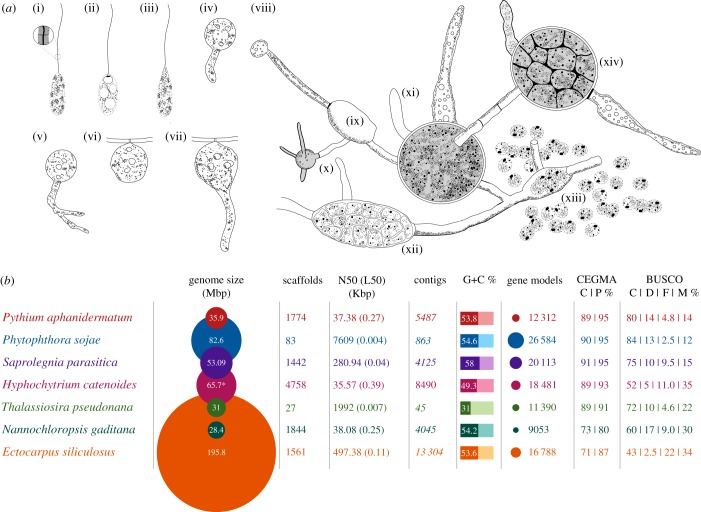
Developmental characteristics of *H. catenoides* and genome statistics of representative stramenopiles. Sketches of a subset of different stages of *H. catenoides* life cycle, adapted and redrawn from [6,14] showing: (i–iii) different views of zoospores (including magnification of tinselate flagellum i), (iv) germination stage of large spore, (v) primary enlargement or primary sporangium, (vi,vii) thallus development on substrate, (viii) unusual extensive branched thallus, which consists of separated sporangia at different stages of maturity (e.g. xii,xiv), connected by long, tubular, septate, hyaline and empty hyphae (x,xi), sometimes with enlargements without sporangia (e.g. ix). Zoospores may fail to swim coming to rest near exit tube (xiii). (*b*) Table of genome statistics for a range of different stramenopiles. Asterisk indicates k-mer estimation of genome size (column 2). All numbers are from the respective genome datasets (see electronic supplementary material, table S12). Numbers in italics (contigs, column 5) are inferred from the scaffolded data. CEGMA: C, complete; P, partial recovered gene models. BUSCO: C, complete; D, duplicated; F, fragmented; M, missing gene models.

*Hyphochytrium catenoides* is a free-living hyphochytrid protist that forms hyphal-like networks and spores with only a single anterior tinselate flagellum (figure 1*a*) [6,14]. The hyphochytrids are thought to branch sister to the oomycetes [4,15], and both these groups grow as filamentous/polarized cells feeding osmotrophically by extracellular secretion of digestive enzymes coupled to nutrient uptake [4,6,14]. These characteristics mean that they ‘resemble’ fungi [4]. Here, we use genome sequence data to confirm the phylogenetic position of the hyphochytrids, investigate characters shared with oomycete parasites and identify the genes involved in cellular characteristics shared with fungi that characterize filamentous/osmotrophic growth. We also use the genome data to investigate the protein repertoire putatively associated with loss of the posterior flagellum in the hyphochytrids. These data provide a unique genome sample of a free-living stramenopile in order to facilitate further evolutionary and cellular research.

## 2. Results and discussion

### 2.1. Genome assembly and gene model prediction

Using a range of methods, we assembled and tested the completeness of the *H. catenoides* genome (see Material and methods). Comparisons measuring the fraction of transcriptome data that aligned to the genome with BLAST, along with CEGMA and BUSCO v.1.2, demonstrated that the genome assembly was predicted to be, respectively, 97.8%, 91.5% and 52% complete in terms of gene sampling (for further analysis and discussion of genome ‘completeness’ analysis, see electronic supplementary material, figure S1). Both CEGMA and BUSCO (v. 1.2) are likely to underestimate the completeness of genomes, as the core gene list is derived from a subset of genomes that does not fully sample a diverse collection of eukaryotic genomes (e.g. BUSCO v. 1.2 only samples fungal and metazoan genomes), which inevitably gives a much lower estimation of completion. A full set of tRNAs was identified in the *Hyphochytrium* genome, including an additional tRNA for selenocysteine. The ≥1 kbp scaffold assembly along with the predicted proteome has been submitted as a draft genome to the EMBL EBI (BioStudies: S-BSST46). Details comparing the assembly with other eukaryotic genome sequences are described in figure 1*b*. Analysis using RepeatMasker [16] determined that the ≥1 kbp genome assembly comprised 9.53% repeat regions of which 1.79% were assigned to transposable elements.

The protocol used for genome contamination assessment, genome assembly and identification of putative protein-coding genes and their predicted proteins are provided in the Material and methods. This approach identified 18 481 putative gene models (406 of these gene models demonstrated evidence of multiple splice forms according to MAKER [17]), a total gene count similar to the mean (15 946) for other sequenced stramenopiles (figure 1*b*). The number of introns and exons reported by the program Genome Annotation Generator (GAG) was 67 332 and 85 813, respectively, with an average of 3.64 introns per gene and an average exon length of 228 and intron length of 208 bp.

Using the genome assembly, we were able to identify and assemble a hypothetical circular mitochondrial chromosome (electronic supplementary material, figure S2). Further analysis did not identify a candidate relic plastid genome (electronic supplementary material, figure S3), while phylogenomic analysis identified only four genes that, under certain scenarios for gene ancestry, could represent genes acquired as part of the endosymbiosis that gave rise to the plastid organelle present in photosynthetic stramenopiles (electronic supplementary material, figure S3).

### 2.2. Genome size, ploidy and evidence of sexual reproduction

K-mer counting [18] was used to predict a haploid genome size of between 54.1 and 68.6 Mbp with follow-up analysis focusing specifically on the ≥1 kbp assembly suggesting a genome size of 65.7 Mbp across 4758 scaffolds and a scaffold N50 size of 35.57 kbp (L50 of 399). The average sequencing coverage of the total assembly was estimated to be 312×, and the average coverage over the ≥1 kbp scaffolds is 610×. Extraction and purification of long strands of DNA was not achieved using multiple DNA extraction protocols, preventing sequencing using a long-read technology and/or pulsed-field gel electrophoresis to estimate chromosome number. We used a RT-PCR method for estimation of genome size [19] that indicated a haploid genome size of 46.9 Mb (s.e.m. = 1.5).

As mentioned in the methods, the N50 of the genome assembly was much improved by the use of Platanus—an assembly algorithm optimized for multi-ploidy genomes. To further investigate evidence of ploidy in our *H. catenoides* culture, we mapped approximately 101 million reads to the 65.7 Mbp assembly identifying 1 393 505 single nucleotide polymorphisms (SNPs) with 1 332 610 (96%) of the SNPs identified consisting of a two-way nucleotide polymorphism (i.e. 58.8/41.2% mean character split). We also took all scaffolds and plotted SNP frequency against scaffold size. The majority of the scaffolds are clustered around a SNP frequency of approximately 0.0275 (electronic supplementary material, figure S4), suggesting that this variation is consistent and not specific to a subset of chromosomes, for example, in the case of aneuploidy. Interestingly, this analysis showed two large scaffolds with very low SNP frequency compared with the rest of the assembly. These scaffolds contain a number of genes with high sequence identity to genes found on large DNA viruses, suggesting the presence of a viral genome or evidence of a recent viral introgression, discussed further below. K-mer mapping [18] showed two peaks in coverage frequency, which is consistent with the reads mapping to a diploid genome (electronic supplementary material, figure S5).

Using reciprocal BLAST searches, we confirmed that *H. catenoides* encodes and expresses putative homologues of all seven eukaryotic meiosis-specific gene families [20] in the culture conditions used to grow *H. catenoides* (see electronic supplementary material, table S1). To our knowledge, sexual recombination has only been observed once in Hyphochytriomycota cultures, with Johnson [21] identifying cellular forms suggestive of zygote production as a result of fusion in the resting spore development of *Anisolpidium ectocarpii* [21]. However, a range of different sexual reproduction systems have been identified in the oomycetes (e.g. [22]); collectively these data suggest meiosis is present in representative taxa across the wider Pseudofungi.

### 2.3. Phylogenetic position of *Hyphochytrium*

**Figure 2.**
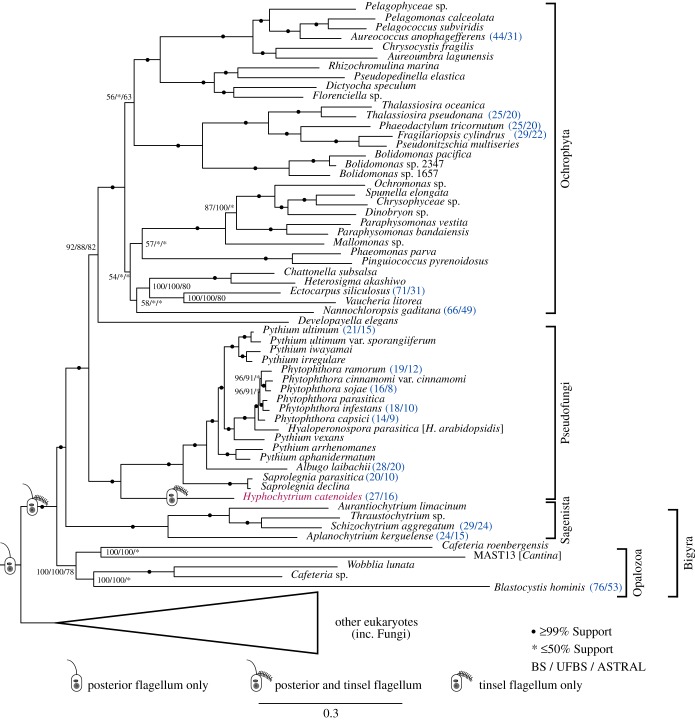
A subsection of the 325 gene (90 230 amino acid) phylogeny of eukaryotes (electronic supplementary material, figure S6a) demonstrating the branching position of *Hyphochytrium*. *Hyphochytrium* highlighted in magenta. The ML tree was built using a supermatrix approach in IQ-TREE under the site heterogeneous model of evolution, LG + *Γ*4 + FMIX(empirical, C60) + PMSF. Values at nodes are ML bootstrap (MLBS) (100 real BS replicates in IQ-TREE LG + *Γ*4 + FMIX(emprical, C60) + PMSF), MLBS under the partitioned dataset using the LG + G4 model of evolution per partition (1000 ultrafast BS replicates) and 100 ASTRAL coalescence multilocus bootstrap replicates, respectively. Bootstrap values below 50% are denoted as an asterisk. Circles denote 99% or above values from all tree topology support analyses. Cartoons of cells indicate change in stramenopile flagellum morphology. Figures highlighted in blue and in parentheses after taxon names are the numbers returned by CEGMA for the complete/partial predicted frequency of 248 CEGs.

*Hyphochytrium* has previously been shown to branch as a sister-group to the oomycetes in rRNA gene phylogenies (e.g. [3,15]). Using a suite of concatenated multiple amino acid sequence alignment approaches (supermatrix and per gene partitioned approaches) and a gene tree coalescence approach [23], we investigated the phylogenetic relationship of *Hyphochytrium* to other eukaryotes by building on previous phylogenomic analyses (e.g. [24–26]). We generated a concatenated amino acid alignment of 325 orthologues (128 taxa and 90 230 amino acid sites) including a comprehensive sampling of eukaryotic taxa based on previously published analyses [24]. We used this alignment to calculate a eukaryote-wide phylogeny using a maximum likelihood (ML) approach with 100 ‘standard’ bootstrap replicates using the IQ-TREE software [27,28] under the site heterogeneous model LG+*Γ*4+F+FMIX (empirical, C60) + PMSF [29] (figure 2; electronic supplementary material, figure S6a shows the wider tree topology). To obtain additional topology support values, we inferred a tree based on this supermatrix with a per gene partitioned model in IQ-TREE with 1000 ultrafast bootstraps replicates (figure 2). Furthermore, using a gene tree coalescence approach in ASTRAL [23] we inferred a species tree with 100 multilocus bootstrap replicates (figure 2). Previously, genes with higher relative tree certainty (RTC) values were shown to improve the overall robustness of phylogenomic analyses [30]. In order to examine the effect of orthologues selected for multi-gene tree analysis, we inferred the RTC for each of the 325 orthologues using RAxML [31], with 100 rapid bootstrap replicates under the LG + *Γ*4 model of evolution. The orthologues were ranked, and the top 50% with the highest RTC scores were selected and multiple gene phylogenies were calculated as above (electronic supplementary material, figure S6b).

The resulting tree topology (figure 2) demonstrates that *H. catenoides* forms a sister-branch to the oomycete radiation with ≥99% support from all methods used for both the 325 multi-gene analysis and the orthologues ranked in the top 50% according to RTC scores (electronic supplementary material, figure S6b). The internode certainty (IC) [30,32] scores of nodes within both analyses showed this phylogenetic relationship was moderately supported across the alignment data matrix (electronic supplementary material, figure S7a,b), consistent with the possibility of mixed signal for this branching relationship in our ‘orthologue’ gene sets. Nonetheless, these results are consistent with the Pseudofungi hypothesis, i.e. the hyphochytriomycetes and the oomycetes are monophyletic and share a common evolutionary trend towards fungal-like osmotrophic feeding and polarized cell growth [3,4].

Our tree places the Pseudofungi as a sister-group to the photosynthetic stramenopiles (i.e. the Ochrophyta) plus *Developayella.* This has some consistencies with previously published phylogenetic analysis based on three nuclear encoded genes [33] and wider phylogenomic analysis [24,34], and in contradiction to analyses of mitochondrial gene phylogenies (concatenation of 10 genes, 7479 positions), which have demonstrated that a separate stramenopile group, the Labyrinthulida (i.e. Bigyra), forms a sister-group to the oomycetes [35]. We note, however, this phylogeny demonstrates a different branching relationship with *Developayella* which is shown here to be sister to the Ochrophyta, a relationship very weakly supported in the internode consistency analyses (electronic supplementary material, figure S7a,b) [32]. The tree recovered here has some similarities to that reported by Derelle *et al.* [34], which uses a large phylogenomic dataset from different taxa. This work argues for monophyly of Bigyra (e.g. *Blastocystis* *+* *Aplanochytrium* and *Schizochytrium*), although our tree shows that this group is paraphyletic, a relationship also shown in Noguchi *et al.* [24]. Derelle *et al.* [34] also recovered paraphyly of this group in a subset of their Bayesian analysis and in their ML analysis, but then went on to demonstrate that this relationship is likely due to a long branch attraction artefact (e.g. [36]) associated with the *Blastocystis* branch and which can lead to the misplacement of Opalozoa (e.g. *Blastocystis*). Interestingly, sisterhood of the Pseudofungi and Ochrophyta implies a minimum of two losses of photosynthesis [34] and independent specialization of ‘osmotrophic lifestyles' in the Bigyra (e.g. *Aplanochytrium* and *Schizochytrium*) and the Pseudofungi (e.g. *Hyphochytrium* and *Phytophthora*) within the stramenopiles. However, this scenario implies that the stramenopile lineage was ancestrally photosynthetic [37], a subject of debate [38,39] (electronic supplementary material, figure S3).

### 2.4. Shared derived traits across the Pseudofungi

Given the placement of *H. catenoides* as a sister-branch to the oomycetes, we were interested in investigating the conservation of cellular, biochemical and genetic traits shared across pseudofungal taxa*.* Oomycete plant parasites, e.g. *Phytophthora* spp., are sterol auxotrophs and appear to have lost the enzymes involved in sterol biosynthesis [40]. The sterol biosynthesis pathway has been predicted to function in *Saprolegnia*, and a putative CYP51 sterol-demethylase encoding gene was identified from the *Saprolegnia parasitica* genome and transcriptome data [12,41]. The protein encoded by this gene is a target of antimicrobial drugs such as clotrimazole and, therefore, has been suggested as a therapeutic target for treatment of *Saprolegnia* infections of fish [42]. Reciprocal BLASTp searches and phylogenetic analyses demonstrated that *H. catenoides* also possesses a putative orthologue (Hypho2016_00003038; electronic supplementary material, figure S8a) of the *S. parasitica* CYP51 sterol-demethylase, which appears to be lost in plant parasitic oomycetes. To confirm that this is a viable drug target we grew *H. catenoides* in the presences of two azole ‘antifungals’—clotrimazole and fluconazole—to assess effectiveness of these compounds in inhibiting *H. catenoides* growth. Both ‘antifungal’ agents were able to inhibit growth of *H. catenoides* (MIC_100_: clotrimazole 0.25 µg ml^−1^; fluconazole 4 µg ml^−1^; electronic supplementary material, figure S8b), indicating that the *H. catenoides* is susceptible to azole compounds, consistent with *H. catenoides* having a functional CYP51 enzyme.

There has been considerable effort to sequence a number of oomycete genomes, which has largely focused on parasitic taxa (e.g. [11,12,43–46]). This work has also, in part, focused on identifying candidate effector proteins (secreted proteins that perturb host function for the benefit of the invading parasite [47] and which often contain N-terminal RxLR amino acid motifs [48–50]) or lectin proteins that bind host molecules. Searches of the *H. catenoides* genome demonstrate there is only one putative protein of unknown function with a candidate RxLR motif (table 1). In addition, *H. catenoides* lacked several gene families linked with the evolution of plant parasitic traits in the oomycetes, i.e. NPP1 or NEP-like proteins (necrosis-inducing *Phytophthora* protein [51,52]), elicitin proteins [53], cutinase [54], pectin esterase and pectin lyase [55,56]. The animal parasite *S. parasitica* was noted to show enrichment of Notch proteins and Ricin lectins, as well as presence of other galactose-binding lectins and the bacterial toxin-like gene family (haemolysin E) [12]. While the Notch protein and Ricin lectin gene families are present in *H. catenoides*, they show no evidence of enrichment comparable to *S. parasitica*. The galactose-binding lectin and haemolysin E gene families are absent. Protease gene families show no general enrichment in comparison with other stramenopiles (table 1).

**Table 1.** Comparison of pseudofungal/stramenopile genes with generalized function.

gene families	*Hyphochytrium catenoides*	*Albugo laibachii*	*Hyaloperonospora arabidopsidis*	*Phytophthora infestans*	*Phytophthora ramorum*	*Phytophthora sojae*	*Pythium ultimum*	*Saprolegnia parasitica*	*Ectocarpus siliculosus*	*Thalassiosira pseudonana*
RXLR	1	0	23	317	102	106	0	0	0	0
NPP1-like proteins	0	0	21	27	62	74	7	0	0	0
elicitin	0	9	14	43	47	53	44	25	0	0
*plant cell wall degrading*										
cutinase	0	3	2	4	4	16	0	0	0	0
glycosyl hydrolases	357	384	242	533	838	1208	436	415	282	264
pectin methyl esterases	0	0	4	11	13	19	0	0	0	0
pectate lyase	0	0	8	36	25	24	16	0	0	0
polygalacturonase	0	3	3	24	17	25	6	3	0	0
*lectins*										
PAN lectin	4	3	2	5	8	5	11	6	1	0
ricin lectin	1	1	3	5	9	10	5	57	0	1
jacalin lectin	2	0	8	15	23	15	3	4	1	0
galactose lectin	0	0	1	1	1	1	1	1	1	1
leguminous lectin	2	1	0	2	1	1	2	0	2	1
legume-like lectin	2	3	3	3	3	3	3	3	3	0
*protease functions*										
protease inhibitors	13	11	14	51	35	46	30	28	15	15
proteases, all	428	379	324	450	541	602	482	630	361	367
serine proteases	166	84	106	170	182	189	200	248	112	140
metalloproteases	92	91	80	98	100	91	107	129	88	101
cysteine proteases	115	124	92	140	116	113	121	208	117	85
*others*										
ABC transporters	81	36	49	148	171	175	158	138	70	58
protein kinases	243	305	217	423	398	430	232	690	330	160
Notch protein	3	0	1	1	1	1	1	18	11	2
haemolysin E	0	0	0	0	0	0	0	5	0	0

**Figure 3.**
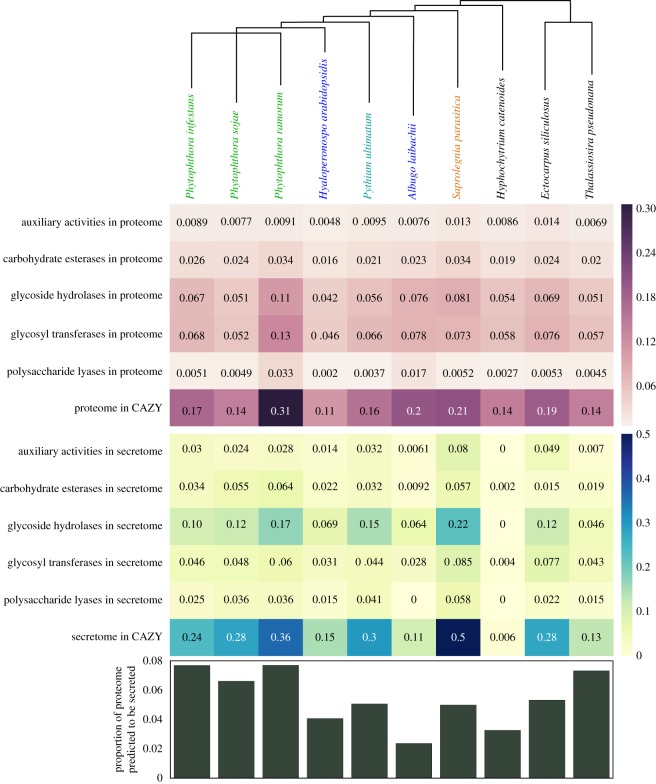
Comparison of secreted proteome and putative carbohydrate active proteins across the Pseudofungi including photosynthetic stramenopile taxa as an outgroup. The schematic phylogeny at the top indicates the relationship between different oomycete species with the ‘lifestyle’ of each species indicated by text colour; green (*Phytophthora* species) indicates plant hemibiotroph, blue (*Hyaloperonospora* and *Albugo*) obligate plant biotroph, teal (*Pythium*) plant necrotroph, orange (*Saprolegnia*) animal saprotroph/necrotroph and black indicates putatively free living (e.g. *Hyphochytrium, Ectocarpus* and *Thalassiosira*). The first heat map in white/purple indicates the proportion of proteome of each organism which was identified as belonging to a particular CAZY (www.cazy.org) category using BLASTp with an expectation of 1 × 10^−5^. The number listed is the proportion, and the colour relates to magnitude of the listed number (as shown by scale bar). The second heat map, in blue/yellow, indicates the proportion of the secretome (predicted via a custom pipeline https://github.com/fmaguire/predict_secretome/tree/refactor) that is identified as belonging to each of these CAZY categories. Auxiliary activities (AA) cover redox enzymes that act in conjunction with CAZY enzymes. The bar chart at the bottom shows the proportion of the proteome for each organism which is predicted to be secreted.

Comparative analysis of candidate secreted proteins defined by *in silico* identification of putative N-terminal secretion sequences demonstrated that *H. catenoides* contains a lower proportion of secreted proteins compared with many other stramenopiles, comparable with the paraphyletic obligate biotrophs *Albugo laibachii* and *Hyaloperonospora arabidopsidis* (figure 3)*.* The *H. catenoides* predicted proteome contains a moderate-to-low proportion of carbohydrate active enzymes [57] relative to other stramenopiles. Interestingly, *H. catenoides* has very few secreted carbohydrate active enzymes in comparison with other stramenopiles, suggesting that *H. catenoides* has a low diversity of extracellular carbohydrate processing functions and is, therefore, dependent on a limited subset of extracellular sources of fixed carbon (figure 3). To test this observation, we grew *H. catenoides* cultures in 190 different carbon sources using OmniLog PM1 and PM2 plates, which allows investigation of growth and respiration rate across a diversity of different carbon sources [58]. These data demonstrated (electronic supplementary material, figure S9a,b) a significant increase in respiration rate compared with the controls upon the addition of: α- or β-cyclodextrin (*p* = 0.01 and 0.01), dextrin (*p* = 0.02), Tween 40 or 80 (*p* = 0.03 and 0.03) or melibionic acid (*p* = 0.03). Of note, dextrin/cyclodextrins are products of enzymatic activity upon starch, a typical component of *H. catenoides* growth medium (YpSs), and may be indicative of the environment in which this organism is typically found. The addition of Tween 40 or Tween 80 has been shown to improve yield in other organisms [59] and may result from direct accumulation of fatty acids, or altered membrane permeability affecting nutrient uptake. In contrast to many oomycetes (e.g. [60]), *H. catenoides* demonstrates a limited utilization of diverse carbon sources. These data are consistent with the hypothesis that the evolution of a wide diversity of secreted carbohydrate active enzymes is associated with evolution of parasitic lifestyle within the oomycete lineages (e.g. [12,61–63]), although this pattern could also be the product of secondary loss in the *H. catenoides* branch.

Seidl *et al*. [64] detected 53 domain architectures that were unique and conserved across the oomycetes *P. infestans*, *P. ramorum*, *P. sojae* and *Hy. arabidopsidis.* Domain architectures are often recombined by a process of gene fusion and/or domain ‘shuffling’ [65]. Such gene fusion characters, although subject to sources of homoplasy (such as gene fission [66]), can represent synapomorphic traits useful for polarizing phylogenetic relationships. We searched the *H. catenoides* genome for evidence of the 53 gene fusions previously identified in oomycetes [64] and found that 12 of these domain architectures were also present in *H. catenoides* (electronic supplementary material, table S2). Of note, we found a fusion gene of a putative β-glucan synthase enzyme domain and a putative membrane transporter gene (electronic supplementary material, table S2 and GenBank ‘nr’ protein database) shared across the Pseudofungi, suggesting that domain fusion has led to a unique coupling of substrate transportation and enzymatic processing prior to the radiation of this group. Theoretically, however, without proteomic data we cannot exclude the possibility that this novel domain combination may be the product of a conserved operon-like gene structure.

Using OrthoMCL [67] combined with a custom pipeline we identified nine Pseudofungi-specific orthologues, with five of these orthologues representing additional Pseudofungi-specific domain combinations (electronic supplementary material, table S3). Of note, these combined results (electronic supplementary material, table S2 and S3) demonstrate a novel diversification of the serine/threonine kinase gene families, consistent with expansions of kinase encoding gene families present in oomycete genomes [12].

### 2.5. Protein repertoire changes associated with loss of the posterior flagellum

**Figure 4.**
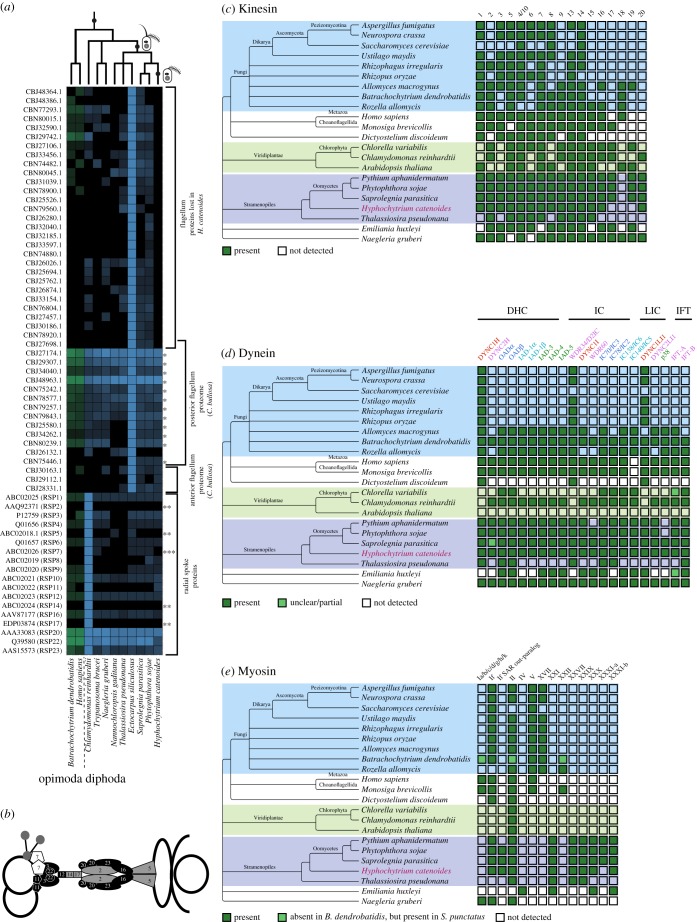
Comparative genomic analysis of *H. catenoides* flagellum proteome and motor protein repertoire. (*a*) Heat map showing sequence identity profiles for flagella proteins with putative homologues present across the eukaryotes (see, electronic supplementary material, table S4 for full dataset). The heat map identifies 29 proteins present in the oomycetes but absent in *H. catenoides*, suggesting that this gene had been lost at the same proximate point to the loss of the posterior flagellum. The analysis also shows 12 proteins (marked as *) identified as posterior flagellum specific in *C. bullosa* that are retained in *H. catenoides* and therefore putatively function in the anterior flagellum. Three *C. bullosa* anterior flagellum specific proteins are also retained in *H. catenoides*. The putative radial spoke proteome also shows numerous losses similar to *Ho. sapiens* (**), this includes the loss of RSP7 (***). Only changes in flagella cytology relevant to the evolution of the stramenopiles are sketched on the top tree. (*b*) Shows a cartoon of the radial spoke protein complex identified in *Chlamydomonas* with each shape number referring to the RPS number [70]. Black shapes illustrate proteins of the spoke complex conserved across the eukaryotes sampled, grey are non-conserved proteins (showing evidence of mosaic loss), while the white complex refers to RPS7 which, although absent in *Ho. sapiens* and other eukaryotes, has been lost separately and is consistent with the loss of the posterior flagellum in the ancestor of *H. catenoides*. (*c*) Distribution of major kinesin paralogue families. Kinesin-2, -9, -16 and -17 have been suggested to have function associated with the flagellum [71]. (*d*) Distribution of major dynein paralogue families. Paralogues are grouped according to the class of component: dynein heavy chain (DHC), intermediate chain (IC), light-intermediate chain (LIC) and intraflagellar transport (IFT), and coloured according to function (red, cytoplasmic; magenta, IFT; dark blue, axonemal outer-arm; light blue, axonemal inner-arm; green, axonemal single-headed). (*e*) Distribution of major myosin paralogue families focusing on variation between Fungi and Pseudofungi.

The stramenopiles (also known as Heterokonta, meaning possessing two unequal flagella) were formally described as a phylum based on the presence of two motile flagella: a ‘standard’ smooth posterior flagellum and an anterior flagellum with tripartite rigid tubular mastigonemes (tinselate) [2]. *Hyphochytrium* builds only a single, anterior tinselate flagellum [6] while the oomycetes build the stramenopile flagella pair. Therefore, the posterior smooth flagellum was lost in the ancestor of the hyphochytrids (figure 2). To explore the consequence of the loss of this organelle in *H. catenoides*, in terms of gene/protein repertoire, we used a comprehensive list of proteins putatively associated with flagellar function [68] to survey the *Hyphochytrium* genome. This list comprises 592 amino acid sequences, 355 of which are found in both the major eukaryotic phylogenetic groupings of Opimoda and Diphoda [69], suggesting they are universal flagellar proteins (UFPs; electronic supplementary material, table S4, figure 4*a*); 330 of the 355 UFPs are also present in the predicted proteome of *H. catenoides*, suggesting that the majority (93%) of the UFPs have been retained and are likely to encode a function associated with the anterior tinselate flagellum.

Flagellum-specific proteomic analysis of the stramenopile brown alga *Colpomenia bullosa* identified 14 proteins specific to the posterior flagellum and three specific to the anterior flagellum [68]. BLAST searches suggest that the three anterior flagellum proteins are also present in *H. catenoides*, as are 12 of the 14 posterior flagellum proteins identified from *C. bullosa*. Conservation of these ‘posterior-specific’ proteins suggests that they have functions associated with the anterior tinselate flagellum in *H. catenoides* (figure 4*a*)*.* One of the *C. bullosa* posterior-specific flagellum proteins absent in *H. catenoides* and the oomycetes is the PAS/PAC sensor hybrid histidine kinase (also known as a helmchrome, CBJ26132.1), a putative photo-sensor associated with a swelling in the posterior flagellum of brown algae [68], discussed further below.

Twenty-nine of the UFPs (8%) were present in oomycetes and other eukaryotic groups but absent in *H. catenoides*. These may represent genuine gene losses, although absences in our draft genome may also be due to incomplete genome sequencing and assembly. If these are genuine losses, it suggests they represent UFP losses that correlate with loss of the posterior flagellum without the function of these UFPs being integrated into the anterior tinselate flagellum (figure 4*a*)*.* These losses include a putative homologue of the Dynein Regulatory Complex 1 (DRC1) protein, which regulates inner dynein motor activity in *Homo sapiens* and *Chlamydomonas reinhardtii* [72], and Radial Spoke Protein 7 (RSP7), a protein that functions in flagellum structure and beating in *Ch. reinhardtii* [70]. Further, analysis of the radial spoke protein repertoire encoded by *H. catenoides* identified a number of other components of the radial spoke complex which are putatively absent in *H. catenoides*. However, RSP7 was the only radial spoke proteome loss specific to the loss of the posterior flagellum in the *Hyphochytrium* lineage (figure 4*a*,*b*); this protein is putatively encoded in the oomycetes but has been separately lost within the Opisthokonta (e.g. *Ho. sapiens*)*.* In *Chlamydomonas* [70], RSP11 and RSP7 have been shown to contain a RIIa domain [73]. Association between RIIa and AKAP domains and RSP3 at the spoke stalk is suggested to be important for flagellar function [70]. Interestingly, comparative analysis suggests that neither RSP7 nor RSP11 are conserved across flagellum-bearing eukaryotes with only *Chlamydomonas*, *Batrachochytrium* and *H. catenoides* retaining RSP11 in our comparative dataset (figure 4*a*,*b*)*.* Domain analysis [74] of the putative *H. catenoides* RSP3 and RSP11 confirmed these proteins contain an AKAP and a RIIa domain, respectively, suggesting that *H. catenoides* has retained only RSP3–RSP11 protein–protein interaction at the base of the radial spoke, proximate to the outer doublet (figure 4*b*).

Phylogenomic analysis of motor protein repertoire, specifically kinesins and dyneins (figure 4*c*,*d*), confirmed that the *H. catenoides* genome assembly has retained many of the motor proteins associated with flagellum function. These include representatives of all seven axonemal dynein heavy chain families (plus their associated intermediate and light-intermediate chains) [75], both the retrograde (DYNC2) and anterograde (Kinesin-2) motors used in intraflagellar transport (IFT), and non-motor components of the IFT particles (figure 4*c*). Also identifiable are members of Kinesin-9 and -16 families, which are present in organisms which build motile flagella [71] (figure 4*d*). This motor repertoire is similar to that seen in oomycetes and shows that the modified tinselate *H. catenoides* anterior flagellum has retained most functions associated with flagellar motors. Wickstead & Gull have also proposed that the Kinesin-17 family has a flagellar function based on its phylogenetic distribution [71]. Our analysis suggests that *H. catenoides* has lost Kinesin-17 (unlike in the oomycetes). This may be associated with the loss of the posterior smooth flagellum, but may also be due to missing sections of the genome in the draft assembly.

### 2.6. Photoreceptors

Stramenopile species have been shown to encode a range of photoreceptor proteins and to initiate a series of responses to light including phototaxis [76]. Specifically, the zoospores of some stramenopile algae can show positive and negative phototaxis [77] associated with a flavoprotein photoreceptor [78], putatively the ‘helmchrome’ located in the posterior flagellum [68] and associated with ‘flagellar swelling’ and a stigma [77]. Consistent with the loss of the anterior flagellum, *H. catenoides* (figure 4; electronic supplementary material, S10) also lacks a gene putatively encoding a helmchrome protein.

A number of additional putative photo-responsive proteins have also been reported from *Ectocarpus* [10]. Using these data and other seed sequences (e.g. [68,79]), we searched the *H. catenoides* genome for putative homologues of photo-responsive proteins. Reciprocal BLAST searches demonstrated that the *H. catenoides* genome contained putative homologues of the flavoproteins Cryptochrome (Hypho2016_00016188), Cryptochrome DASH (Hypho2016_00004514) and Photolyase (Hypho2016_00002462) gene families (electronic supplementary material, figure S10a), and transcriptome data demonstrate that these genes are transcribed. This analysis also identified three putative type I (microbial) rhodopsins (Hypho2016_00006030, Hypho2016_00006031 and Hypho2016_00010050), the first putative representative of this gene family from a stramenopile (electronic supplementary material, figure S10a,b). The three rhodopsins all contain a conserved 11-*cis*-retinal binding pocket, specifically the lysine residue site of the Schiff base where the retinal is covalently linked (electronic supplementary material, figure S10c). Furthermore, reciprocal BLAST searches of both the genome and the transcriptome sequence datasets confirmed the presence of genes putatively encoding the latter two steps of the retinal biosynthesis pathway (e.g. a putative β-carotene-15, 15′-dioxygenase (Hypho2016_00004122) and a putative retinol dehydrogenase (Hypho2016_00000702). These genes encode the pathway steps that convert the vitamin β-carotene into 11-*cis*-retinal, the critical cofactor for rhodopsin to function as a light-responsive protein.

### 2.7. Gene families encoding hallmarks of fungal characteristics in the Pseudofungi

**Figure 5.**
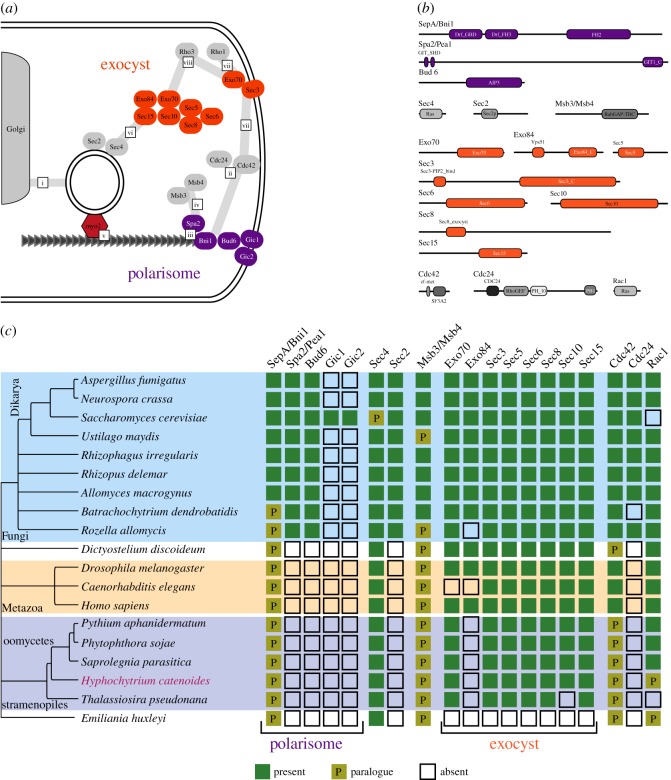
Comparative genomic analysis of gene families that function in polarized filamentous growth in the Fungi. (*a*) Cartoon outlining proteins and complexes involved in polarized growth in *Saccharomyces cerevisiae* (this is a variation of a figure shown in [80]). Vesicles are delivered from the Golgi (*a*(i)) along cytoskeleton tracks to predetermined sites on the plasma membrane. Cdc42p is activated by Cdc24p (*a*(ii)) promoting [84] assembly of the polarisome complex (*a*(iii)) resulting in the formin Bni1p radiating actin cables [85,86]. Msb3p and Msb4p interact with Spa2 in the polarisome (*a*(iv)) which is thought to recruit Cdc42 from the cytosol at the site of tip growth [87]. Post-Golgi secretory vesicles are transported along actin cables using a type V myosin motor protein [88,89] (*a*(v)), to dock with the exocyst complex in a process dependent on Sec4 and its GEF Sec2 [90,91] (*a*(vi)) and so the vesicle is guided to its target site on the plasma membrane [92]. Cdc42p and Rho1 are required for localization of Sec3p, which together form a spatial marker for the exocyst (*a*(vii)) and Rho3p and Cdc42p mediate vesicle docking (*a*(viii)). Cdc42p plays a key role in regulating these processes in *S. cerevisiae* but in Pezizomycotina and basidiomycete fungi equivalent functions are performed by Rac1p [93,94]. (*b*) The domain architecture of the 17 proteins associated with polarized growth in fungi. (*c*) The taxon distribution of putative homologues of polarized growth proteins across a representative set of taxa including the Pseudofungi. ‘P’ indicates a putative paralogue relationship as identified using phylogenetic analysis.

One of the main purposes for sequencing the *H. catenoides* genome was to investigate conservation and/or loss of genes that underpin the fungal/pseudofungal lifestyle. Many fungi grow as filamentous cells, reinforced by robust cell walls composed of polysaccharides such as chitin. These characters are not unique to the Fungi but are typical in many fungal lineages [80]. A suite of cellular systems allow fungi to grow as polarized cells, laying down cell wall and feeding on extracellular substrates by a combination of exocytosis of enzymes and cell-wall material combined with endocytosis and transporter protein mediated uptake of target nutrients. Fungal filamentous structures such as hyphae grow almost exclusively from the tip of the hyphal structure [81], allowing fungi to ‘grow as they feed’. This feature combined with a robust cell wall means they can generate high turgor pressures, ramify into recalcitrant material, feed osmotrophically and maximize metabolic rates [80,82,83]. Homologous cellular systems also drive bud growth in *Saccharomyces cerevisiae*, allowing researchers to use *S. cerevisiae* to study proteome function involved in polarized growth (for reviews, see [81,84]). The proteins that are known to control this system are illustrated in figure 5*a* and involve key complexes, the exocyst and the polarisome. These systems are important for establishing the temporal and spatial control of polarized cell growth in fungi [81,84]. Comparative analyses show the exocyst and Sec4 orthologues are conserved across a diversity of eukaryotes including *H. catenoides*, while the polarisome and associated proteins are specific to the Fungi, given current taxon sampling (figure 5*c*). Comparative analysis demonstrates that specific elements of polarized cell growth control are not present in Pseudofungi, suggesting these filamentous microbes accomplish polarized growth using different proteome functions.

Motor protein evolution has been suggested to be an important factor in the acquisition of filamentous growth phenotypes in the fungi, with a specific focus on myosin and kinesin genes that encode functions involved in polarized cell growth, vesicle-transit and chitin synthesis [95–97]. Phylogenomic analysis of the motor head domain of all three motor types (figure 4*c*–*e*) demonstrates no expansion in motor paralogues uniquely shared by the Fungi and Pseudofungi. In addition, Pseudofungi lack the Myosin V and XVII shown to be important in fungal growth and chitin synthesis [96] (figure 4*e*). The lack of shared/unique motor repertoire between Fungi and Pseudofungi is consistent with the idea that these groups evolved filamentous polarized growth characteristics separately and based on different cellular systems. It has been noted that oomycetes contain a diverse complement of myosin paralogues [98]. The analyses reported here demonstrate that elements of this oomycete motor protein gene family expansion are also present in *H. catenoides*, specifically; Myosin XXX and XXI and Kinesin 14 and 20 show high degrees of expansion by duplication specific to the Pseudofungi (figure 4*c*,*e*), suggesting these motor proteins may be linked to filamentous polarized growth characteristics present in this group.

**Figure 6:**
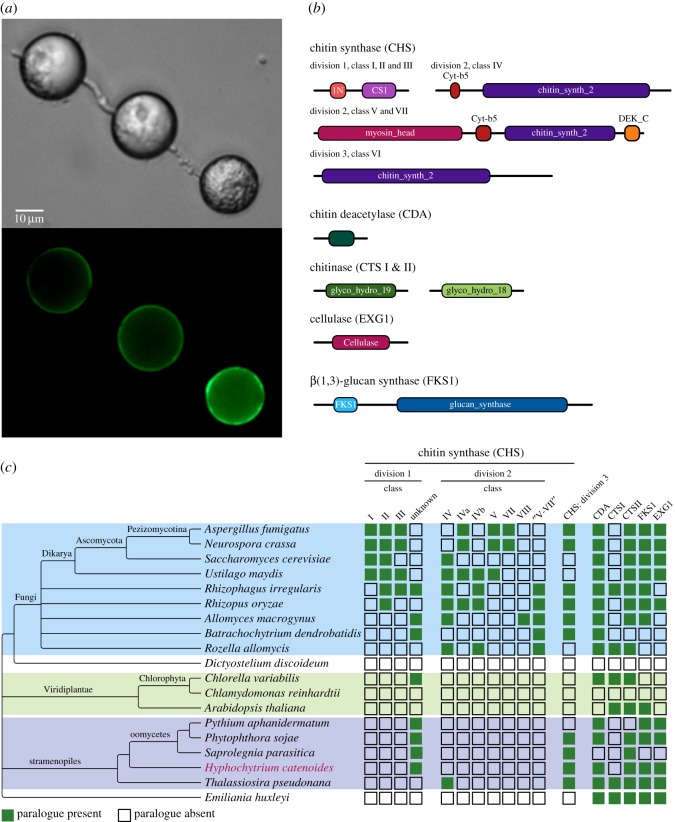
Comparative genomic analysis of gene families that function in cell-wall synthesis. (*a*) Micrographs showing the wheat germ agglutinin fluorescent staining of a chitin cell wall on *Hyphochytrium* structures. (*b*) The domain architecture of eight proteins that function in cell-wall synthesis. (*c*) The taxon distribution of putative gene families associated with cell-wall synthesis across a representative set of taxa including the Pseudofungi.

Like fungi [99] and many other eukaryotes [100–106], *H. catenoides* also produces chitin as cell-wall material [107]. Oomycetes have also been shown to produce chitin in their cell walls [108]. This is consistent with previous data that suggest that chitin synthesis and deposition as a cell-wall material predates the diversification of many major lineages of the eukaryotes [80,107]. *H. catenoides* has a similar repertoire of chitin synthesis and digestion as found in the oomycetes (i.e. chitin synthase division I), while another group of stramenopiles, the diatoms, which also produce chitin [109], have a variant chitin gene repertoire, namely chitin synthase division II and a chitinase (GH19) not present in Pseudofungi (figure 6). This suggests that chitin production as a cell-wall component is universal and anciently acquired in the eukaryotes, but the genes that control the synthesis and remodelling of this structural polysaccharide have been reconfigured numerous times. Specifically, Pseudofungi seem to lack all chitin synthase division II genes (figure 6*c*), which are numerous and diversified in fungi, suggesting another key difference between the Fungi and Pseudofungi.

### 2.8. Viral integration across the Pseudofungi

**Figure 7.**
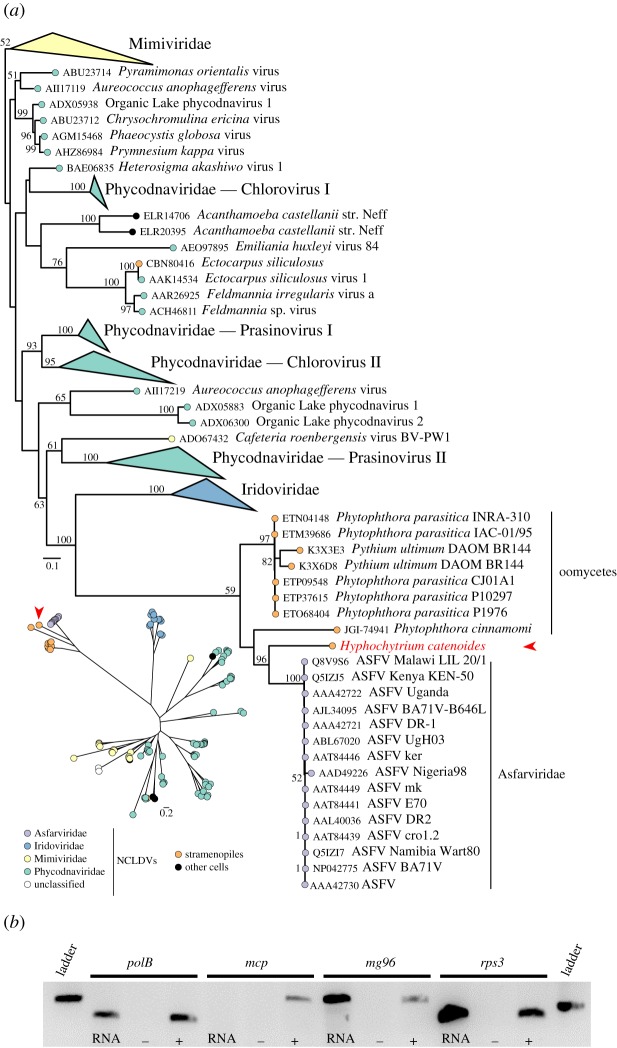
Phylogeny of viral MCP proteins indicating the branching position of the pseudofungal genes and evidence of transcription of viral derived genes in *H. catenoides*. (*a*) Homologous sequences were identified using three psi-BLAST iterations with *H. catenoides* putative MCP as query; to remove sequence redundancies, retrieved sequences were clustered at 90% amino acid identity with cd-hit v4.6. Sequences were then aligned using MAFFT v7 iterative, global homology mode (G-INS-i); alignment sites retained for subsequent phylogenetic analysis were selected using trimAL [110] gap distribution mode. Final MCP multiple sequence alignment was composed of 386 sites. ML tree was inferred using IQ-TREE v1.3 and LG + I + *Γ*4 + F model (determined as the best-fitting model by Bayesian information criterion). Node supports were evaluated with 100 non-parametric bootstrap replicates. The Mimiviridae clade was used to root the ML tree (unrooted version displayed on the lower left part). (*b*) RT-PCR showing expression of *polB* and *mg96* viral genes alongside an *rps3* positive control. No expression of the *mcp* gene was detected. RT-PCR was performed on *H. catenoides* RNA alongside genomic DNA (+) and no-template (−) controls, with PCR products run on an agarose gel alongside a 1 kb ladder (Promega; 250 bp shown).

The comparative genomic analysis of Pseudofungi demonstrated that *H. catenoides*, *Phytophthora cinnamomi, Phytophthora parasitica* and *Pythium ultimum* harbour genes putatively encoding viral major capsid proteins (MCP) (electronic supplementary material, table S5). These proteins have high sequence identity with each other and branch together with MCP proteins from African swine fever virus (Asfarviridae, a lineage of the nucleocytoplasmic large DNA viruses—NCLDVs), but which are divergent when compared with other NCLDV MCP proteins (figure 7*a*). Exploring the *H. catenoides* genome assembly to determine the presence of viral-like genes, we identified 45 candidate viral-derived genes, 38 of which are present on two scaffolds which were shown to have very low SNP frequency in the assembly (electronic supplementary material, table S5). All of these 38 genes showed highest similarity to NCLDV families such as Mimiviridae, Marseilleviridae, Phycodnaviridae, Asfarviridae and Poxviridae (electronic supplementary material, table S5). The genome assembly in these regions was confirmed by nested PCR and sequencing from both the 5′ and 3′ ends of the *polB*, *mcp*, *mg96* genes of viral ancestry (electronic supplementary material, table S6). The viral-like genes were found in linkage with genes of *H. catenoides*/pseudofungal ancestry. For example, the genome assembly demonstrated that the viral-like *mcp* gene was on the same DNA contig as a putatively native *H. catenoides* histone-encoding gene (electronic supplementary material, figure S11). To confirm this assembly and linkage between ‘host’ and viral gene we conducted a bridging PCR resulting in an amplicon of 2837 bp and sequenced this amplicon, confirming that the *mcp* and histone genes are linked and on the same stretch of DNA (electronic supplementary material, table S6).

One hundred and forty-five predicted genes were identified in the two contigs that contain a high number of viral genes. BLASTx analyses suggest that the two contigs contained 37 (26%) and 18 (12%) genes of highest identity to genes of known viral genomes (electronic supplementary material, table S7). The BLASTx results for the remaining 235 putative genes showed a wide variation of top scoring hits including both prokaryotic- and eukaryotic-like genes. The frequency of putative exons for the two contigs was 1.62 and 1.49, respectively, a lower intron/exon frequency than observed for the wider genome (intron frequency = 3.64), thus suggesting that genes encoded on viral gene-containing contigs have introns. Indeed, multiple viral-like genes show evidence of introns suggesting these genes have been: incorrectly modelled, subject to intronization or exon-like shuffling during integration, or these genes are undergoing pseudogenization and are therefore broken ORFs, which are being reported as intron/exon structures. However, we note that gene of viral provenance Hypho2016_00000945-RA (scaffold 5419) contains multiple putative coding regions present in our transcriptome data. The low SNP frequency of these contigs suggests they represent a unique haploid portion of the genome, a viral genome captured in our assembly, or alternatively a site of viral introgression in the *H. catenoides* genome. We currently favour the hypothesis that this is a site of viral introgression due to the presence of putative introns in the contig and the low relative proportion of genes of clear viral provenance.

Products from *polB, mg96* and *rps3* were detected by RT-PCR in our culture conditions, suggesting that viral-like genes are transcriptionally active (figure 7*b*)*.* By contrast, a lack of transcript from the *mcp* gene suggests that a complete virus or a viral factory is not being manufactured in the culture conditions tested (figure 7*b*). Electron microscopy also failed to observe icosahedral structures typical of NCLDV particles or an intracellular viral factory (see electronic supplementary material, figure S12).

These data combined with evidence of viral genes present in oomycete genome assemblies (figure 7*a*) [111] suggest a hitherto unsampled diversity of large DNA viruses found infecting or integrated within the genomes of Pseudofungi. This is consistent with other data suggesting the Pseudofungi have been subject to viral transduction [111]. It has also been shown that many different lineages of the stramenopiles have similarly retained fragments of viral genomes [112], suggesting a wider and undersampled diversity of stramenopile-infecting large DNA viruses. It is tempting to speculate that this may be a mechanism driving horizontal gene transfer (HGT) seen in the oomycetes [113], given that NCLDVs have been shown to harbour host-derived and foreign genes [114,115] and that fragments of large DNA viruses have now been shown to be present in fungi [111], a group shown to be a donor of HGT genes to the oomycetes [63,113]. Consistent with this, we note that the two contigs containing the viral derived genes also contain two genes with top BLASTx hits to fungal genes (electronic supplementary material, table S7).

The Pseudofungi are thought to lack the capabilities to perform phagotrophy [4], a mechanism hypothesized to be important for HGT in eukaryotes [116]. However, there is evidence of gene transfer into the oomycetes from both fungi and prokaryotes [54,63,117–121]. The extent of ancient HGTs in eukaryotes has recently been questioned [122]. Yet, Ku *et al*. [122] also identified genes uniquely present in oomycetes and bacteria which are described as ‘recent lineage specific acquisitions' (see fig. 1 in [122], marked as *b*). Evidence of viral introgression within the Pseudofungi, therefore, identifies a possible mechanism driving HGT in the Pseudofungi, which cannot perform phagotrophy. It is important to note that viral transduction as a vector for HGT in the eukaryotes would be likely to produce a very different profile of gene transfer compared with mechanisms such as phagocytosis (in eukaryotes) [116], transformation (prokaryotes and eukaryotes) [123] or conjugation (prokaryotes and eukaryotes) [124,125]. This is because gene transfer via a virus would be likely to transfer a lower number and lower diversity of gene families for two reasons: (i) genes carried by the virus would have been passaged by selection within the viral lineage and (ii) the limited DNA carrying capacity of the viroid. Such a mechanism of HGT is, therefore, consistent with the results of Ku *et al*. [122], which suggest HGT is less frequent in eukaryotes compared with prokaryotes. However, this does not exclude the possibility that infrequent HGTs can lead to the acquisition of novel and/or positively selected traits.

## 3. Conclusion

**Figure 8.**
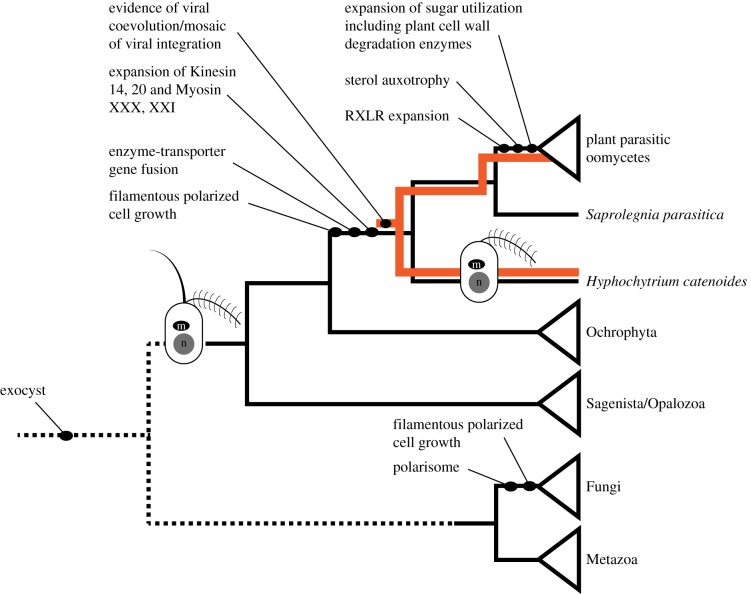
Schematic phylogenetic tree summarizing the evolution of cell and genomic characters relevant to the evolution of the Pseudofungi. Only changes in flagella complement relevant to the evolution of the stramenopiles are sketched.

The draft genome of the free-living stramenopile pseudofungus *H. catenoides* provides an important reference for comparative biology specifically with a view to understanding the evolution of filamentous growth and osmotrophic feeding. *H. catenoides* branches sister to the oomycetes that contains many important parasitic groups. These data demonstrate that *H. catenoides* does not encode many of the gene families found in oomycetes that have been associated with parasitic function, suggesting that these characteristics are more recent adaptations/acquisitions within the oomycetes (table 1). Our data also demonstrates that *H. catenoides*, and the Pseudofungi more widely, possess the genes that encode a range of features associated with filamentous growth and osmotrophic feeding in fungi. These include the exocyst vesicle trafficking system, sterol biosynthesis pathway and a repertoire of chitin cell-wall synthesis systems common to fungi. By contrast, Pseudofungi do not possess the genes encoding a polarisome complex, chitinase I, chitin synthase II/Myosin V or Myosin XVII, identifying clear differences between these two filamentous osmotrophic groups. Figure 8 summarizes how various features associated with filamentous growth and osmotrophic feeding arose relative to the branching position of the Fungi and the Pseudofungi. We hope the *H. catenoides* draft genome will provide a useful dataset for comparative biology within the Pseudofungi and across the eukaryotes, especially with regards to understanding the evolution of filamentous osmotrophic characteristics.

## 4. Material and methods

### 4.1. Cell culture in preparation for sequencing

*Hyphocytrium catenoides* (ATCC 18719) was inoculated onto Emerson YpSs agar. Cell mass was prepared for DNA and RNA extraction as described previously [63]. DNA samples were checked for contamination using an environmental DNA SSU PCR approach [63] using both eukaryotic 18S PCR primers 1F (CTGGTTGATCCTGCCAG) and 1520R (CTGCAGGTTCACCTA) (e.g. [126]), which produced a clean chromatogram of a *Hyphochytrium* 18S sequence, and prokaryotic 16S PCR primers PA (AGAGTTTGATCCTGGCTCAG) and PH (AAGGAGGTCATCCAGCCGCA) which were negative (e.g. [127]).

### 4.2. Genome and transcriptome sequencing, assembly and validation and ORF calling

One lane of paired-end (100 bp) Illumina HiSeq data was generated along with two lanes of paired-end (76 bp) Illumina GAiix at the Exeter Sequencing Service producing 2× 212 760 559 HiSeq reads along with 2× 15 266 599 and 2× 16 274 715 GAiix reads. After trimming and cleaning (using TagCleaner [128] and PRINSEQ [129]) of the data, we subsequently digitally normalized it with khmer [130] in order to discard redundant data and sampling variation and remove errors. This reduced the number of reads to 415 241 668 HiSeq along with 28 964 302 and 30 961 514 GAiix, a reduction of 13 436 262 reads in total. The raw reads are deposited in NCBI and EBI with accessions as: Illumina GAiix = SRX033129 and Illumina HiSeq = ERS1151585 respectively. An initial assembly, using the program Ray v.2.2.1 [131] was generated (see https://github.com/guyleonard/hyphochytrium/tree/master/manuscript/data for details of commands used), and produced 29 448 scaffolds, with a total of 107 387 882 bp, and an N50 of 8 746 bp.

Next, we investigated the possibility that *H. catenoides* was a diploid using an assembly program that allows for multiple ploidy. The program Platanus v.1.2.1 was used to produce an assembly with 53 358 scaffolds incorporating 68 330 525 bp and an N50 of 29 450 bp (see https://github.com/guyleonard/hyphochytrium/tree/master/manuscript/data for details of commands used).

The Platanus assembly was subsequently filtered into four datasets; all scaffolds, scaffolds ≥10 kbp, scaffolds ≥5 kbp and scaffolds ≥1 kbp in order to test the effects of the N50 statistic and gene recovery rate by removing short and erroneous scaffolds/contigs (electronic supplementary material, figure S1). We determined that the set of scaffolds ≥ 1 kbp did not affect our predicted proteome complement and increased the N50. The filtered ≥1 kbp Platanus assembly, along with the mitochondrial genome assembly, are deposited in EBI with the accessions: Study ID, PRJEB13950; Scaffolds, FLMG01000001-FLMG01004758; and Mitochondria, LT578416. The full assembly and other filtered datasets can be accessed at https://github.com/guyleonard/hyphochytrium or https://www.ebi.ac.uk/biostudies/studies/S-BSST46.

K-mer counting analysis was conducted using Jellyfish along with two publically available scripts (estimate_genome_size.pl and the website GenoScope, see https://github.com/josephryan/estimate_genome_size.pl and [132]). The average sequencing coverage of this assembly was estimated using the ‘estimate_genome_size.pl’ tool for the total assembly and using the ‘genomeCoverageBed’ from BEDTOOLS [133] for the ≥1 kbp subset of scaffolds.

Gene prediction was conducted by using CEGMA to predict which of the 246 core genes are present in our *Hyphochytrium* ≥1 kbp scaffolds; these predicted CEGs are then used in the training step of the program SNAP (see http://korflab.ucdavis.edu/software.html) to generate a set of *ab initio* gene models. The program GeneMark-ES [134] was also run independently on the ≥1 kbp scaffold data, which produced another set of gene models. Both these sets of gene models are in the form of a hidden Markov model (HMM). A first pass of the pipeline MAKER was then run with the default settings, incorporating the gene models from SNAP and GeneMark-ES while also deriving alignment statistics from the 454-transcriptome assembly with tBLASTn, RepeatMasker [135] and exonerate [136]. The output is a set of gene models in GFF3 format. A second round of SNAP was then performed with the new predictions (after the GFF3 has been converted to a HMM) and the program AUGUSTUS [137] is run in *ab initio* mode using the MAKER first pass predictions (i.e. AUGUSTUS default gene models were not used as they are generated from distantly related taxa). Both outputs of SNAP (run 2) and AUGUSTUS are then fed back into MAKER for a second run with stricter settings (gene predictions are available here: https://github.com/guyleonard/hyphochytrium/tree/master/gene_predictions). The final output is a GFF3 file, transcripts and protein FASTA files. The resulting gene predictions were then BLAST searched against the SwissProt database along with InterproScan to assign putative annotations. The results were then used with the program ANNIE [138] to provide the correct format of annotation information to the program GAG [139] for database deposition. The resulting genome data is submitted as an update of a prior BioProject sequence submission [63]; to do this we used the ‘gff3toembl’ program from PROKKA [140].

Previously, we had sequenced a transcriptome from the same culture strain of *Hyphochytrium* [63] using 454 FLX sequencing of cDNA reads and assembled it with Newbler 2.5 [141] using the default cDNA settings. We removed 70 sequences from this assembly of less than 100 bp in length (excluding the polyA regions) and/or contigs that consisted of predominantly repeat motifs. This resulted in 6202 transcript sequences assembled in Newbler 2.5 using the standard settings for cDNA. The reads were also assembled in Trinity but resulted in significantly more (nearly double) contigs.

### 4.3. Assessment of contamination of the genome sequence

To identify any prokaryotic contamination in the ≥1 kbp scaffold assembly, we first conducted BLASTn searches of the assembly using prokaryotic SSU and LSU rDNA sequences as search seeds (*Escherichia coli* taken from [CP012802] and *Sulfolobus acidocaldarius* [NR_043400 & NR_076363]). This analysis only returned sequences of similarity to the *H. catenoides* mitochondria genome assembly, suggesting that no, or very limited, prokaryotic sequence contamination was present. To support this, we subjected all 4758 genome scaffolds to a BLASTx analysis against a database of 65 eukaryotic and 164 representative prokaryotic complete predicted proteomes (electronic supplementary material, table S8) with a gathering threshold of 1 × 10^−10^. This approach did not identify any scaffolds that did not have at least one top hit to a eukaryotic genome for a subsection of the scaffold. Indeed, only 87 of the scaffolds had greater than 50% of the subsections with a top BLAST hit to a prokaryotic genome and only 20 of the scaffolds had greater than 70% of their top BLAST hits to a prokaryotic genome. These 20 scaffolds were inspected manually; 11 of these showed the presence of putative spliceosomal introns and/or other genes more similar to other eukaryotic genes. For the remaining nine scaffolds (totalling 31.8 kbp), we could not exclude them as possible prokaryotic contamination (listed in electronic supplementary material, table S9).

Comparisons of GC content versus read coverage coupled with BLASTn analysis to identify likely aberrant genomic affiliation of assembly scaffolds (e.g. ‘blobology’ [142]) has emerged as useful tool for identifying contamination of genome-sequencing projects [143]. We undertook this approach on both the ≥1 kbp scaffold assembly and the total assembly, and the graphs did not identify any suspect traces of contamination; however, they do show the presence of the mitochondrial genome as an aberrant cluster of ‘blobs’, i.e. with lower than average GC content (electronic supplementary material, figure S13a–d).

A fourth round of checks for contamination were conducted by using tetramer counting of the ≥1 kbp scaffold dataset for the building of Emergent Self Organising Maps [144]. These use similarities in the 4-mer frequencies to build, by way of an artificial neural network, an emergent ‘map’ of the input space properties of the data. Two runs of the software developed by Dick *et al.* [144] were completed (see electronic supplementary material, figure S14a,b): (i) the *Hyphochytrium* scaffolds only and (ii) the *Hyphochytrium* scaffolds along with the scaffolds from eight ‘small’ genomes which were added to the tetramer frequency dataset, (Bacteria (blue): *E. coli*, *Mycobacterium tuberculosis*; Archaea (grey): *Methanococcus vanniellii*, *S. solfataricus*; Fungi (purple): *Encephalitozoon intestinalis, Saccharomyces cerevisiae*; Archaeplastida *Ostreococcus tauri*; Protist (red): *Cryptosporidium hominis*). The maps produced in the electronic supplementary material, figure S14 show no indication of overlap or features indicative of contamination.

### 4.4. *Hyphochytrium catenoides* genome qPCR size estimation

The haploid genome size of *H. catenoides* was estimated using a qPCR-based method [19]; 50 ml of a *H. catenoides* culture, grown in YpSs for 7 days at 25°C, was centrifuged for 3 mins at 3200*g*. The supernatant was removed and genomic DNA was extracted from the remaining cells using a PowerSoil DNA isolation kit (MO BIO Laboratories). An *rps3* PCR standard was amplified using primers Hcat_rps3_F (CGAGGGCTACATGGTCAAGA) and Hcat_rps3_R CCTTTGGCTCGATGATGGTG). Each 25 µl reaction consisted of 0.5 U Phusion polymerase (New England Biolabs), 1× HF buffer, 400 µM dNTPs, 2 µM each primer and 1 µl *H. catenoides* genomic DNA (11.6 ng µl^−1^). Cycling conditions consisted of an initial denaturation of 5 min at 98°C, followed by 30 cycles of 10 s at 98°C, 30 s at 61.0°C and 30 s at 72°C, then a final extension of 5 min at 72°C. The 185 bp PCR product was purified by gel extraction (Thermo Scientific GeneJET Gel Extraction kit) and eluted using elution buffer. Concentration of the purified product was determined using a Qubit dsDNA HS assay kit (Thermo Fisher Scientific). Real-time PCR was used to quantify the number of copies of *rps3* present in each genomic DNA sample. Quantitative PCR was performed in a StepOnePlus real-time PCR system (Thermo Fisher Scientific). Reaction conditions were optimized using a gradient PCR and a standard curve was determined using dilutions of *H. catenoides* genomic DNA and analysed using StepOne software v. 2.3 (slope: −3.367; *y*-intercept: 33.841; efficiency: 98.15%). Each 20 µl PCR contained 10 µl PowerUp SYBR Green Master Mix (Thermo Fisher Scientific), 500 nM each primer (Hcat_rps3_F and Hcat_rps3_R, sequences as above) and 1 µl template DNA. Template was either *H. catenoides* genomic DNA or the PCR standard. Standards were diluted (10^−1^ to 10^−7^) from an initial concentration of 24.7 ng µl^−1^ and run in triplicate, while three independent genomic DNA samples were run in quintuplicate. Cycling conditions were as follows: UDG activation for 2 min at 50°C and DNA polymerase activation for 2 min at 95°C, followed by 40 cycles of 15 s at 95°C and 1 min at 60°C. ROX was used as a reference dye for analysis of CT values. Each reaction was followed by melt-curve analysis, with a temperature gradient of 60–95°C at 0.3°C s^−1^, to ensure presence of only a single amplicon. The PCR standards were used to create a calibration curve (*y* = 8 × 1010 × 10^−0.67*x*^; *R*^2^ = 0.99992); CT values from amplifications of genomic DNA templates were then applied to this curve and the ‘mass’ of the haploid genome was calculated [19]. This value was then used to calculate the haploid genome size, using 615.8771 g mol^−1^ as the mean molar mass of a base pair [145].

### 4.5. Mitochondrial genome assembly

Contigs of putative mitochondrial origin, from both assemblies, were identified by BLAST searches against the mitochondrial genome of *Phytophthora infestans* (NC_002387.1). The contigs from the genome assemblies were visualized, linked and edited using the program SEQUENCHER (https://www.genecodes.com), resulting in two contigs. However, we were unable to circularize the genome using these two fragments. Therefore, regions spanning the gaps in the mtDNA super-contigs were amplified by polymerase chain reaction (PCR) with primers specific to the flanking sequences. Purified PCR products were sequenced using Sanger chemistry (externally at Eurofins Genomics, Ebersberg). This allowed the two contigs to be joined, resulting in a linear genome flanked on one end with *rpl16* and *atp8* on the other. These genes were identical to the other *rpl16* and *atp8* genes found in the assembled mitochondrial genome; we therefore inferred that these represented the beginning and end of a 19 kb inverted repeat (electronic supplementary material, figure S2). Mitochondrial genes were identified and annotated using mfannot (http://megasun.bch.umontreal.ca/cgi-bin/mfannot/mfannotInterface.pl, last accessed 20 June 2017) followed by manual inspection. The putatively circular genome was visualized using CGView [146]. Results and discussion of the mitochondrial data can be found in the electronic supplementary material, figure S2.

### 4.6. Search for *Hyphochytrium catenoides* representatives of key oomycete gene families

Using Pfam searches (Pfam release 29.0) with default defined *e*-value cut-offs, we searched the *H. catenoides* predicted proteome for: NPP1-like proteins (Pfam domain: PF05630), elicitin (PF00964), cutinase (PF01083), pectin methyl esterases (PF01095), pectate lyase (PF03211), polygalacturonase (PF00295) PAN lectin (PF00024), ricin lectin (PF00652), jacalin lectin (PF01419), galactose-binding lectin (PF00337), legume lectin (PF00139), legume-like lectin (PF03388), ABC transporters (PF00005), protein kinase (PF00069 & PF07714), notch protein (PF00066) and haemolysin E (PF06109). In addition, the *H. catenoides* predicted proteome was searched against the MEROPS database (https://merops.sanger.ac.uk/) to identify putative protease inhibitors and proteases and the CAZymes analysis [147] toolkit (using Pfam) at http://mothra.ornl.gov/cgi-bin/cat/cat.cgi?tab=PFAM1 to identify putative carbohydrate interacting proteins. Predicted proteins containing putative RxLR motifs and Crinkler domains were identified using the pipelines described in the literature [148,149].

### 4.7. Secretome analysis

Putatively secreted proteins were predicted using a custom pipeline (https://github.com/fmaguire/predict_secretome/tree/refactor) which identifies sequences predicted to have a signal peptide (via SignalP 4.1 [150]), no TM domains in their mature peptide (via TMHMM 2.0c [151,152]), a signal peptide that targets for secretion (via TargetP [153]) and belonging to the extracellular ‘compartment’ (as predicted by WoLFPSort 0.2 [154]). The CAZY database [155] was downloaded, converted into a BLAST-DB and searched using the predicted proteome and secretomes using BLASTp with an expectation of 1 × 10^−5^. Hit tallies were then summed, proportions calculated and data plotted in Python via the Pandas and Seaborn packages (figure 3).

### 4.8. Phylogenetic analysis of individual gene families

Unless otherwise stated in the figure legends all phylogenetic analyses were conducted using the following protocols. Using BLASTp we used the seed sequence to identify putative homologues across a locally maintained database of eukaryotic and prokaryotic genome-derived protein datasets (electronic supplementary material, table S10) with a gather threshold of 1 × 10^−10^. The Multiple Sequence Comparison by Log-Expectation (MUSCLE) program (v. 3.8.31) [156] was used to produce a multiple sequence alignment for each set of proteins. Alignments were then manually corrected and masked in SeaView (v. 4.2.4) [157]. Sequences that required a high level of site exclusion (due to the sequence not aligning or not masking well) or where they formed long branches in preliminary analysis were removed. The phylogenies were calculated using RAxML [31] with 1000 (non-rapid) bootstrap replicates and using the substitution matrix and gamma distribution identified using ProtTest3 (v. 3.2.1). In some cases, the invariant sites parameter was also included in the model (if indicated in the ProtTest3 analysis).

To identify putative orthologues that arose at the base of the Pseudofungi, gene clusters identified from 74 genomes (electronic supplementary material, table S11) were mapped onto the species phylogeny using a pipeline described at https://github.com/guyleonard/orthomcl_tools and http://dx.doi.org/10.5281/zenodo.51349. Putative pseudofungal specific orthologues were individually tested by conducting gene phylogeny, as described above, combined with additional BLAST searches of NCBI and JGI databases to test and improve taxon sampling (see electronic supplementary material, table S3 for the resulting set of pseudofungal specific orthologues).

### 4.9. Multi-gene concatenated phylogenetic analysis to identify the branching position of *Hyphochytrium catenoides*

Using previously established methods [25,158], we built a concatenated amino acid alignment of 325 orthologues resulting in a masked data matrix of 128 taxa consisting of 90 203 amino acid sites constructed from previously identified seed alignments [25].This dataset encompassed a wide sampling of eukaryotes as well as a broad sampling of stramenopiles available in public databases (e.g. [24,25]). Single gene trees were inferred in RAxML under the PROTCAT + LG model with 100 rapid bootstraps. To examine the effect of the genes used in our phylogenomic analyses we estimated the RTC (i.e. the average of all internode confidence (IC) values for each single gene tree given the bootstrap replicate trees [30,32]). These were calculated in RAxML v. 8.2.6 [31] by comparing the best tree bipartitions to those in the bootstrap trees. The average RTC value for all single gene trees was 0.263. Using the RTC values of all single gene trees, we identified and extracted the top 50% orthologue trees (162 genes, ranging in RTC values from 0.608 to 0.260—named as 162-50RTC dataset). The 162-50RTC genes were concatenated into a supermatrix (128 taxa, 60 059 amino acids) and analysed also in a partitioned and coalescence framework (electronic supplementary material, figure S6b), as with the 325-gene dataset.

Using these alignments (325 gene and 162 gene (162-50RTC) datasets), we calculated a ML with 100 real bootstrap replicates using the IQ-TREE software [27,28] and with the site heterogenous model of evolution LG+*Γ*4+C60+F+PMSF (posterior mean site frequencies) substitution model [29]. The full phylogeny for each are shown in the electronic supplementary material, figure S6a and b. Partitioned phylogenomic species trees were inferred using IQ-TREE v. 1.5.5, allowing each partition to have its own model and evolutionary rates. Each partition was independently analysed under the LG+*Γ*4 model of evolution. This analysis encompassed 1000 ultrafast bootstrap replicates. For summary-coalescent species tree estimation, we employed ASTRAL [23] with default settings and with species tree topology and node support estimated with ASTRAL multilocus bootstrapping (100 replicates). For this coalescence tree, ASTRAL was given all single gene RAxML (PROTCATLGF) best ML phylogenies and 100 rapid bootstrap replicates for each single gene alignment. IC was calculated for the IQ-TREE supermatrix ML tree (LG+*Γ*4+C60+F+PMSF) for both datasets (325 and the 162-50RTC). These were calculated in RAxML v.8.2.6 [31] by comparing the overall ML bipartitions to those in the best individual ML gene trees. These IC along with the TC (Tree certainty) values are mapped on the phylogeny shown in the electronic supplementary material, figure S7a and b.

### 4.10. Identification of genes of plastid ancestry

We constructed a database of taxonomically diverse representative genomes (electronic supplementary material, table S11) and clustered the respective proteomes into putative orthologue groups using OrthoMCL [67], retaining only the groups containing *H. catenoides* genes. Next, we resampled sequences from a wider database of 1205 taxa (electronic supplementary material, table S10) using BLASTp searches [159] to recover up to three sequences from each genome using a gathering threshold of 1 × 10^−10^. We then filtered these clusters, identifying only those containing both a *H. catenoides* gene and genes from photosynthetic or ancestrally-photosynthetic eukaryotic taxa. These sequences were then aligned using MAFFT [160], masked using TRIMAL [110] and a phylogeny was calculated from the data matrix using FASTTREE2 [160]. The resulting phylogenies were manually inspected for a phylogeny that showed *H. catenoides*/pseudofungal/stramenopile genes which: (a) branched within the Archaeplastida radiation, (b) branched with genes of photosynthetic eukaryotes and within a bacterial radiation or (c) branched with cyanobacterial genes. This process required re-running of the phylogenetic pipeline for many gene clusters, either reducing gene sampling or removing long-branch sequences. A subset of 101 gene cluster phylogenies putatively showed a phylogenetic relationship consistent with criteria (a)–(c) described above. The alignments from these clusters were then manually refined, the taxon sampling checked using manual BLAST searches of the NCBI nr database and phylogenies recalculated using the RAxML approach described above. The results of this analysis identified four candidate plastid endosymbiosis acquired genes; these are presented and discussed in the electronic supplementary material, figure S3.

### 4.11. Testing for CYP51 sterol-demethylase drug sensitivity

Azole susceptibility was assessed using a modification of the protocol reported in Warrilow *et al.* [42]. Briefly, fluconazole and clotrimazole were dissolved in dimethyl sulfoxide (DMSO) to a stock concentration of 25.6 mg ml^−1^. Dilutions were then made with DMSO to prepare 100× stock solutions. These stocks were diluted in PYG (1.25 g l^–1^ peptone, 1.25 g l^–1^ yeast extract, 3 g l^–1^ glucose) medium to a final volume of 5 ml, each containing 100 µl of *H. catenoides* liquid culture (grown in YpSs at 25°C shaking for 7 days) to achieve final azole concentrations of 256, 128, 64, 32, 16, 8, 4, 2, 1, 0.5 and 0.25 µg ml^−1^, and with control samples containing 1% (v/v) DMSO. Cultures were incubated, in triplicate, for 7 days at 25°C with 200 r.p.m. shaking, and MIC_100_ was scored manually by assessing for presence/absence of hyphal growth (see electronic supplementary material, figure S8 for the results of the CYP51 and drug treatment analysis).

### 4.12. OmniLog ‘phenotype microarrays'

Measures of 100 ml *H. catenoides* culture were grown in PYG in baffled flasks, at 25°C with 170 r.p.m. shaking to minimize aggregation. Cells were recovered by centrifugation at 3200*g*, washed twice with water and re-suspended in PYG (as above, no carbon-source) to a final concentration of approximately 1.5 × 10^3^ cells ml^−1^. Cells were allowed to recover at 25°C with shaking for 30 min before Dye mix D (Biolog) was added to a 1× final concentration. A measure of 100 µl of cells was inoculated into each well of PM1 and PM2 carbon-source plates and incubated for 7 days at 25°C. Each growth assay was performed in triplicate from independent cultures.

OmniLog Phenotype Microarray outputs were analysed using OPM [162]. Data were aggregated using the ‘opm-fast’ method, analysed using the A parameter (maximum value of OmniLog units reached) and tested by *t*-test. Significant *p*-values were extracted if they resulted in increased respiration rate in comparison with the negative control well A01 (see electronic supplementary material, figure S9 for the results of the OmniLog analysis).

### 4.13. Confirmation of viral genes in the *Hyphochytrium catenoides* assembly and reverse-transcriptase PCR of viral genes

To confirm that the viral genes were assembled correctly and were resident in the *H. catenoides* genome, PCRs across the 3′ and 5′ junctions of the putative viral open reading frame for three of the viral genes *polB*, *MCP* and *mg96* were performed. PCR reactions (25 µl; 1× Phusion HF buffer, 400 µM dNTP mix, 200 nM each primer, 0.5 U Phusion polymerase) were performed with the following cycling conditions: initial denaturation of 5 min at 98°C, followed by 30 cycles of 10 s at 98°C, 30 s at 56–64°C and 1 min at 72°C, then a final extension of 5 min at 72°C. These were purified using a GeneJET PCR Purification Kit or GeneJET Gel Extraction kit (Thermo Scientific) and sequenced to confirm that each product matched the expected amplicon. To confirm that the *mcp* gene was on the same contig as the histone H3 gene, we performed a PCR across these two genes (expected amplicon of 2837 bp) using the same conditions as above, except with an annealing temperature of 64°C and with a 3-min extension. The PCR product was purified and A-tailed using *Taq* polymerase, then cloned using the StrataClone PCR Cloning Kit (Agilent Technologies). The resulting vector was sequenced using T3/T7 primers, with primer-walking to confirm the entire 2.8 kb sequence.

To investigate if the viral derived genes are actively transcribed in our culture conditions, we conducted RT-PCR of the *polB, mcp*, *mg96* and *rps3* virus confirming *polB, mg96 *and* rps3* are expressed in our culture conditions and suggesting that the viral-like genes are transcriptionally active. RNA was extracted from *H. catenoides* using RNA PowerSoil Total RNA Isolation (MoBio). Residual genomic DNA was removed using RQ1 RNase-Free DNase (Promega) and Taq PCR was performed to confirm absence of DNA. Reverse-transcriptase PCR (RT-PCR) was then performed using a Qiagen OneStep kit according to the manufacturer's instructions, alongside genomic DNA positive and no-template controls. The following cycling conditions were used: reverse transcriptase of 30 min at 50°C and initial denaturation of 15 min at 94°C, followed by 32 cycles of 1 min at 94°C, 1 min at 50°C and 1 min at 72°C, then a final extension of 10 min at 72°C. Samples were then analysed on a 2% (w/v) agarose gel.

### 4.14. WGA staining

*Hyphochytrium catenoides* was grown for 7 days at 25°C and 100 µl of mycelial growth was removed and suspended in 1 ml PBS, then 5 µg ml^−1^ calcofluor white (Fluka) and 10 µg ml^−1^ WGA, Alexa Fluor 488 conjugate (Invitrogen) were added and cells were incubated for 30 min in the dark. Cells were washed twice in PBS and imaged using an Olympus IX73 microscope on a 40× objective. Unstained cells were also checked to confirm the absence of autofluorescence.

## Supplementary Material

Supplementary Figure Legends

## Supplementary Material

Figure S1

## Supplementary Material

Figure S2

## Supplementary Material

Figure S3

## Supplementary Material

Figure S4

## Supplementary Material

Figure S5

## Supplementary Material

Figure S6

## Supplementary Material

Figure S7

## Supplementary Material

Figure S8

## Supplementary Material

Figure S9

## Supplementary Material

Figure S10

## Supplementary Material

Figure S11

## Supplementary Material

Figure S12

## Supplementary Material

Figure S13

## Supplementary Material

Figure S14

## Supplementary Material

Figure S15

## Supplementary Material

Table S1

## Supplementary Material

Table S2

## Supplementary Material

Table. S3

## Supplementary Material

Table S4

## Supplementary Material

Table S5

## Supplementary Material

Table S6

## Supplementary Material

Table S7

## Supplementary Material

Table S8

## Supplementary Material

Table S9

## Supplementary Material

Table S10

## Supplementary Material

Table S11

## Supplementary Material

Table S12

## Data accessibility

All relevant data generated for this project can be found at https://www.ebi.ac.uk/biostudies/studies/S-BSST46 or https://github.com/guyleonard/hyphochytrium. Sequencing data can be accessed via the EMBL accessions SRX033129 (Illumina GAiix) and ERS1151585 (Illumina HiSeq). Genomic assemblies of the nuclear and mitochondrial genomes are available under the EMBL accessions: study ID: PRJEB13950, scaffolds: FLMG01000001-FLMG01004758 and Mitochondria: LT578416.
