# Supplementary material for: Comparative genomic analysis of the ‘pseudofungus’ Hyphochytrium catenoides
Source: Open Biol. 2018 Jan 10;8(1):170184. doi: 10.1098/rsob.170184 (PMC5795050; doi:10.1098/rsob.170184)
Supplement: Supplementary Figure Legends [file rsob170184supp1.docx]

**Supplementary Figure Legends**

**Fig. S1. Comparison of transcriptome to genome assembly and analysis of genome completeness.** Using a range of methods, we assembled and tested the completeness of the *H. catenoides* genome (see methods). Comparisons measuring the fraction of transcriptome data that aligned to the genome with BLAST, along with CEGMA and BUSCO demonstrated that the genome assembly was predicted to be respectively; 97.8%, 91.5%, and 52% complete in terms of gene sampling. We suspect that both CEGMA and BUSCO (v1.2) underestimate the completeness of genomes as the core gene list is derived from a subset of eukaryotic genomes that does not fully sample a diverse collection of eukaryotic genomes, (e.g. BUSCO version 1.2 only samples fungi and metazoan genomes). Fig. 1A+B shows the CEGMA/BUSCO scores across the stramenopiles, demonstrating a 27/16 complete/partial gene family absence in the *H. catenoides* assembly according to CEGMA. This is a moderate level of absence compared with other stramenopile genomes (e.g. *Blastocystis hominis* and *Ectocarpus siliculosus* with 76/53 and 71/31 complete/partial gene family absences respectively). Using the CEGMA and transcriptome comparison approaches described, we then investigated the completeness of the assembly when scaffolds below 1 kbp, 5 kbp, and 10 kbp were serially removed. For each dataset, the N50 statistic was computed, along with gene predictions, CEGMA results and ‘BLAST’ hits against the transcriptome. This analysis demonstrates that the removal of scaffolds less than 1 kbp has a negligible effect on the predicted completeness of the genome.

**Fig. S2. The mitochondrial genome of *H. catenoides*.** Using common mitochondrial marker genes as query sequences for tBLASTx searches we searched the *H. catenoides* for fragments of the mitochondrial genome. These analyses showed that the organelle genome assembly was highly fragmented. By searching both the Platanus and an alternative Ray assembly [1] we identified and re-assembled, two mitochondrial genome contigs using Sequencher [2]. We then used multiple primers for targeted PCR amplification and joined the two fragments of the mitochondrial genome, resulting in a linear fragment of 48,124 bp in size (inner red semi-circle). Based on physical genome mapping [3, 4], the *H. catenoides* mitochondrial genome is reported to be circular and 54 kb with a ~14 kb inverted repeat (a feature present in some oomycetes). We used multiple combinations of PCR primers to try and join the other sides into a circular genome, but could not amplify a bridging region suggesting that our assembly is missing a large section of the mitochondrial genome, likely the inverted repeat. Based on the sequence similarity at the ends of our mitochondrial assembly to sequences nested within the major contig (see methods), we inferred a 19 kb inverted repeat which putatively completes the circular genome (inner green semi-circle).

Annotation showed that the *H. catenoides* mitochondrial genome encodes 33 mitochondrial protein-coding genes (plus six putative ORFs with no similarity to other mitochondrial genes), 21 unique tRNAs for 18 amino acids, and two ribosomal RNA genes. Except for the lack of rps7 and TatC genes, this gene set is very similar to reported oomycete mitochondrial genomes [4]. No introns were predicted.

The assembly demonstrated the presence of genes similar to mitochondrial *atp1* in both the mitochondrion and also the putative nuclear genome assembly (Hypho2016_00005578). The *atp1* gene has been transferred from the mitochondrial genome to the nuclear genome independently in several lineages but is retained in the mitochondrial genomes of a range of stramenopiles including the eustigmatophytes (e.g. *Nannochloropsis*), oomycetes and *Cafeteria* [4, 5]. We reconstructed the phylogeny of the *atp1* genes to test the hypothesis that the nuclear *atp1* gene in *H. catenoides* may be the result of an endosymbiont gene transfer. We show that while the mitochondria-encoded *atp1* branches within a clade including all other bona-fide mitochondrial *atp1* genes (regardless of which genome- nuclear or mitochondrial -they are encoded), surprisingly the *H. catenoides* nuclear-encoded *atp1*-like gene branched with oomycete and Nannochloropsis sequences as a separate paralogue of uncertain ancestry, sister to all bacterial-derived (i.e. bacterial, mitochondrial, and chloroplast) *atp1* genes (Fig. S15). With the exception of *Nannochloropsis*, these divergent *atp1*-like proteins have putative N-terminal mitochondrial targeting peptides, indicating that these proteins may represent a stramenopile-specific nuclear encoded mitochondrial *atp1*-like gene.

The figure is illustrated in the following manner. Ribosomal genes (pink), tRNA genes (red) – also labelled as single letters, and protein coding genes (blue) are represented around the mitochondrial genome (black). Genes represented on the outside are encoded clockwise whereas genes represented on the inside are encoded counter-clockwise. Small and large ribosomal protein genes have been abbreviated as ‘s’ and ‘L’, respectively. The inferred inverted repeat regions are overlaid with green arrows. The inner circle depicts the original 48,124 bp assembly (red), the inferred ~19 kb inverted repeat (green), and the overlap (grey).

**Fig. S3. Investigation of plastid genome and nuclear encoded genes of plastid derived endosymbiotic ancestry.** Many stramenopiles are photosynthetic and retain a plastid of secondary endosymbiotic ancestry [6, 7]. Some commentators have hypothesised that this plastid is derived from an ancient event and as such the stramenopile plastids has the same derivation as the plastid organelles present in dinoflagellates and Apicomplexa (see [8]), which would mean that the Pseudofungi were ancestrally photosynthetic but have lost both the plastid genome and organelle. This hypothesis has been challenged on a number of levels; either by phylogenomic data which supports a pattern of multiple (or serial) eukaryote–eukaryote endosymbioses [9-12], or alternatively evidence suggesting the oomycetes lack a statistically significant footprint of genes of plastid endosymbiotic ancestry [13]. As such, the ancestry of secondary ‘plastid’ endosymbiosis/es relative to the radiation of the stramenopiles remains controversial. Similar to oomycetes, hyphochytrids seem to lack a plastid organelle and photosynthetic function.

To search for possible plastid genomes, we took the proteome encoded by the plastid of *Chlamydomonas reinhardtii* (69 proteins) and *Thalassiosira pseudonana* (141 proteins) as query sequences for a tBLASTn search of the entire genome assembly (including contigs and scaffolds below 1 kbp in size). Only a minority of searches recovered any hits in *H. catenoides* and these were shown by reciprocal BLAST searches to be putative mitochondrial-located ribosomal genes, suggesting that there is no plastid genome present in our genome assembly.

The established approach for identifying endosymbiotic ancestry of protist lineages that appear to have no endosymbiotic organelles is to identify genes of endosymbiotic ancestry present on the nuclear genome. This approach has worked well for identifying cryptic endosymbiotic ancestry for both the plastid [14, 15] and mitochondrial [16-18] organelles. Genes derived from the endosymbiosis that gave rise to the primary plastid organelle should branch with or within a cluster of cyanobacterial genes on a phylogenetic tree. Using a phylogenomic pipeline described in the methods, we constructed a phylogeny for all gene-clusters which included both a *H. catenoides* gene and a gene of a eukaryote with a plastid or of photosynthetic ancestry, and included a wide sampling of putative homologues from both eukaryotic and prokaryotic taxa. This process resulted in 8970 preliminary phylogenetic trees, which we searched for a phylogenetic relationship that demonstrated *H. catenoides* genes branching with photosynthetic eukaryotes or eukaryotes with photosynthetic ancestry, which in turn branched within a cluster of bacterial sequences. Following the example of others [19, 20] we reasoned that cyanobacterial signature was likely mixed up by horizontal gene transfer within the prokaryotes and so any *H. catenoides* gene cluster that showed the relationship described above (i.e. [[*H. catenoides* + Plastid-bearing-Eukaryotes]bacteria]) potentially represented a relict gene ancestrally derived from the endosymbiosis that gave rise to the plastid. For 101 preliminary phylogenies that showed this relationship, the amino acid sequence alignment was edited, masked manually and the taxon sampling checked using additional BLAST searches of NCBI nr protein database. This process identified four gene families where *H. catenoides* protein sequences (in three cases branching with other Pseudofungi/Stramenopiles taxa) branched with photosynthetic eukaryotic taxa sister to bacterial taxa. In all four cases (Fig. S3A-D) we did not detect a putative orthologue of these gene families in eukaryotic taxa thought not to have had a plastid endosymbiotic ancestry (i.e. Fungi, Metazoa and Amoebozoa). In only one case did the eukaryotic sequences branch with the cyanobacteria, although the bootstrap support for this relationship was weak (Fig. S3D).

We then used TargetP [21] searches to investigate if the putative orthologues of the *H. catenoides* protein sequence encoded by Archaeplastida taxa had putative plastid targeting N-terminal peptide, demonstrating that none of these protein sequences contained such peptide characteristics.

Taken together these data suggest that the putative proteome *of H. catenoides* contains a subset of four genes that could be putatively of plastid endosymbiotic ancestry. It is possible that our survey protocol missed a number of genes of endosymbiotic ancestry. This is because pipeline generated trees often have inappropriate taxon sampling (i.e. too large), with the pipeline-generated alignments too noisy to allow effective phylogenetic analysis. As such, trees generated often show no meaningful branching relationships or lack resolution. To partially control for this problem, we reran many of our candidate genes, where the resulting alignment was very large, using a subset of selected genomes based on the initial tree.

It is difficult to conclude if four gene families identified here, which in all cases do not show a strongly supported cyanobacterial/plastid ancestry for the eukaryotic genes, represents a significant result which confirms plastid ancestry for the Pseudofungi. Indeed, others have argued that similar numbers of gene phylogenies indicate a plastid endosymbiotic ancestry for different protist groups that have no evidence of a plastid organelle [14, 15]. Conversely, it is difficult to prove a negative, because it is certainly possible that the majority of plastid/cyanobacterial derived genes have been lost, while the complement of plastid endosymbiosis derived genes retained may be so small in number and with hugely complicated phylogenetic histories, that it would be near impossible to recover using pipeline based tree building methods. As such these results are inconclusive with regards to photosynthetic/plastid ancestry of *H. catenoides*.

All phylogenies were calculated using Maximum Likelihood methods with 1000 bootstrap replicates as described in the methods. Sequences from all taxa possessing plastid of primary endosymbiotic ancestry (i.e. Archaeplastida) were searched using TargetP for evidence of an N-terminal targeting peptide. Alignments are provided at [https://github.com/guyleonard/hyphochytrium/blob/master/manuscript/data/](https://github.com/guyleonard/hyphochytrium/blob/master/manuscript/data/data_s1_fig_1a_hyphochytrium_phylogeny.tree). **(A)** Pas -Histidine Kinase domain containing protein phylogeny calculated from a data matrix of 46 sequences and 281 alignment positions. **(B)** Cytidylytransferase family domain containing protein phylogeny calculated from a matrix of 38 sequences and 178 alignment positions. **(C)** Phylogeny of a conserved hypothetical protein calculated from a data matrix of 41 sequences and 143 alignment positions. **(D)** Phylogeny of a putative Heme oxygenase domain calculated from a data matrix of 49 sequences and 186 alignment positions.

**Fig. S4. Identification of SNP frequency mapped to individual scaffolds.** A plot of SNP frequency for each scaffold of the draft genome arranged by length. SNPs are more common in shorter scaffolds. Blue line indicates trend-line with standard deviation. Two aberrant scaffolds with low SNP frequency are identified by a red circle.

**Fig. S5. K-mer coverage of genome assembly showing two peaks indicative of a diploid genome.** A GenomeScope graph of the k-mer profile generated by using Jellyfish displaying k-mer coverage vs frequency. The two k-mer peaks are indicative of a diploid genome.

**Fig S6 A eukaryote-wide phylogeny. (A)**. 325 gene (90,230 amino acid) phylogeny of eukaryotes. The maximum likelihood tree was built using a supermatrix approach in IQ-Tree under the site heterogenous model of evolution, LG+G4+FMIX (empirical, C60)+PMSF. Values at nodes are ML bootstrap (MLBS) (100 real BS replicates), IQ-TREE LG+G4+FMIX (empirical, C60)+PMSF, MLBS under a the partitioned dataset using the LG+G4 model of evolution per partition (1000 ultrafast BS replicates), 100 ASTRAL coalescence multilocus bootstrap replicates, respectively. Bootstrap values below 50% are denoted as a *. Circles denote 99% or above values from all analyses. **(B)**. Dataset is based on the 162 gene dataset in which the top 50% relative tree confidence scores of genes in Figure S6A were collected (60,059 amino acids). The maximum likelihood tree was built using a supermatrix approach in IQ-Tree under the site heterogenous model of evolution, LG+G4+FMIX (empirical, C60)+PMSF. Values at nodes are ML bootstrap (MLBS) (100 real BS replicates), IQ-TREE LG+G4+FMIX (empirical, C60)+PMSF, MLBS under a the partitioned dataset using the LG+G4 model of evolution per partition (1000 ultrafast BS replicates), 100 ASTRAL coalescence multilocus bootstrap replicates, respectively. Bootstrap values below 50% are denoted as a *. Circles denote 99% or above values from all analyses.

**Fig. S7. Internode- and tree-consistency (A).** Probabilistic internode confidence values mapped onto the tree of Figure S6A. These were calculated in RAxML v 8.2.6 by comparing the overall ML bipartitions to those in the best individual ML single gene trees. Values shown are internode consistency (IC) and tree consistency (TC). **(B)**.Probabilistic internode confidence values mapped onto the tree of Figure S6B. These were calculated in RAxML v 8.2.6 by comparing the overall ML bipartitions to those in the best individual ML single gene trees. Values shown are internode consistency (IC) and tree consistency (TC).

**Fig. S8. Phylogeny indicating the branching position of the *Hyphochytrium* putative CYP51 sterol-demethylase protein and results of drug sensitivity assay.** Concentrations were as used in [23], while the MIC showed considerably increased potency as reported for *S. parasitica.*

**Fig. S9. OmniLog Phenotype Microarray (PM) respiration curves.** Triplicate cultures of *H. catenoides* were suspended in PY-G medium and inoculated into OmniLog PM1 (A) and PM2 (B) plates. PM plates were incubated at 25°C for 7 days, and outputs were analysed using the R package opm, with the A-parameter (maximum value of OmniLog units reached) calculated to investigate differing utilization of carbon sources. Each replicate is indicated by an independent respiration curve for each well, wells with a significantly altered respiration/growth rate are highlighted in green.

**Fig. S10 Photoreceptors putatively encoded on the *Hyphochytrium* genome. (A)** Distribution of known photoreceptor families across a subset of stramenopile genomes. **(B)** Subsection of the Rhodopsin alignment showing the conserved location of the Schiff base where the retinal is covalently linked. **(C)** Phylogeny of the rhodopsin gene family showing phylogenetic position of the *Hyphochytrium* putative rhodopsins.

**Fig. S11. Histone H3 phylogeny.** ML phylogeny of the putative Histone H3 encoding gene found next to viral-like *H. catenoides* *mcp* gene in our genome assembly. Here we have used a DNA alignment for the purpose of calculating a phylogeny as this gene family is highly conserved at the amino acid level. The tree is illustrated to identify major taxonomic groups: purple for alveolates and blue for stramenopiles. The H3 histone gene from *Hyphochytrium* scaffolds are illustrated in red with the scaffold ID and coordinates on the scaffold. A red diamond indicates the *Hyphochytrium* scaffold harbouring a viral-like MCP gene, all other accession numbers are RefSeq accession numbers. This gene family includes a large number of putative homologs, as such we have reduced redundancy by clustering similar sequences using 95% global identity. Alignment was made using MAFFT [24] with alignment sampling conducted using trimAL [25] resulting in a data matrix of 411 nucleotide sites and 164 sequences. The phylogeny was calculated using IQTREE v1.5 with a GTR + I + Γ4 substitution model and 100 non-parametric bootstrap replicates.

**Fig. S12. Representative *Hyphochytrium* TEM images showing absence of evidence for viral particles or a viral factory.** In preparation for microscopy *H. catenoides* cultures were grown in PYG medium at 25°C, 170 rpm. Samples were then subject to high pressure freezing using a Leica EM RTS and were left to undergo free substitution using an infiltration mixture of 0.1% uranyl acetate and 1% osmium tetroxide as an overnight fixative. The fixative was removed and the concentration of EPON resin was increased in over six increments until embedding occurred at 100% EPON. The resultant palate was cut using a diamond knife and exposed to lead citrate before being mounted on a standard copper grid. All images were obtained using a JEOL JEM 1400 transmission electron microscope. Twenty cells on one EM slide were subject to cursory inspection for evidence of viral-like structures. This figure shows six imaged cells (A, C, G, J, K & L, with additional micrographs of sub-cellular regions: B, D, E, F, H, I).

**Fig. S13. ‘Blobplot’ analysis of genome assembly. ‘**Blobplots’ allow the visualization of genome assemblies by displaying putative taxon-annotated GC-coverage plots. **(A/B)** Plots showing all scaffolds from the draft assembly with BLAST annotation from the NCBI ‘nt’ database. **(B)** the transcriptome of *Hyphochytrium* and the mitochondrial genome assembly were included in the reference database. Notice the small circle of *Hyphochytrium* annotated blobs in the upper-left of the image, these aberrant GC contigs are mitochondrial hits. **(C/D)** Shows the same images, however, only scaffolds greater than 1kbp have been included in the analysis.

**Fig. S14. Emergent properties graphs for assessment of contamination in the *Hyphochytrium* genome assembly.** Two emergent self-organizing maps show the scaffold data plotted as tetra-mer frequencies represented by a neural network mapping approach. **(A)** To determine contamination, the map was run on the *Hyphochytrium* scaffolds and does not show extensive regions of variance. **(B)** To further investigate the contamination in our genome, other eukaryotic genomes and prokaryotes were ‘spiked’ into the process, here we see clear separation of all the genomes. *Hyphochytrium scaffolds* (orange), Bacteria (blue): *E. coli*, *Mycobacterium tuberculosis*; Archaea (grey): *Methanococcus vanniellii, Sulfolobus solfataricus*; Microsporidia/Fungi (purple): *Encephalitozoon intestinalis, S. cerevisiae*; Archaeplastida (green): *Ostreococcus tauri*; Protist (red): *Cryptosporidium hominis*.

**Fig. S15. ATP1 gene phylogeny showing the presence of a divergent nuclear and mitochondria encoded proteins in the stramenopiles.** The nuclear encoded *H. catenoides* Atp1-like protein sequence was used as a query in BLAST and pHMMer searches. Sequences retrieved were aligned with sequences from [5] using MUSCLE. Sequences were subjected to phylogenetic analysis using RaxML with 500 bootstraps using the LG substitution matrix. Atp1-like sequences predicted to be targeted to mitochondria by TargetPv1.1 are indicated by an encircled "M".

1 Boisvert, S., Raymond, F., Godzaridis, E., Laviolette, F., Corbeil, J. 2012 Ray Meta: scalable de novo metagenome assembly and profiling. *Genome Biol*. **13**, R122. (gb-2012-13-12-r122 [pii] 10.1186/gb-2012-13-12-r122)

2 GCC. Sequencher. 4.2 ed. Ann Arbor: Gene Codes Corporation 2003.

3 McNabb, S. A., Eros, R. W., Klassen, G. R. 1988 Presence and absence of large inverted repeats in the mitochondrial DNA of Hyphochytriomycetes. *Can J Bot*. **66**, 2377-2379. (10.1139/b88-322)

4 O'Brien, M. A., Misner, I., Lane, C. E. 2014 Mitochondrial genome sequences and comparative genomics of *Achlya hypogyna* and *Thraustotheca clavata*. *J Eukaryot Microbiol*. **61**, 146-154. (10.1111/jeu.12092)

5 Ševčíková, T., Klimeš, V., Zbránková, V., Strnad, H., Hroudová, M., Vlček, Č., Eliáš, M. 2016 A comparative analysis of mitochondrial genomes in eustigmatophyte algae. *Genome Biol. Evol.* **8**, 705-722. (10.1093/gbe/evw027)

6 Archibald, J. M. 2009 The puzzle of plastid evolution. *Curr. Biol.* **19**, R81-88. (S0960-9822(08)01485-1 [pii] 10.1016/j.cub.2008.11.067)

7 Derelle, R., López-García, P., Timpano, H., Moreira, D. 2016 A phylogenomic framework to study the diversity and evolution of stramenopiles (=heterokonts). *Mol. Biol. Evol.* **33**, 2890-2898. (10.1093/molbev/msw168)

8 Cavalier-Smith, T. 2000 Membrane heredity and early chloroplast evolution. *Trends Plant Sci.* **5**, 174-182.

9 Baurain, D., Brinkmann, H., Petersen, J., Rodríguez-Ezpeleta, N., Stechmann, A., Demoulin, V., Roger, A. J., Burger, G., Lang, B. F., Philippe, H. 2010 Phylogenomic evidence for separate acquisition of plastids in cryptophytes, haptophytes, and stramenopiles. *Mol. Biol. Evol.* **27**, 1698-1709. (10.1093/molbev/msq059)

10 Stiller, J. W., Schreiber, J., Yue, J., Guo, H., Ding, Q., Huang, J. 2014 The evolution of photosynthesis in chromist algae through serial endosymbioses. *Nat Commun*. **5**, 5764. (10.1038/ncomms6764)

11 Petersen, J., Ludewig, A.-K., Michael, V., Bunk, B., Jarek, M., Baurain, D., Brinkmann, H. 2014 *Chromera velia*, endosymbioses and the Rhodoplex hypothesis—plastid evolution in cryptophytes, alveolates, stramenopiles, and haptophytes (CASH Lineages). *Genome Biol. Evol.* **6**, 666-684. (10.1093/gbe/evu043)

12 Dorrell, R. G., Gile, G., McCallum, G., Méheust, R., Bapteste, E. P., Klinger, C. M., Brillet-Guéguen, L., Freeman, K. D., Richter, D. J., Bowler, C. 2017 Chimeric origins of ochrophytes and haptophytes revealed through an ancient plastid proteome. *Elife*. **6**, e23717. (10.7554/eLife.23717)

13 Stiller, J. W., Huang, J., Ding, Q., Tian, J., Goodwillie, C. 2009 Are algal genes in nonphotosynthetic protists evidence of historical plastid endosymbioses? *BMC Genomics*. **10**, 484. (10.1186/1471-2164-10-484)

14 Reyes-Prieto, A., Moustafa, A., Bhattacharya, D. 2008 Multiple genes of apparent algal origin suggest ciliates may once have been photosynthetic. *Curr Biol*. **18**, 956-962. (<http://dx.doi.org/10.1016/j.cub.2008.05.042>)

15 Huang, J., Mullapudi, N., Lancto, C. A., Scott, M., Abrahamsen, M. S., Kissinger, J. C. 2004 Phylogenomic evidence supports past endosymbiosis, intracellular and horizontal gene transfer in *Cryptosporidium parvum*. *Genome Biol.* **5**, R88.

16 Riordan, C. E., Ault, J. G., Langreth, S. G., Keithly, J. S. 2003 *Cryptosporidium parvum Cpn60* targets a relict organelle. *Curr. Genet.* **44**, 138-147.

17 Roger, A. J., Svard, S. G., Tovar, J., Clark, C. G., Smith, M. W., Gillin, F. D., Sogin, M. L. 1998 A mitochondrial-like chaperonin 60 gene in *Giardia lamblia*: evidence that diplomonads once harbored an endosymbiont related to the progenitor of mitochondria. *Proc. Natl. Acad. Sci. USA*. **95**, 229-234.

18 Hirt, R. P., Healy, B., Vossbrinck, C. R., Canning, E. U., Embley, T. M. 1997 A mitochondrial HSP70 orthologue in *Vairimorpha necatrix*: molecular evidence that microsporidia once contained mitochondria. *Curr. Biol.* **7**, 995-998.

19 Martin, W., Herrmann, R. G. 1998 Gene transfer from organelles to the nucleus: how much, what happens, and Why? *Plant Physiol.* **118**, 9-17.

20 Martin, W., Rujan, T., Richly, E., Hansen, A., Cornelsen, S., Lins, T., Leister, D., Stoebe, B., Hasegawa, M., Penny, D. 2002 Evolutionary analysis of *Arabidopsis*, cyanobacterial, and chloroplast genomes reveals plastid phylogeny and thousands of cyanobacterial genes in the nucleus. *Proc. Natl. Acad. Sci. USA*. **99**, 12246-12251.

21 Emanuelsson, O., Nielsen, H., Brunak, S., von Heijne, G. 2000 Predicting subcellular localization of proteins based on their N-terminal amino acid sequence. *J. Mol. Biol.* **300**, 1005-1016.

22 Noguchi, F., Tanifuji, G., Brown, M. W., Fujikura, K., Takishita, K. 2016 Complex evolution of two types of cardiolipin synthase in the eukaryotic lineage stramenopiles. *Mol Phylogenet Evol*. **101**, 133-141. (<http://dx.doi.org/10.1016/j.ympev.2016.05.011>)

23 Warrilow, A. G. S., Hull, C. M., Rolley, N. J., Parker, J. E., Nes, W. D., Smith, S. N., Kelly, D. E., Kelly, S. L. 2014 Clotrimazole as a potent agent for treating the oomycete fish pathogen *Saprolegnia parasitica* through inhibition of sterol 14α-demethylase (CYP51). *Appl Environ Microbiol*. **80**, 6154-6166. (10.1128/AEM.01195-14)

24 Katoh, K., Kuma, K., Toh, H., Miyata, T. 2005 MAFFT version 5: improvement in accuracy of multiple sequence alignment. *Nucleic Acids Res*. **33**, 511-518. (33/2/511 [pii] 10.1093/nar/gki198)

25 Capella-Gutierrez, S., Silla-Martinez, J. M., Gabaldon, T. 2009 trimAl: a tool for automated alignment trimming in large-scale phylogenetic analyses. *Bioinformatics*. **25**, 1972-1973. (btp348 [pii] 10.1093/bioinformatics/btp348)
