# Supplementary figures and images for: Comparative genomic analysis of the ‘pseudofungus’ Hyphochytrium catenoides
Source: Open Biol. 2018 Jan 10;8(1):170184. doi: 10.1098/rsob.170184 (PMC5795050; doi:10.1098/rsob.170184)

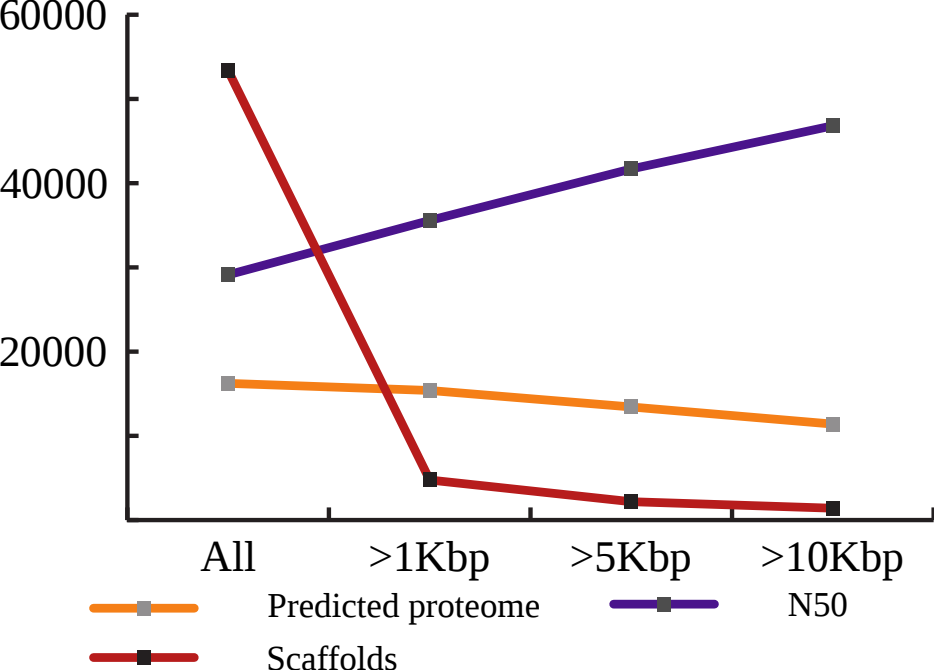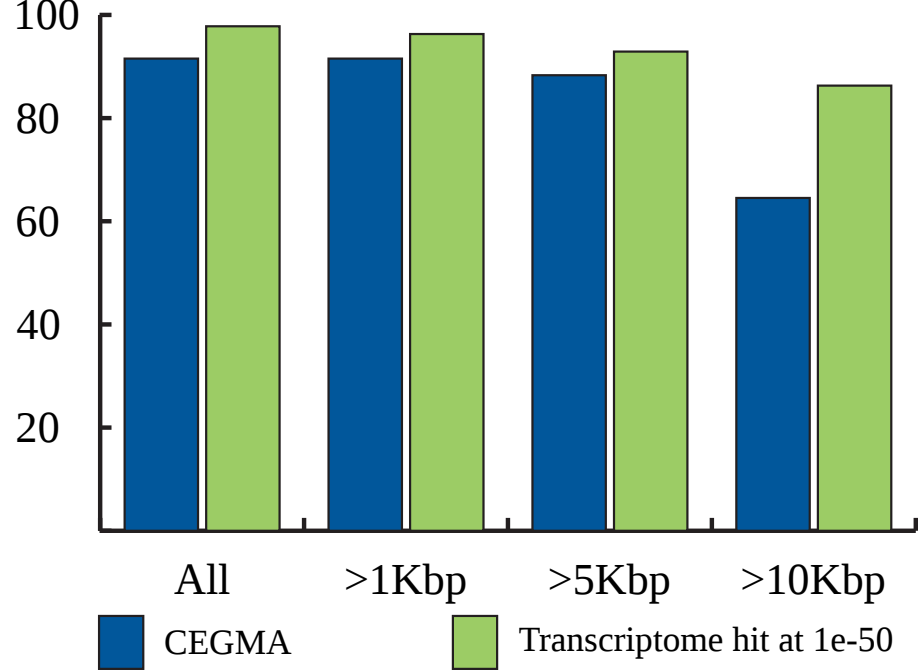

Supplement: Figure S1 [file rsob170184supp2.pdf]

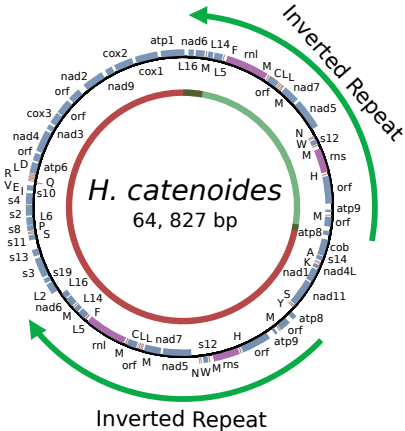

Supplement: Figure S2 [file rsob170184supp3.pdf]

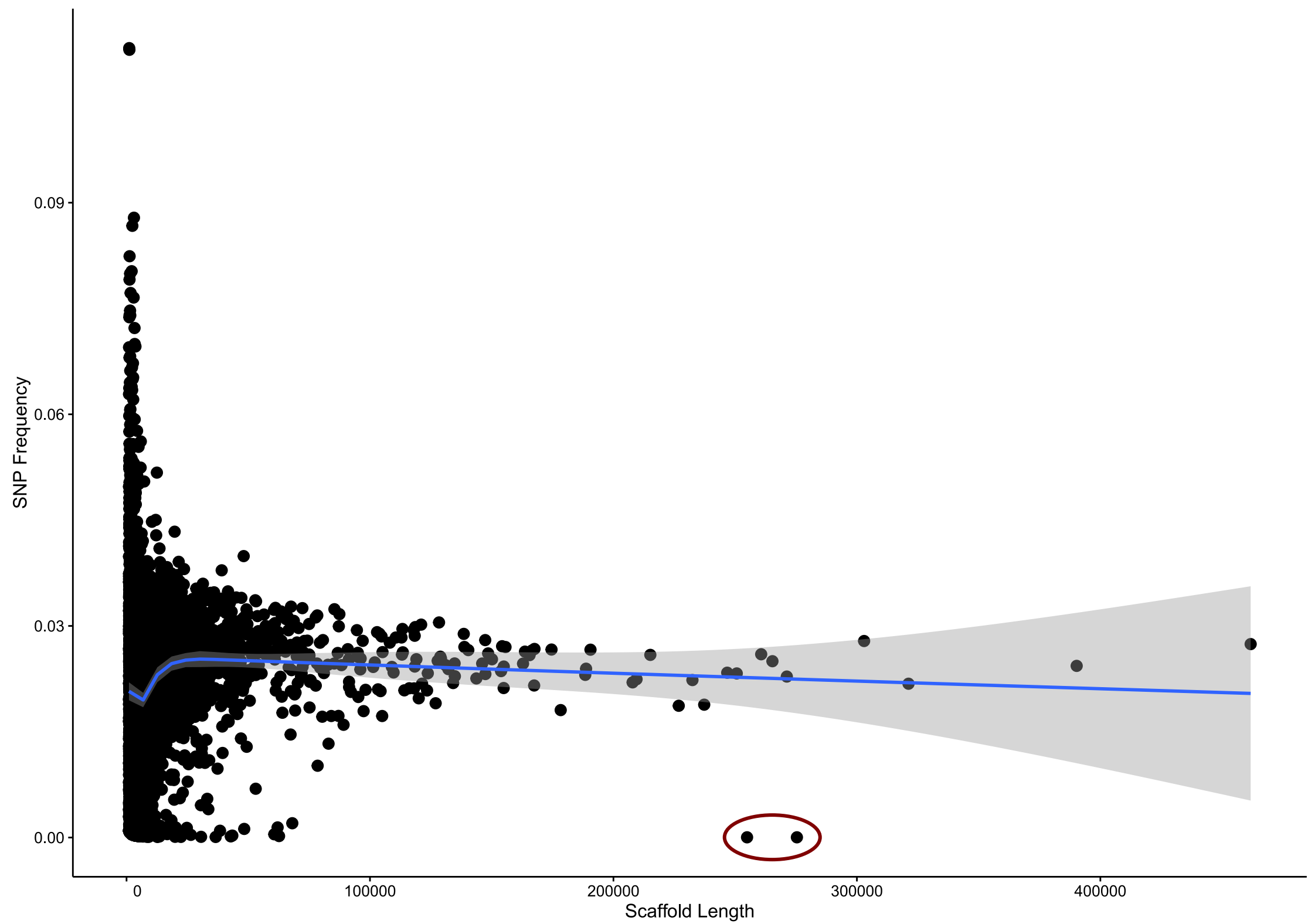

Supplement: Figure S4 [file rsob170184supp5.pdf]

A

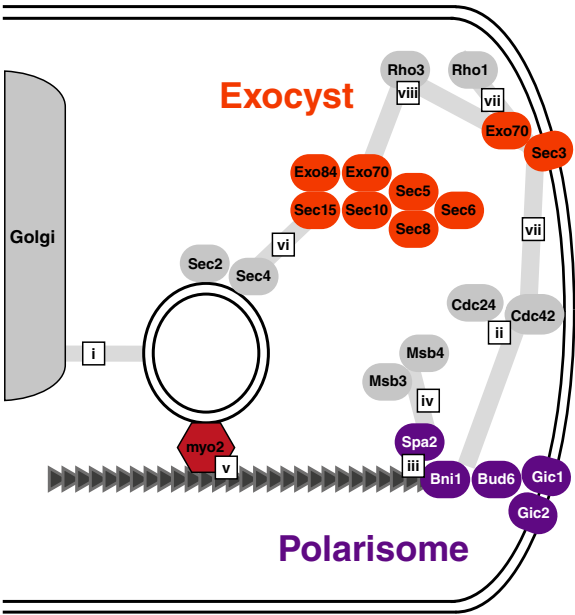

B

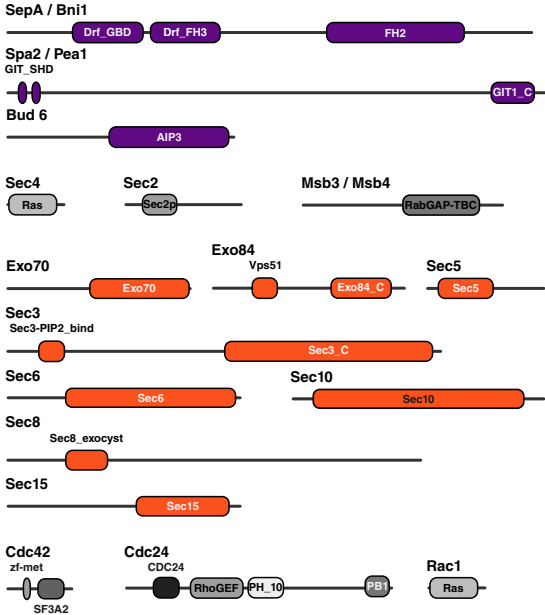

C

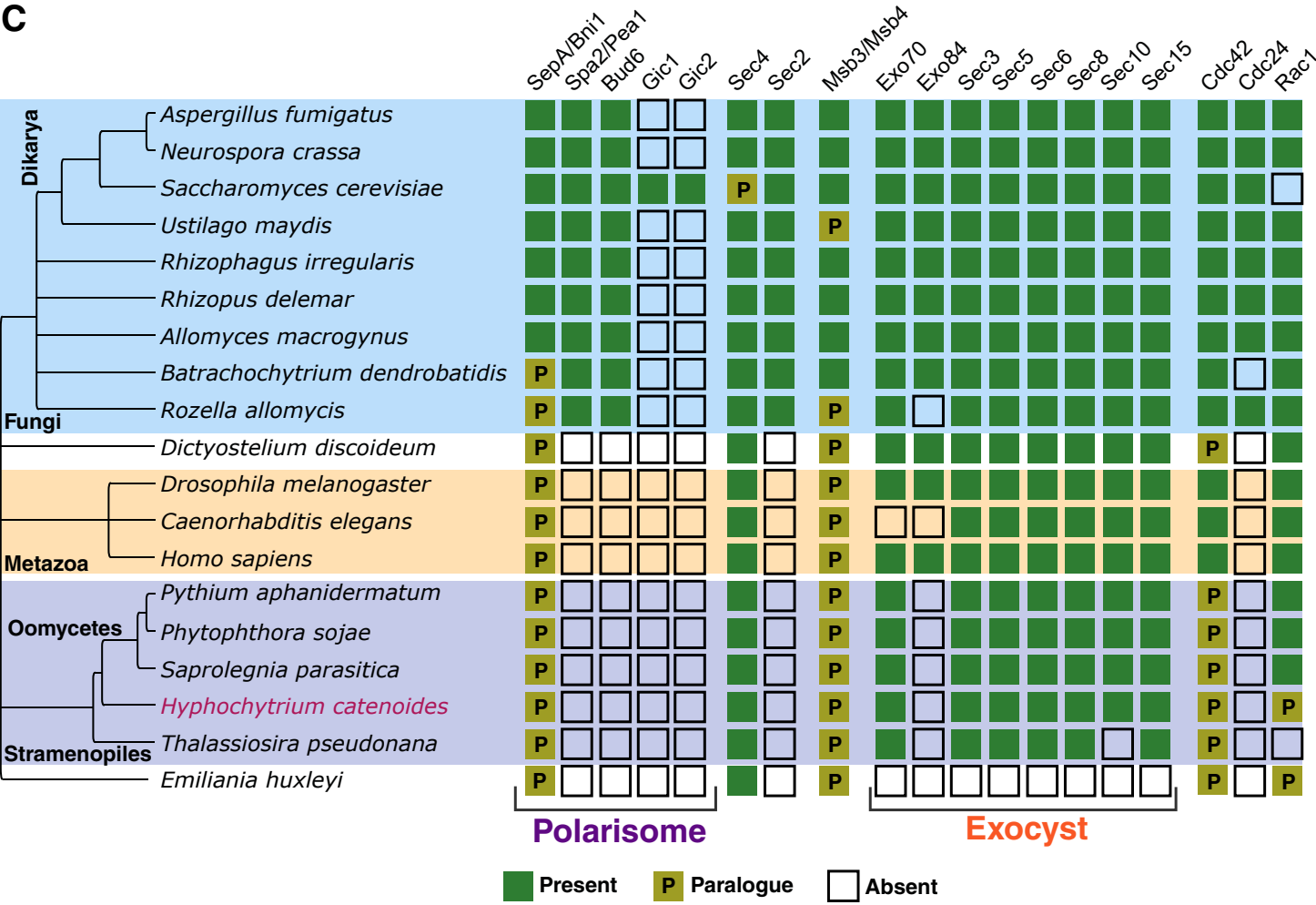

Supplement: Figure S5 [file rsob170184supp6.pdf]

Fig 6A

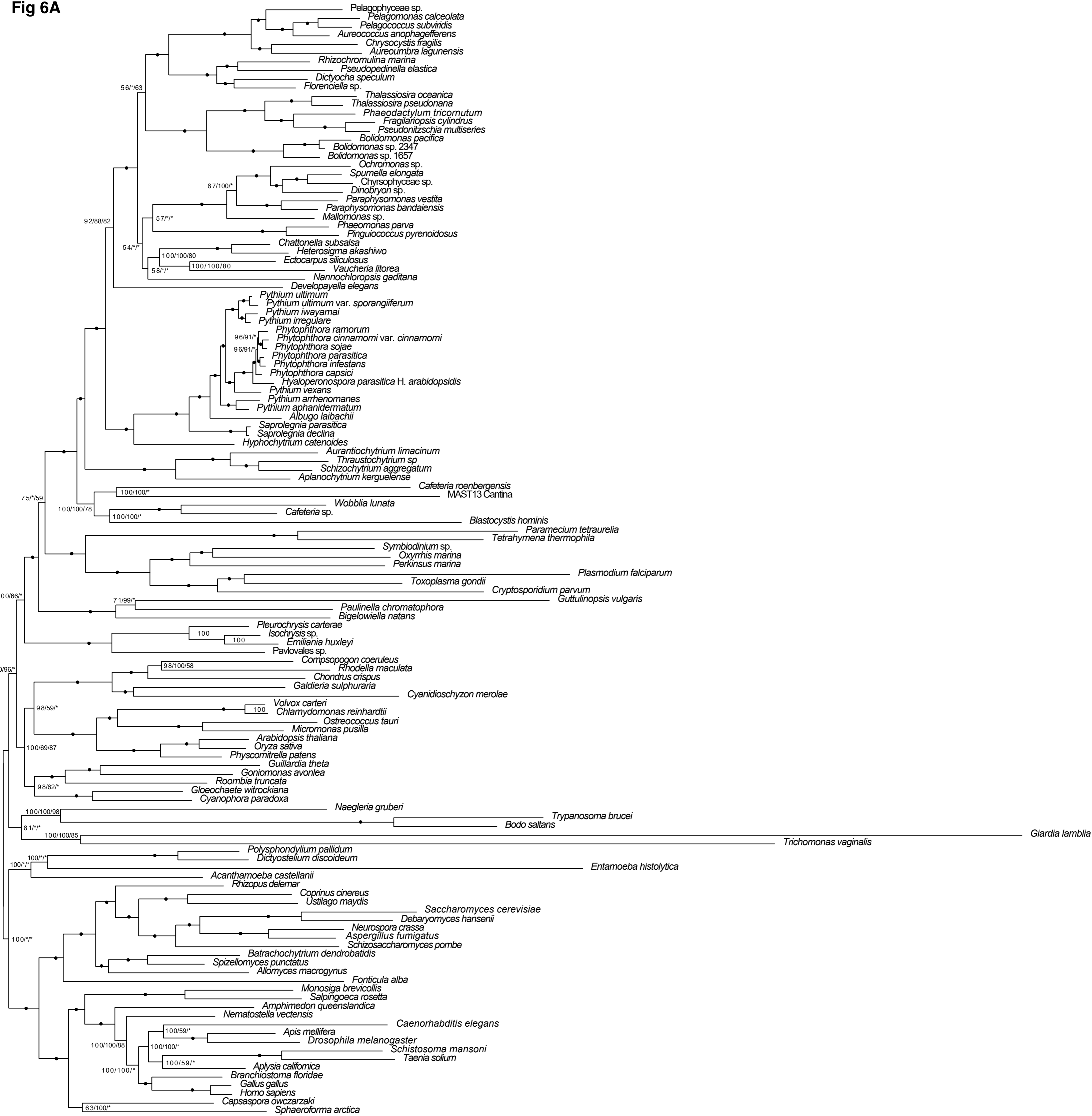

Fig 6B

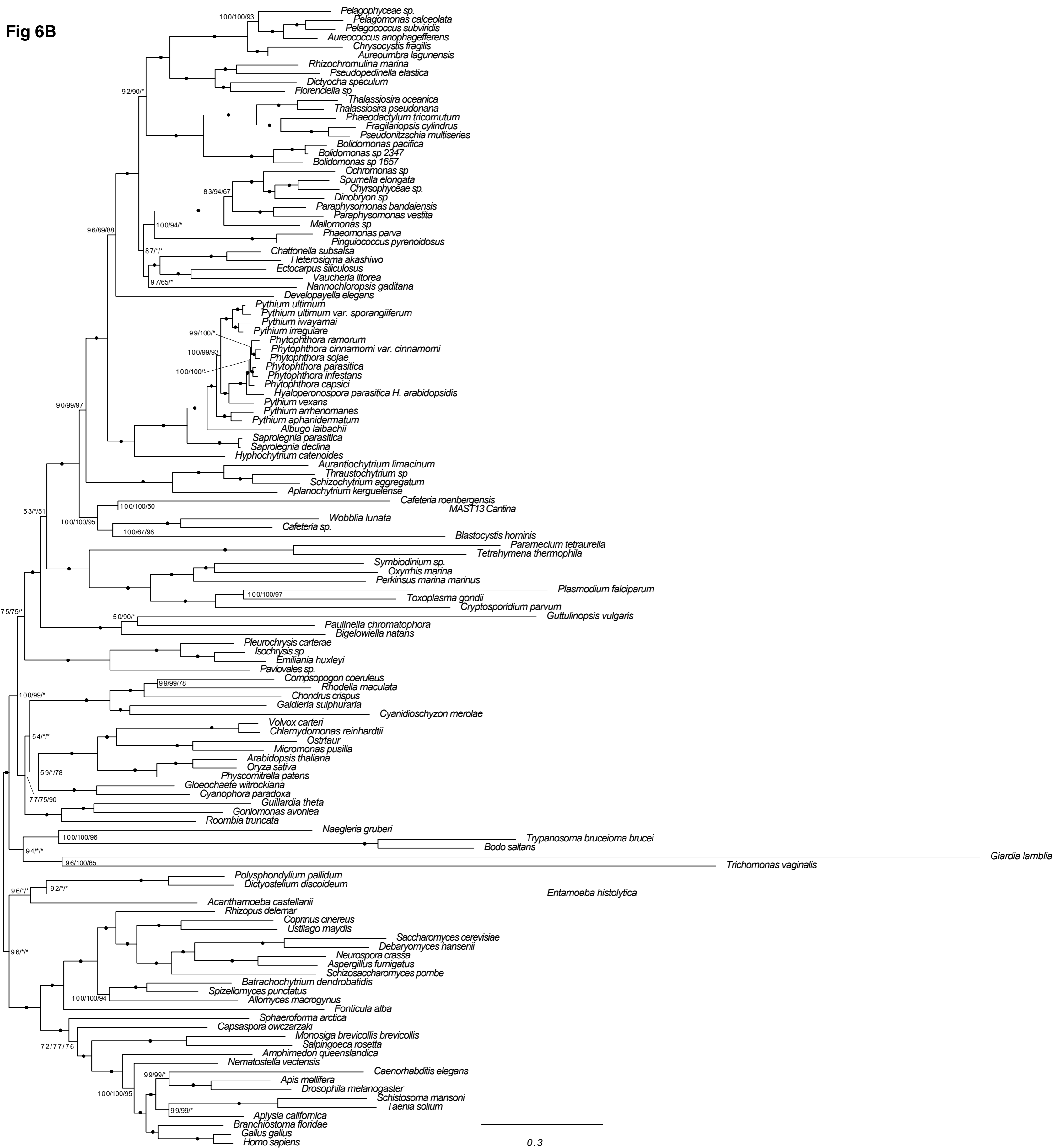

Supplement: Figure S6 [file rsob170184supp7.pdf]

Fig S7A

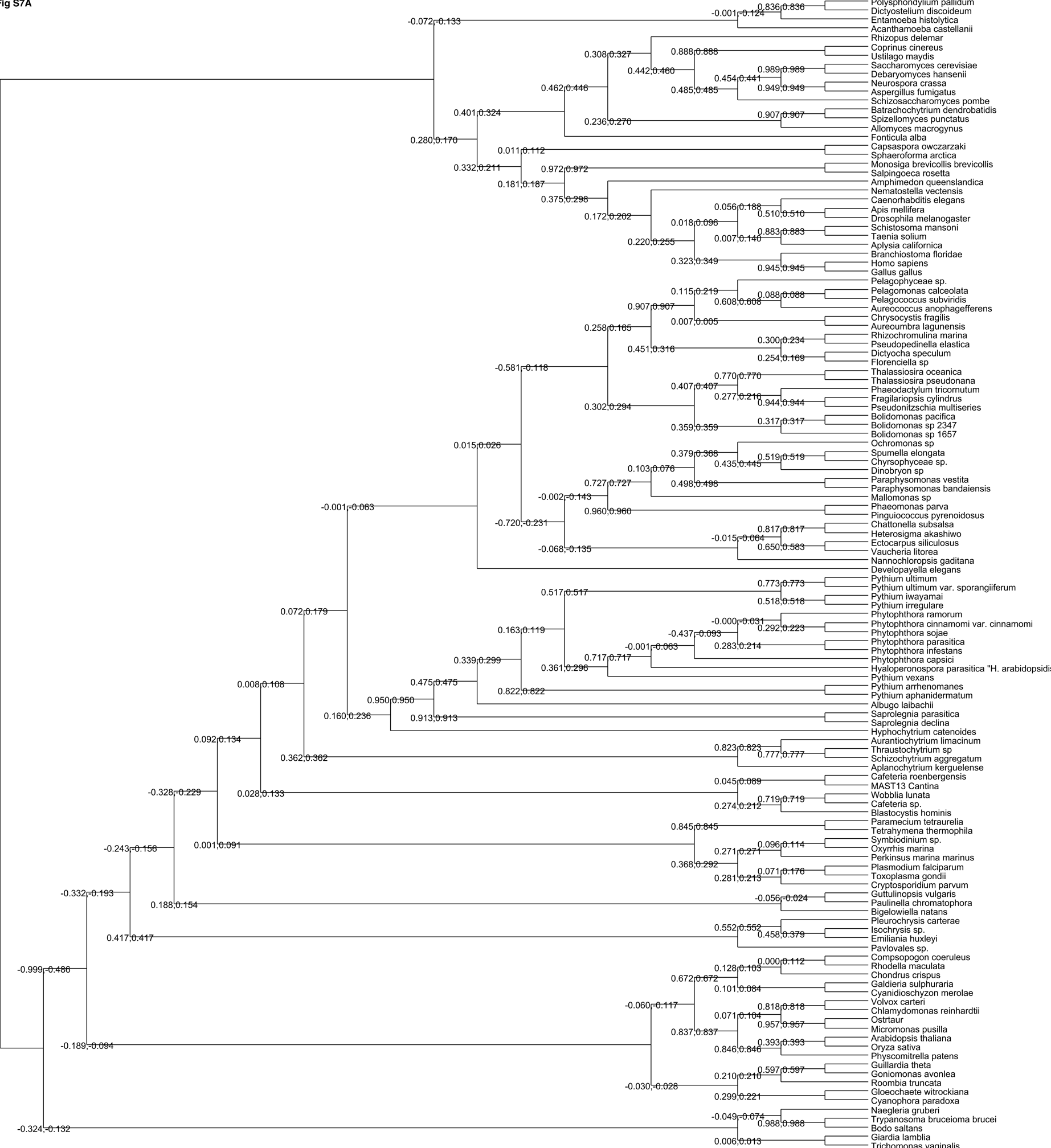

Fig S7B

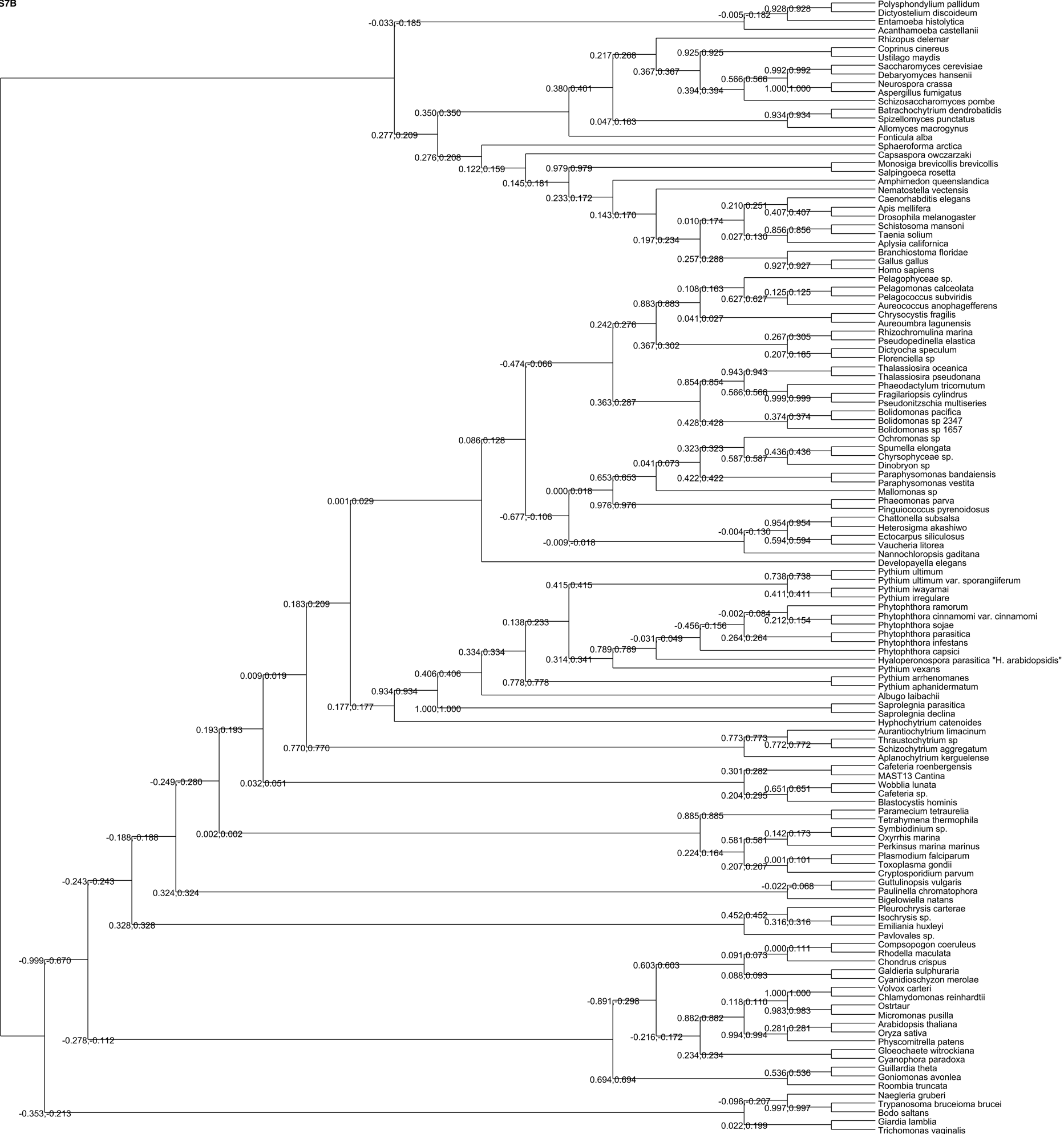

Supplement: Figure S7 [file rsob170184supp8.pdf]

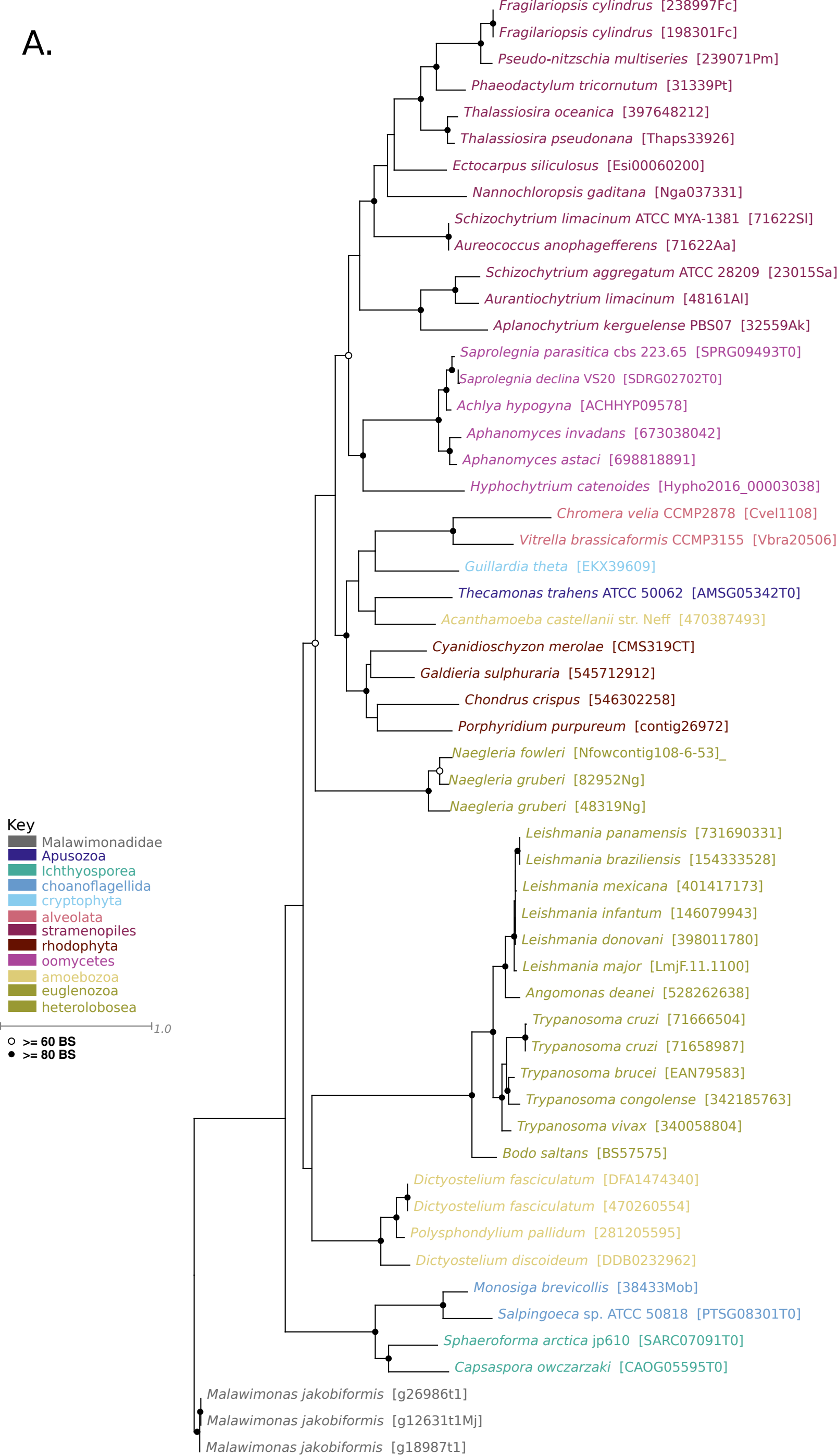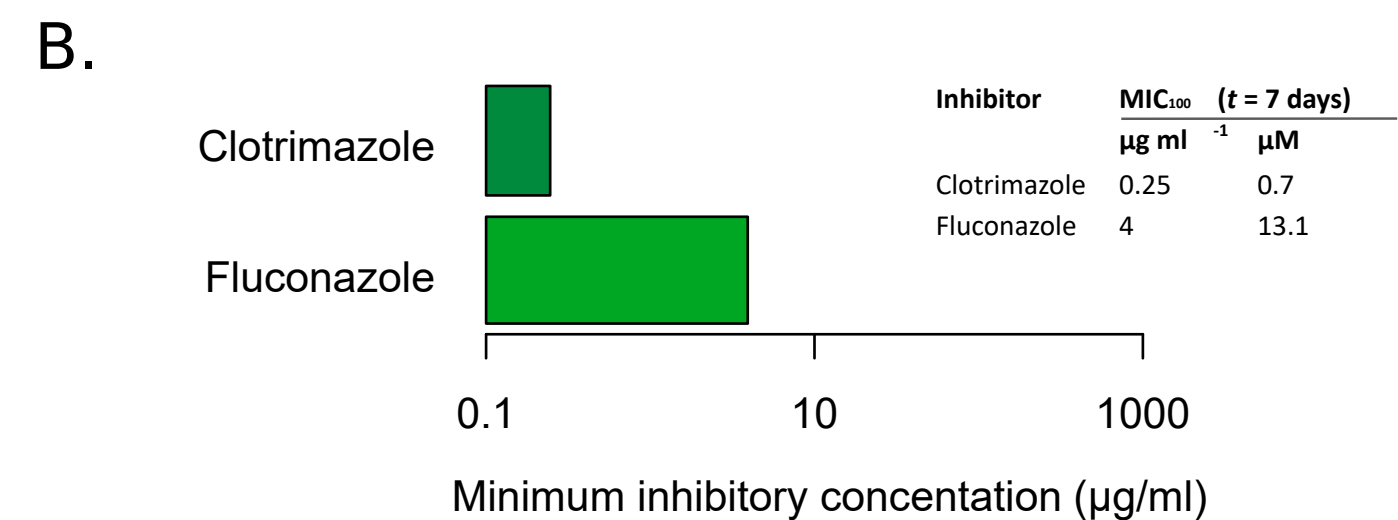

Supplement: Figure S8 [file rsob170184supp9.pdf]

A

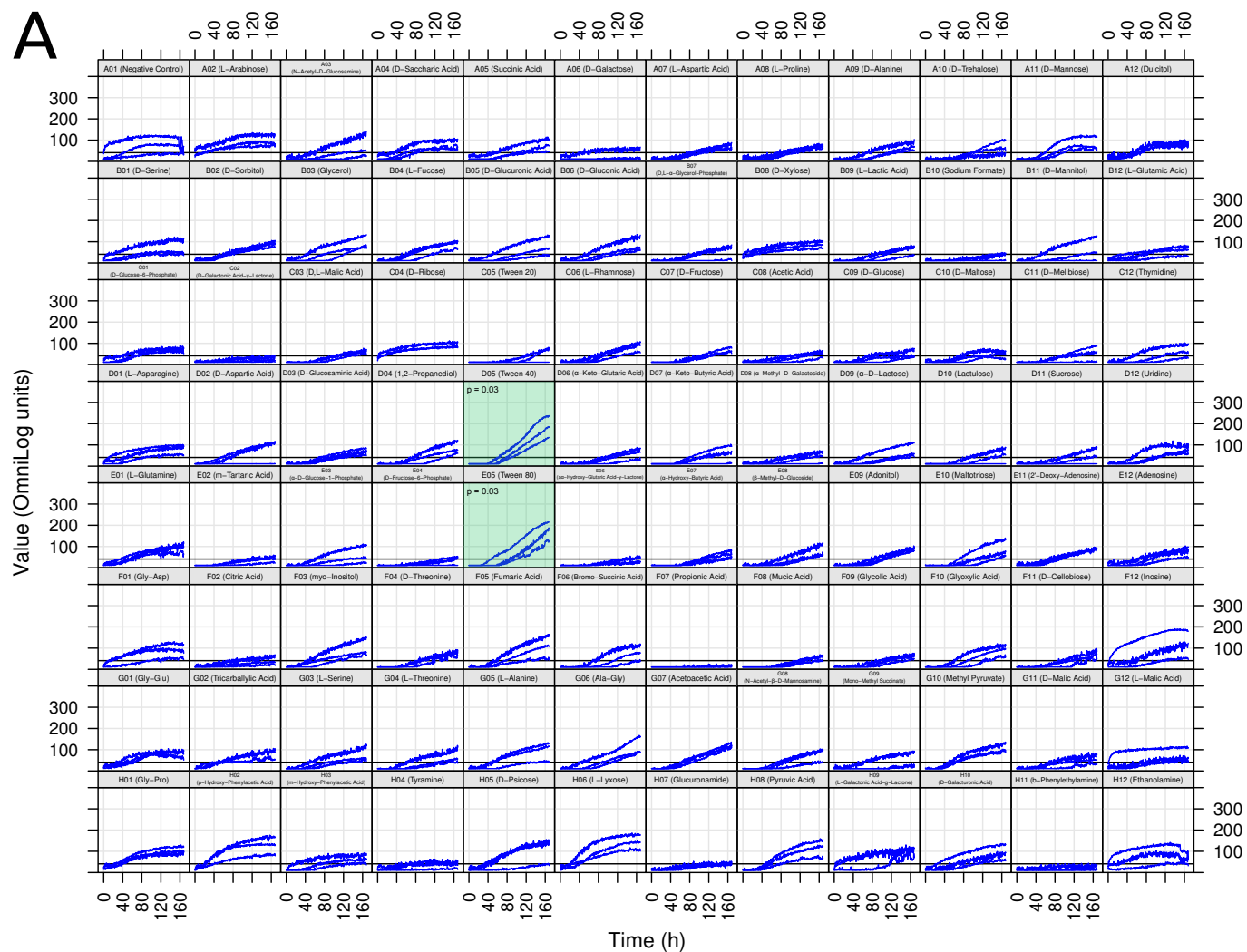

B

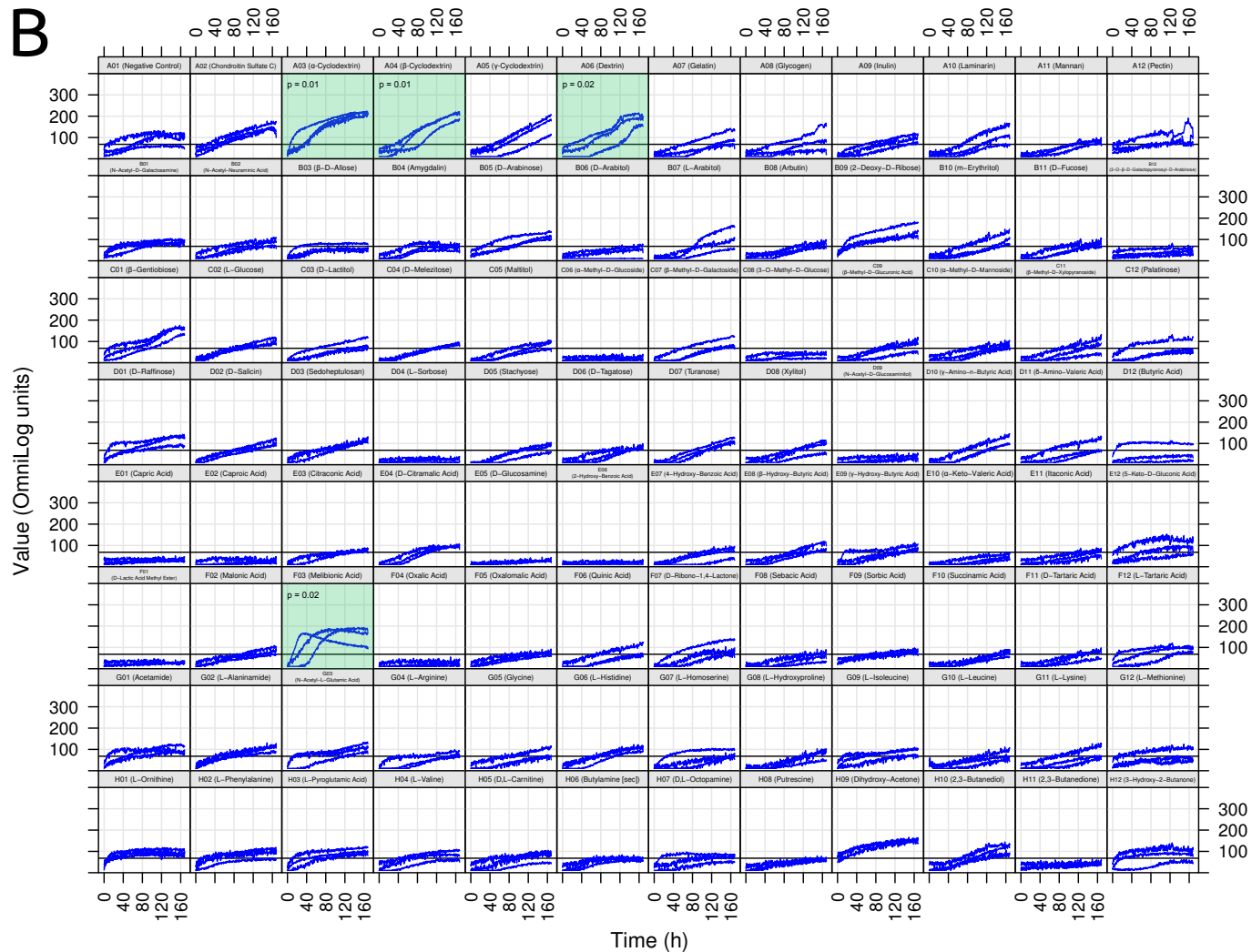

Supplement: Figure S9 [file rsob170184supp10.pdf]

A.

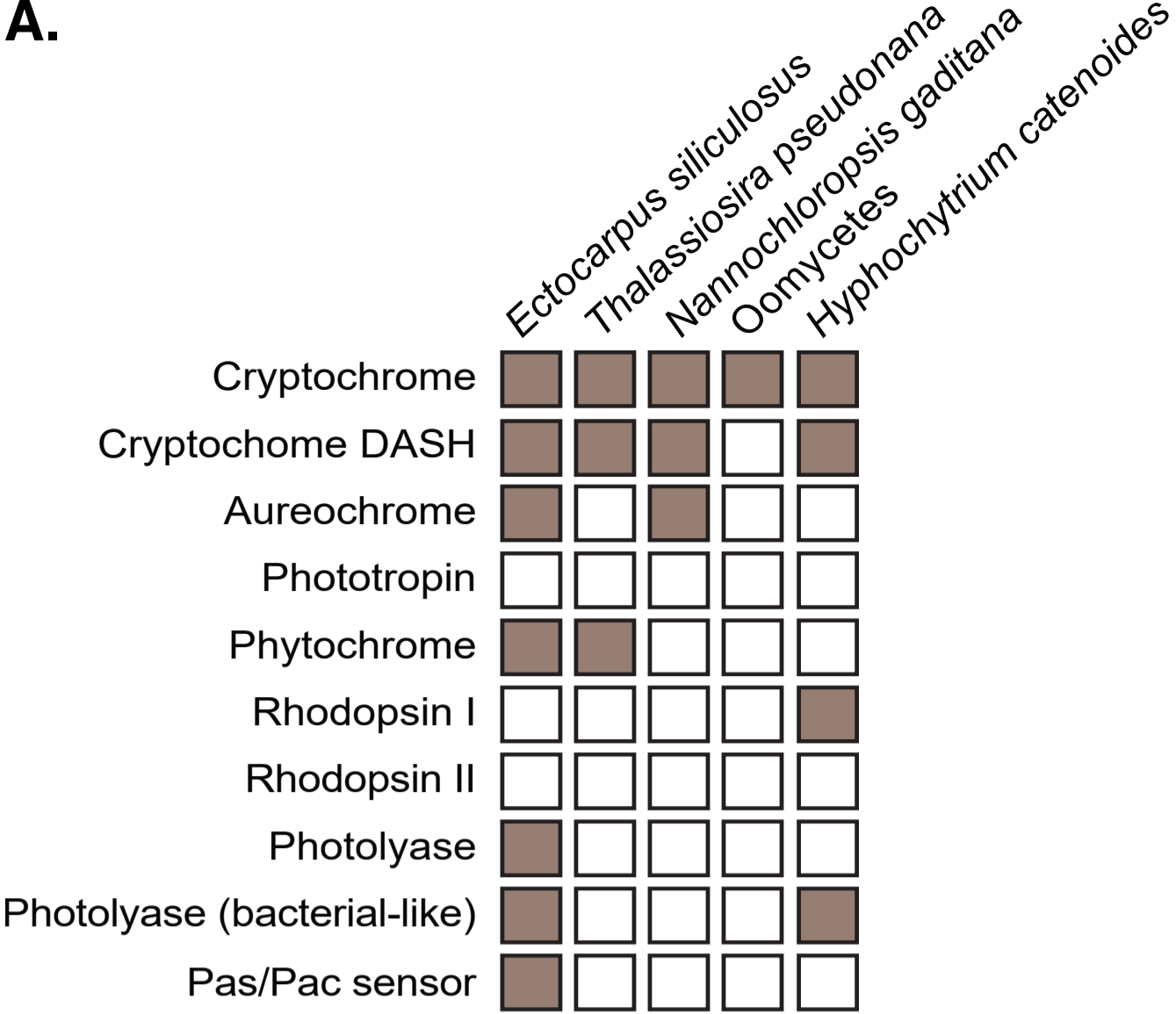

C.

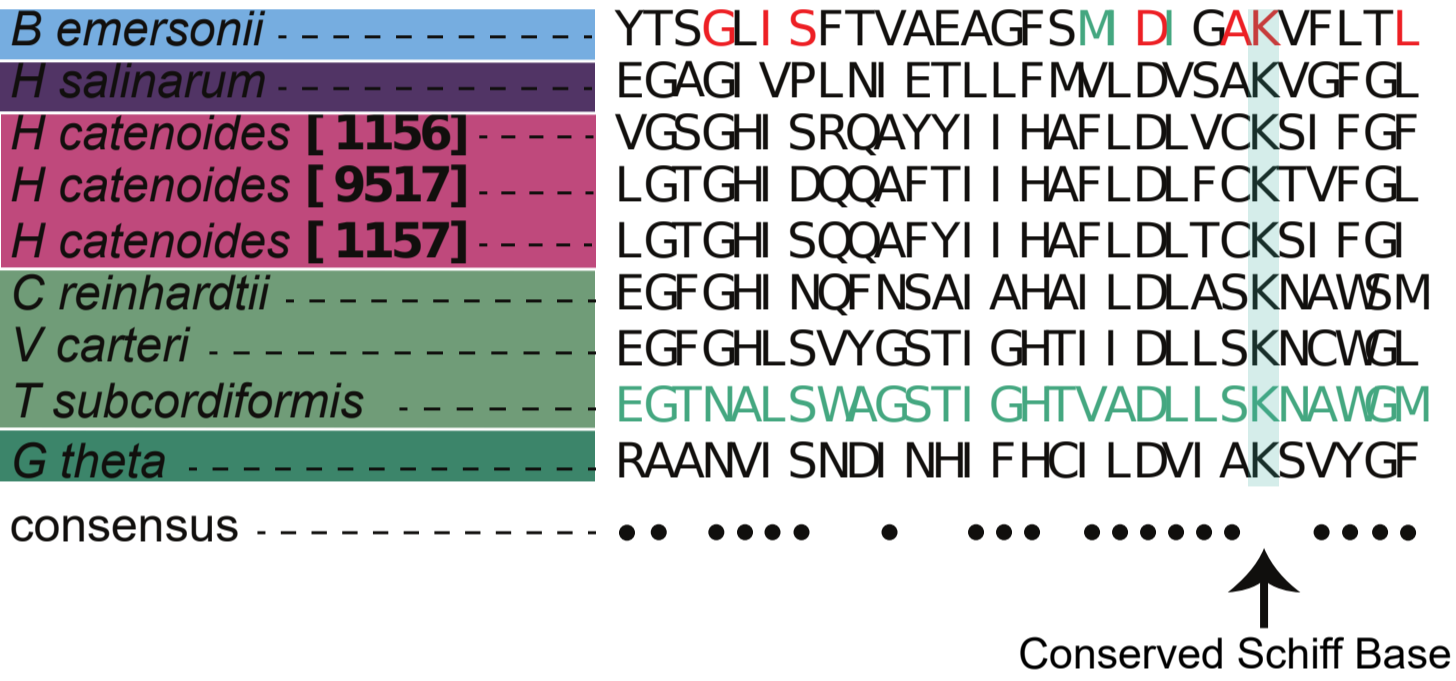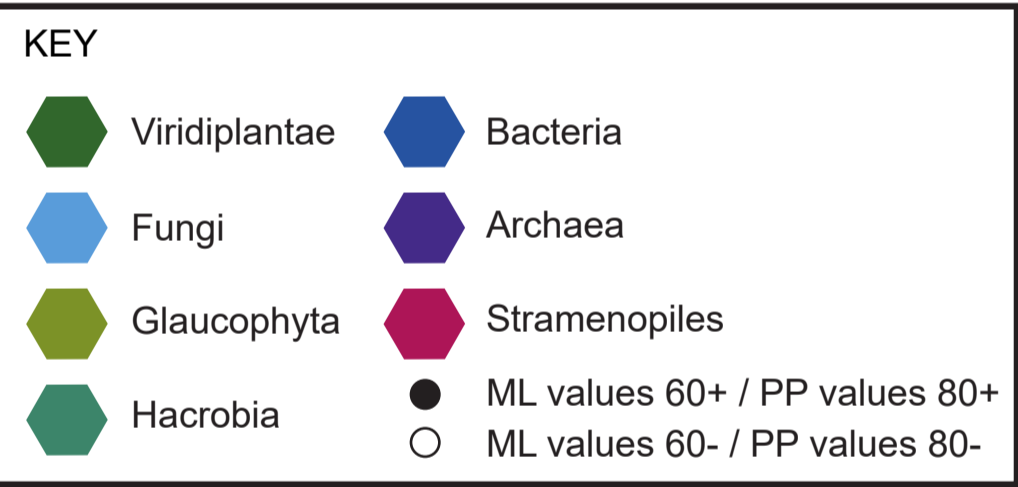

B.

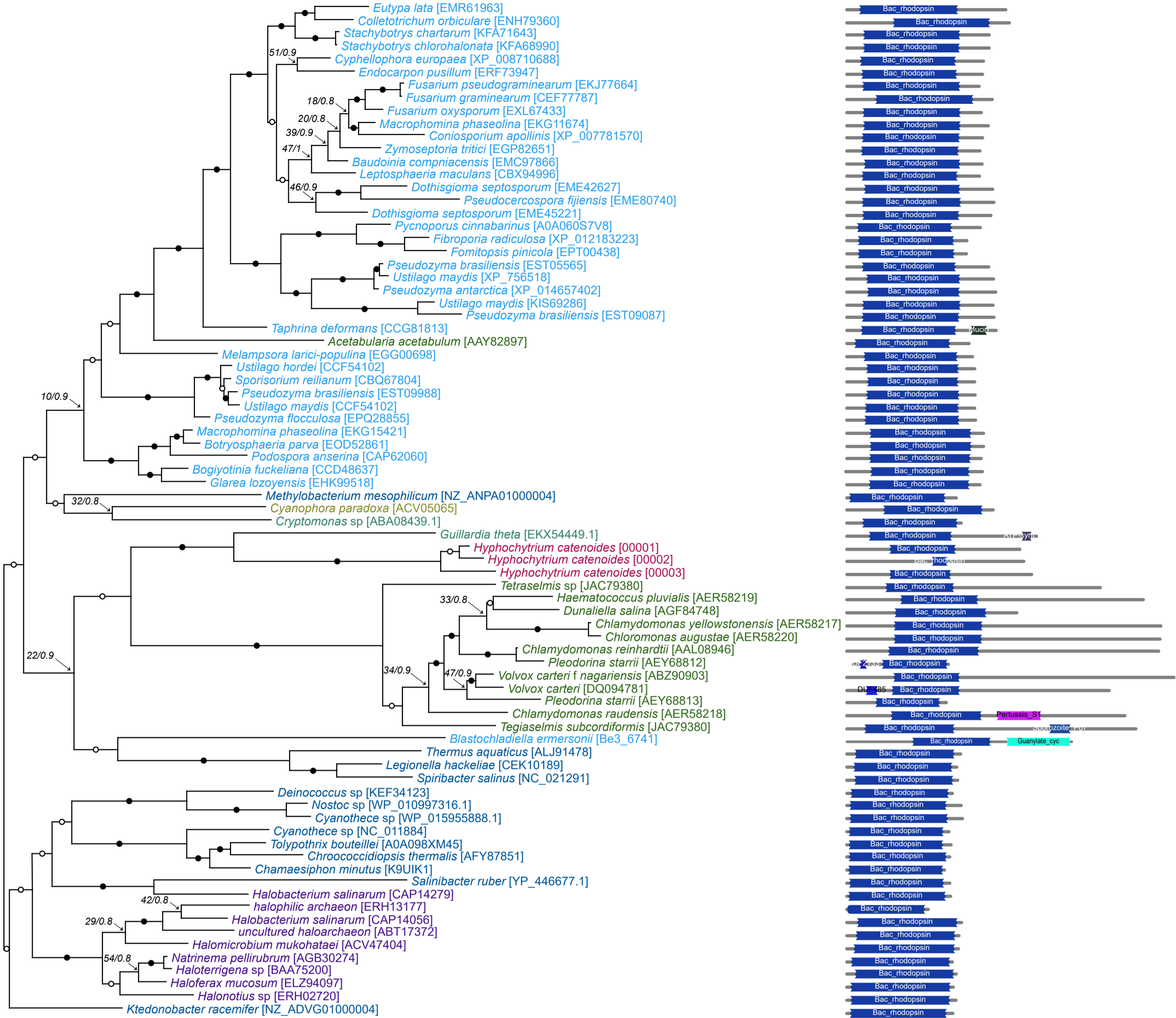

Supplement: Figure S10 [file rsob170184supp11.pdf]

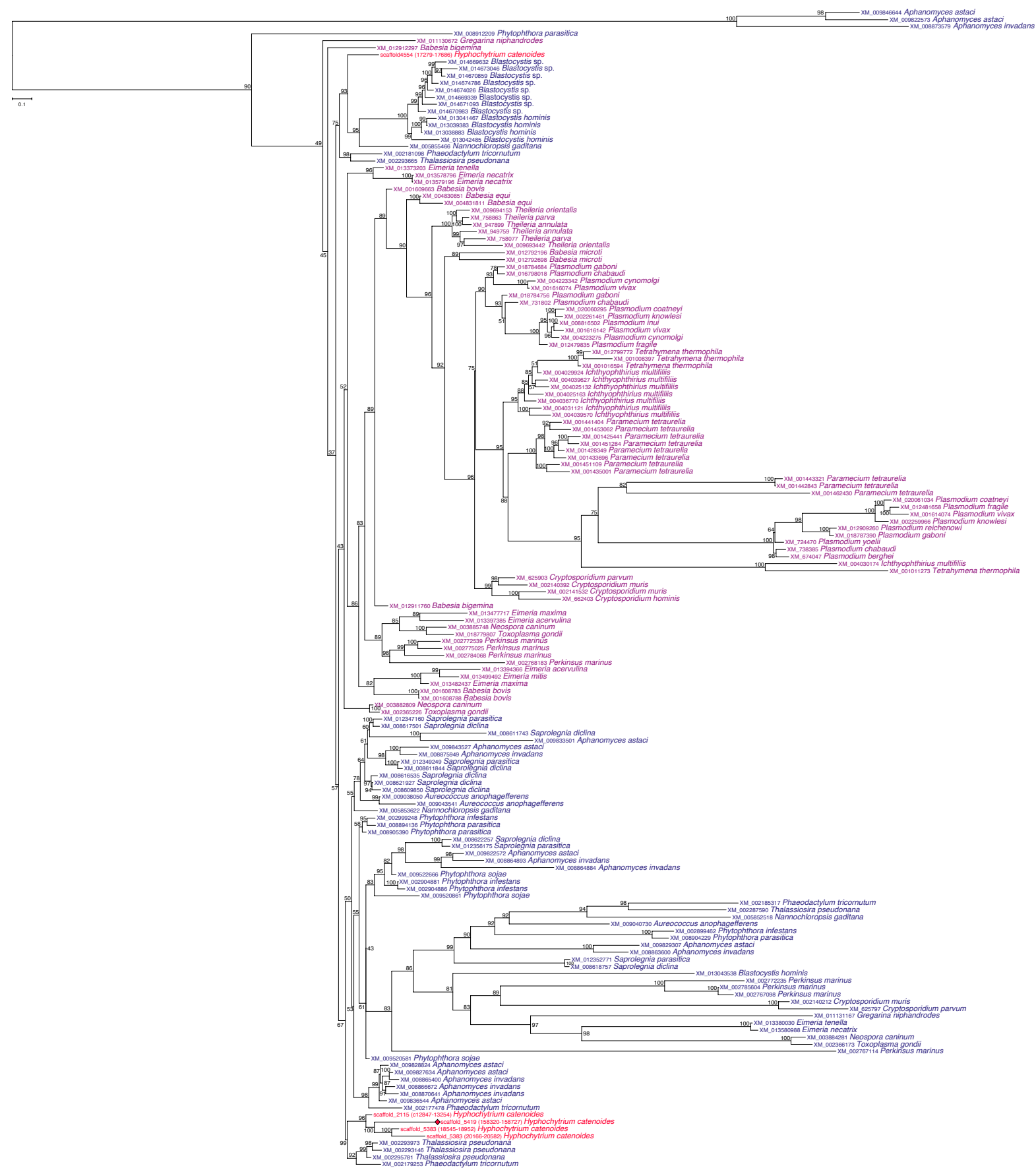

Supplement: Figure S11 [file rsob170184supp12.pdf]

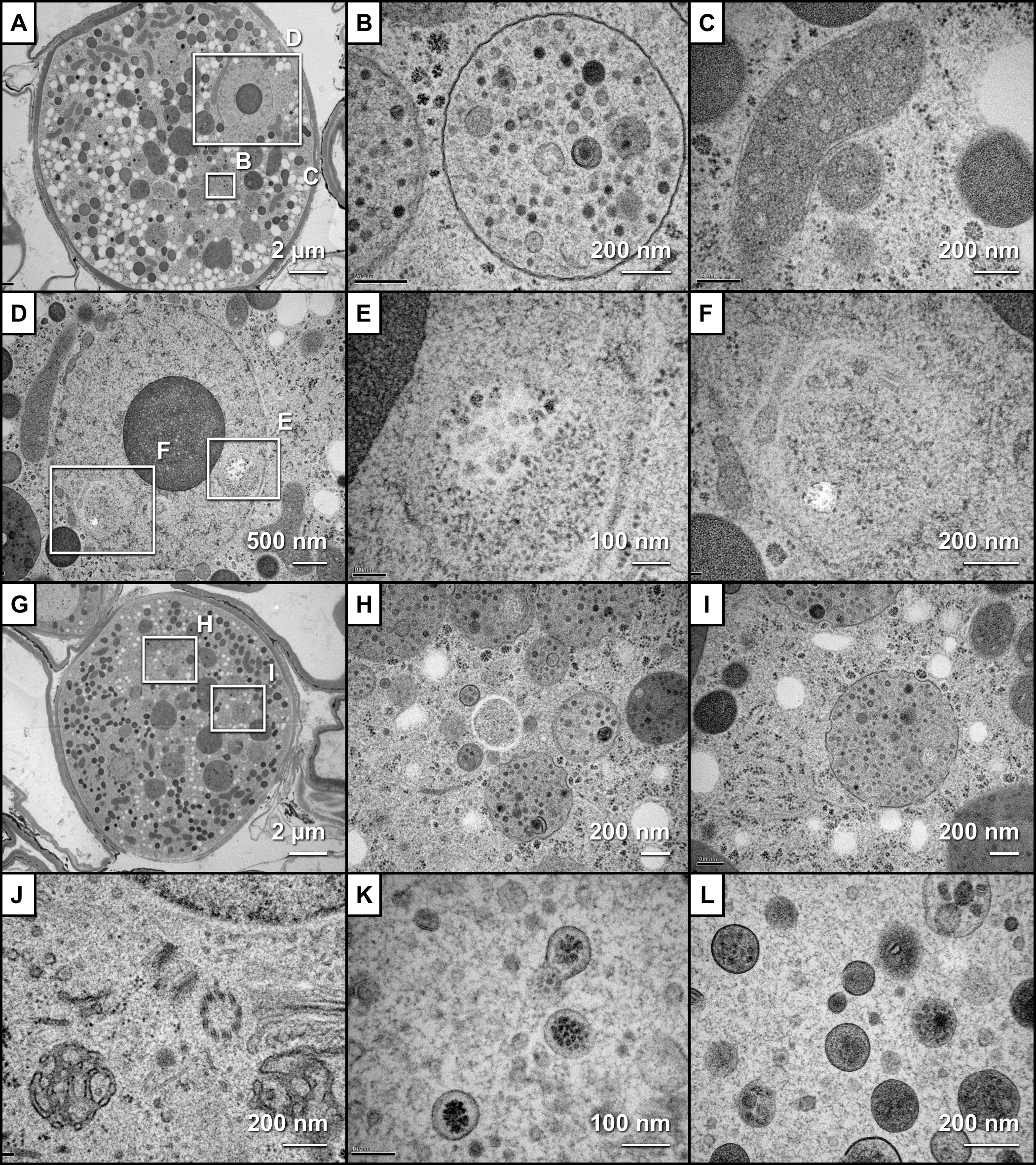

Supplement: Figure S12 [file rsob170184supp13.png]

A.

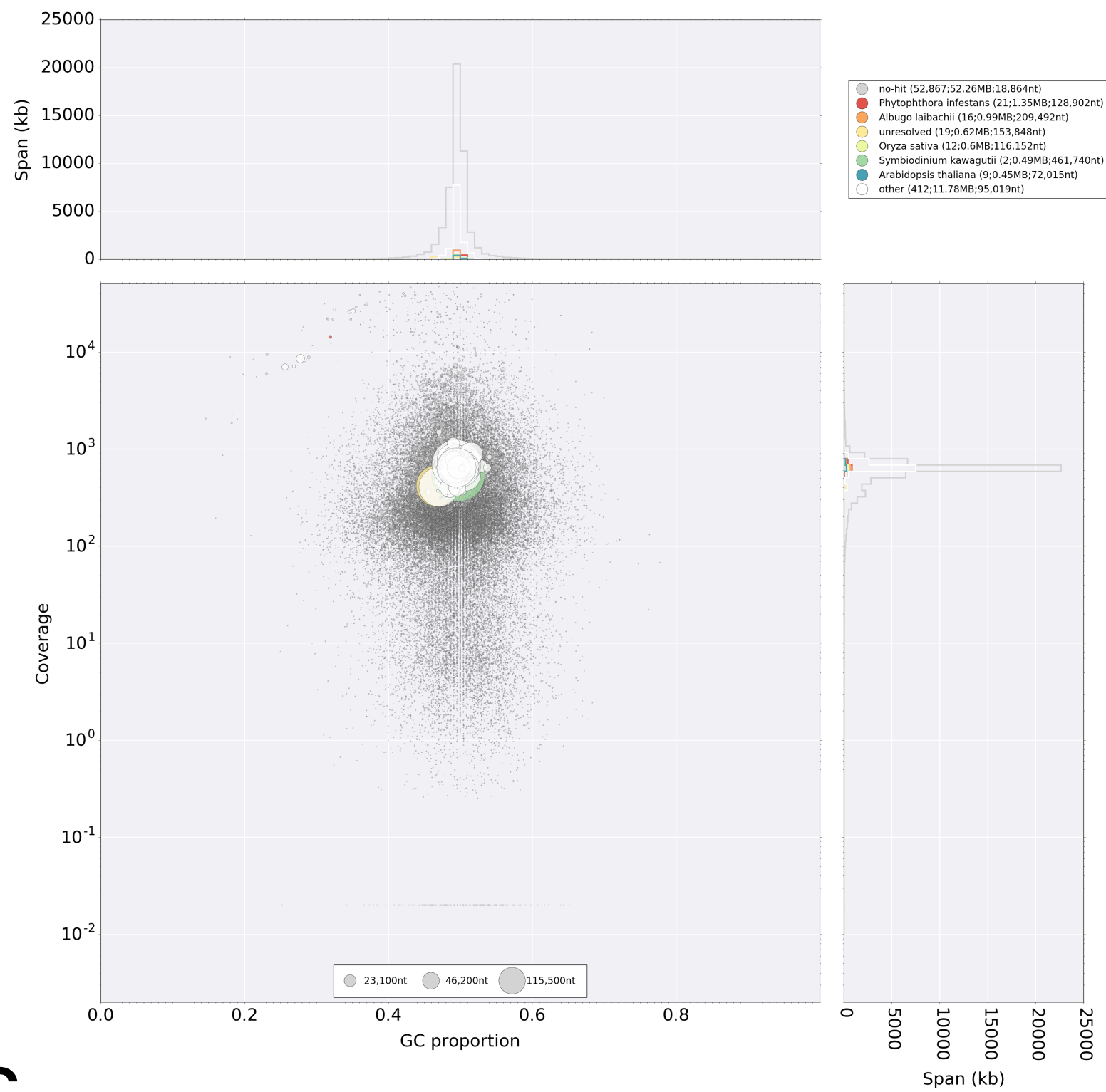

B.

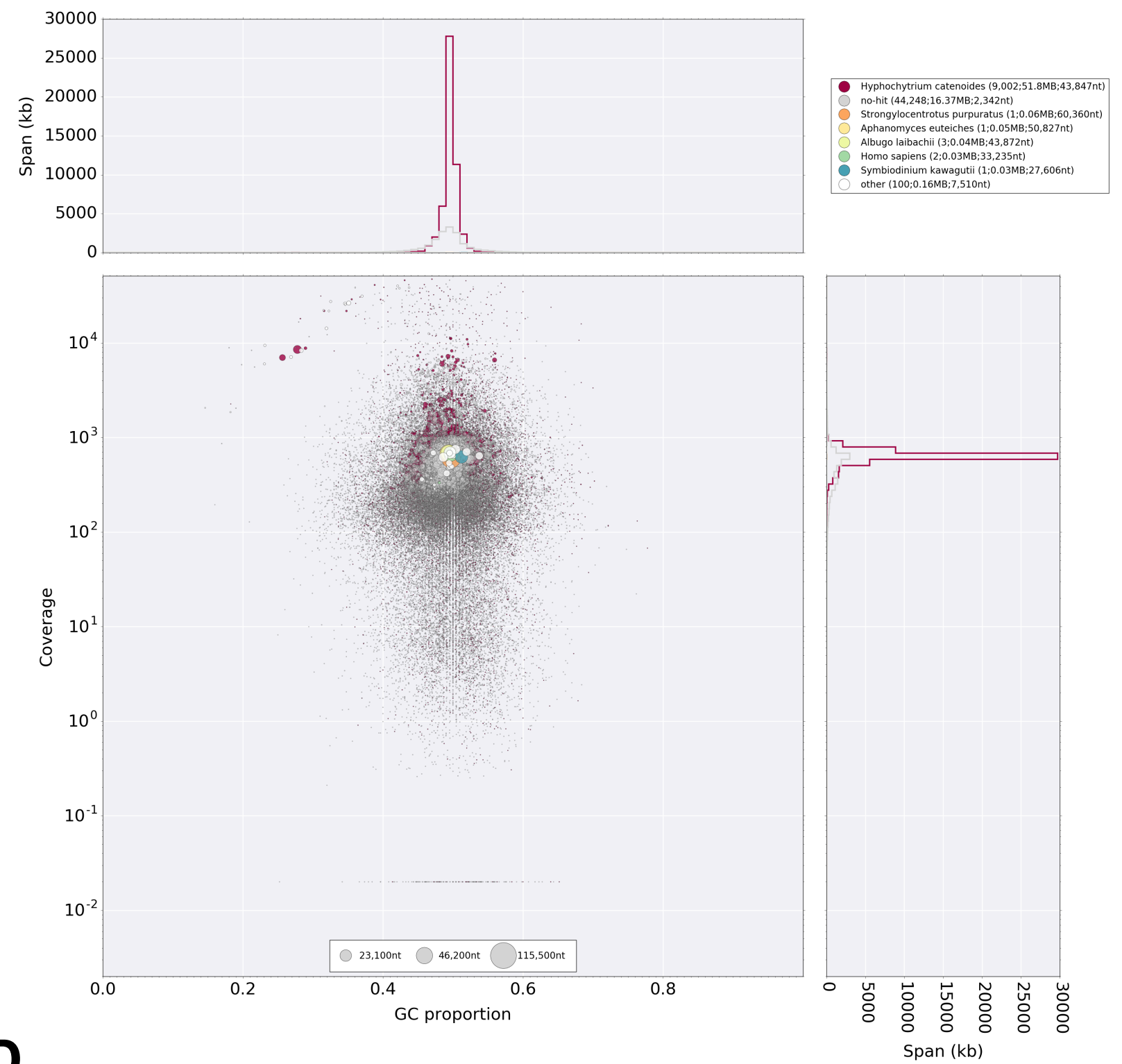

C.

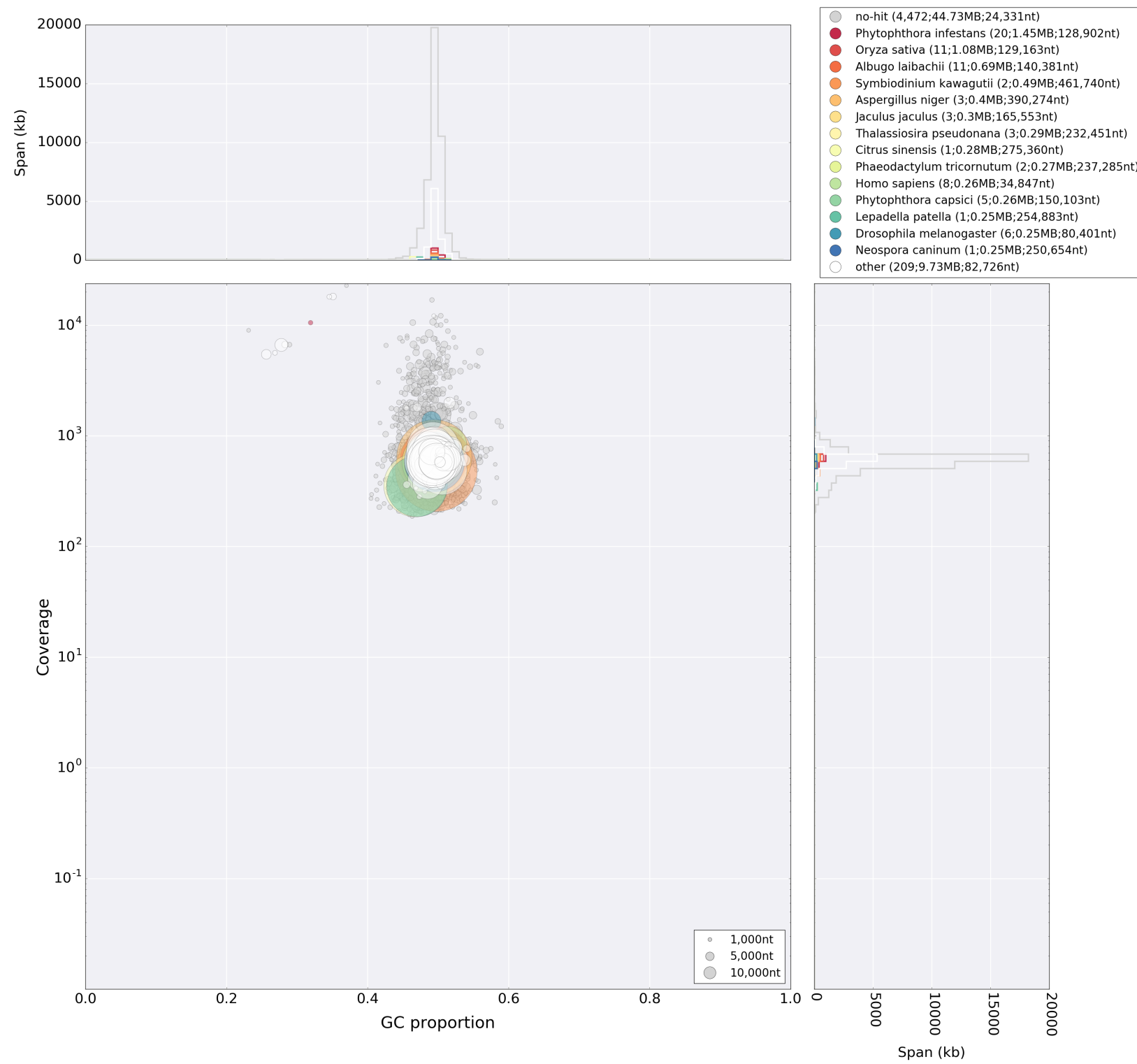

D.

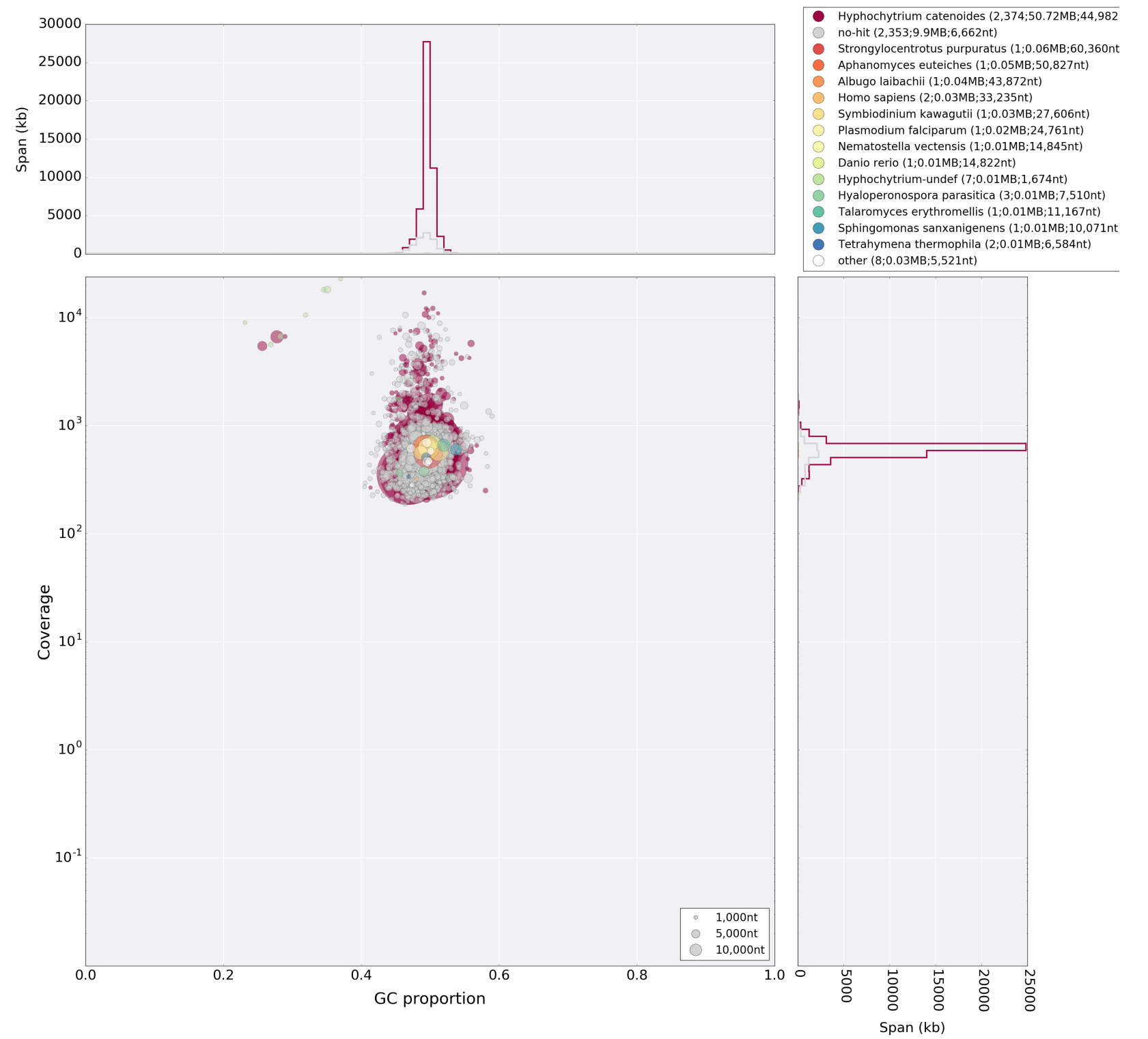

Supplement: Figure S13 [file rsob170184supp14.pdf]

A.

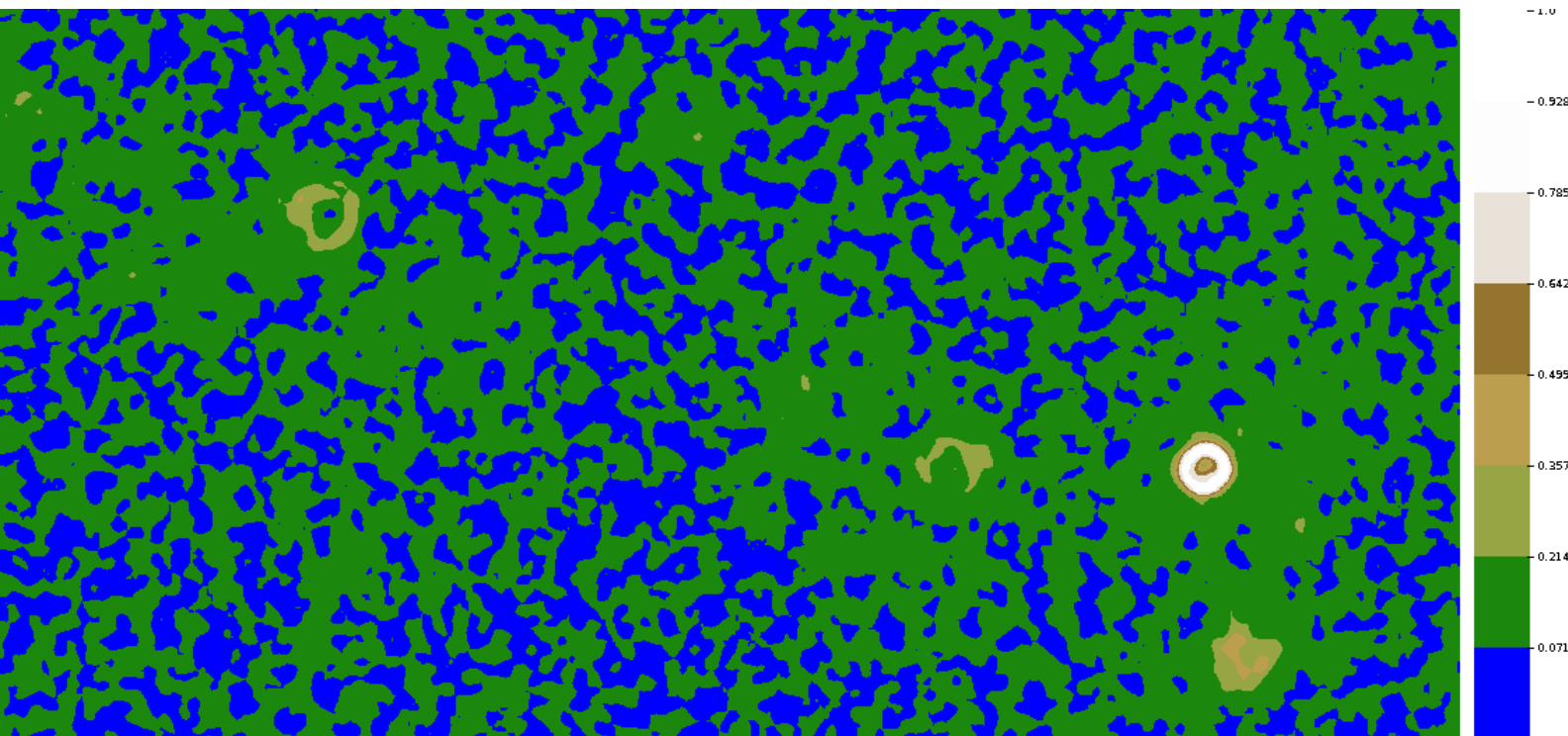

B.

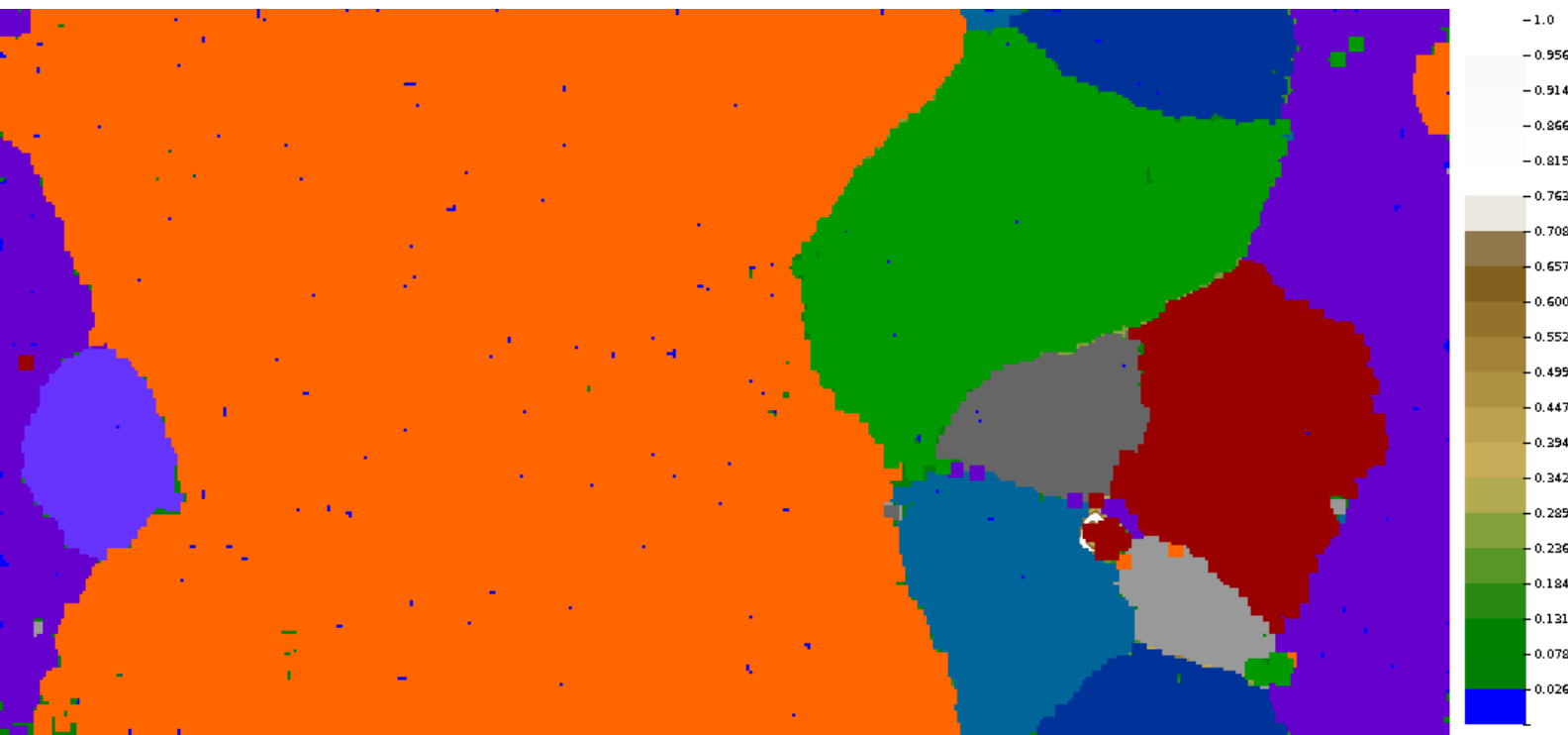

Supplement: Figure S14 [file rsob170184supp15.pdf]

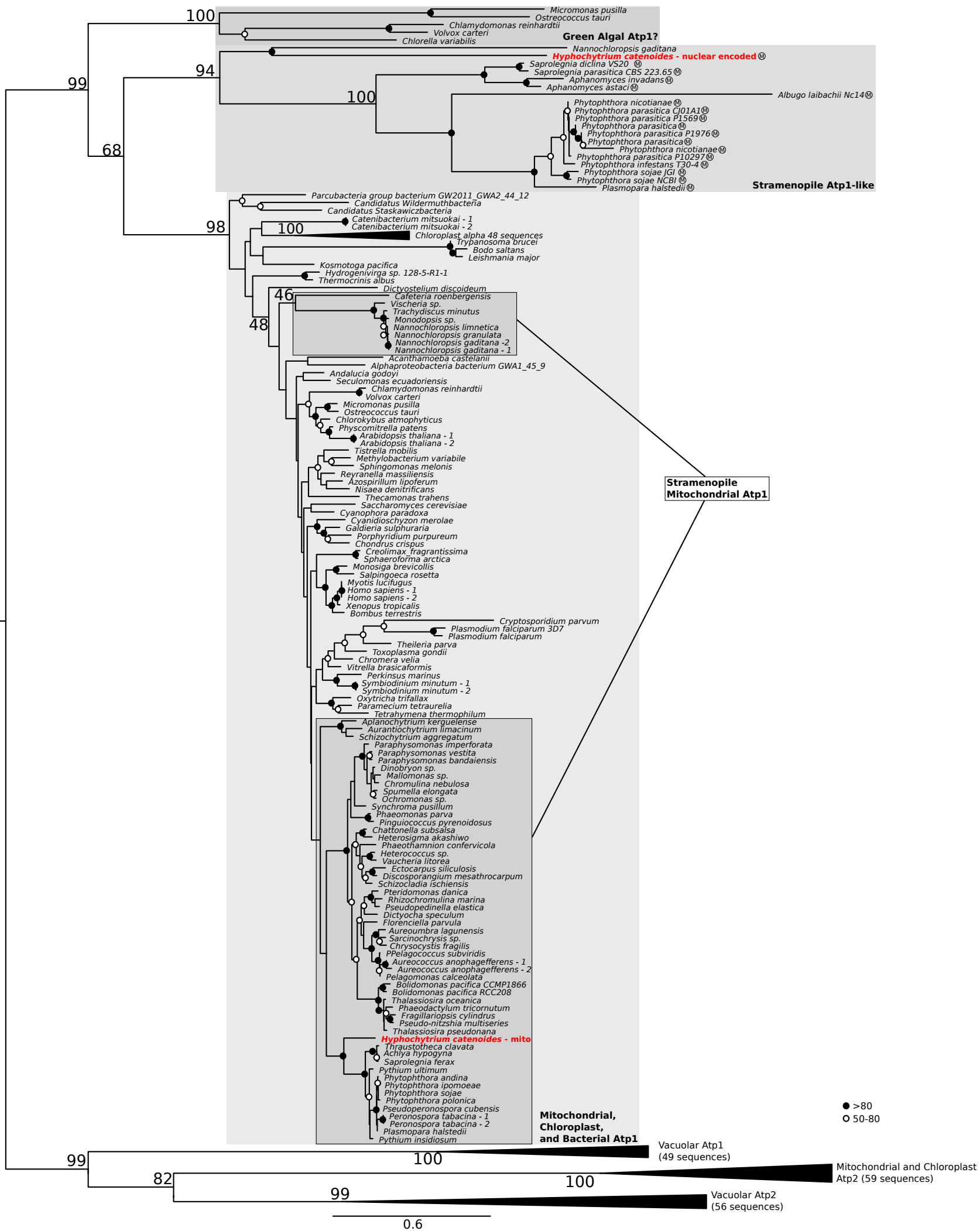

Supplement: Figure S15 [file rsob170184supp16.pdf]
