# Supplementary material for: Comparative genomic analysis of the ‘pseudofungus’ Hyphochytrium catenoides
Source: Open Biol. 2018 Jan 10;8(1):170184. doi: 10.1098/rsob.170184 (PMC5795050; doi:10.1098/rsob.170184)
Supplement: Table S1 [file rsob170184supp17.pdf]

Table S1. Results of reciprocal BLASTp search identifying putative homologues in *Hyphochytrium catenoides* genome of Meiosis functioning gene families (1).

**Meiosis specific genes**	**Seed sequence (*Homo sapiens* [*Hs*] or *Saccharomyces cerevisiae* [*Sc*])**	**Hit in *Hyphochytrium catenoides***	**Reciprocal hit**	**Transcriptome**
SPO11	AAD52562.1 (*Hs*)	Hypho2016_00006629	Yes	Yes
HOP1	NP_012193 (*Sc*, weak hit for *Hs*)	Hypho2016_00001841	Weak	Yes (for weak hit)
HOP2	AHZ60122 (*Sc*, weak hit for *Hs*)	Hypho2016_00004240	Yes	Yes
MND1	NP_011332.2 (*Sc*)	Hypho2016_00011726	Yes	Yes
DMC1	NP_011106.1 (*Sc*)	Hypho2016_00007211	Yes	Yes
MSH4	NP_116652.1 (*Sc*)	Hypho2016_00003686	Yes (possible paralog)	Yes
MSH5	AAA67649.1 (*Sc*)	Hypho2016_00005653	Yes	Yes

1. Ramesh MA, Malik SB, Logsdon JM, Jr. A phylogenomic inventory of meiotic genes; evidence for sex in *Giardia* and an early eukaryotic origin of meiosis. Curr Biol. 2005;15:185-91.
