# Supplementary material for: Comparative genomic analysis of the ‘pseudofungus’ Hyphochytrium catenoides
Source: Open Biol. 2018 Jan 10;8(1):170184. doi: 10.1098/rsob.170184 (PMC5795050; doi:10.1098/rsob.170184)
Supplement: Table S2 [file rsob170184supp18.pdf]

Table S2. Novel Domain Architectures shared across the Pseudofungi.

Gene Name	Domain Architecture	Oomycete exemplar (Accession number)	*Hyphochytrium* homologue (Accession number)
1-phosphatidylinositol 3-phosphate 5-kinase FAB1	FYVE_like_SF-GAF	*Phytophthora nicotianae* (KUF83522)	Hypho2016_00014879
Long-chain-fatty-acid--AMP ligase FadD29	FAAL-PKS_PP-BioF	*Phytophthora nicotianae* (KUF80336)	Hypho2016_00002377
rhodanese domain-containing dual specificity protein phosphatase	DSPc-FYVE_like_SF- RHOD	*Phytophthora nicotianae* (KUF77573)	Hypho2016_00006029
putative aminotransferase	AAT_like-dihydrodipicolinate reductase	*Phytophthora sojae* (XP_009530580)	Hypho2016_00002138
putative callose synthase	Glucan_synthase-Sugar_tr	*Phytophthora infestans* (XP_002998554)	Hypho2016_00001204
conserved hypothetical	RING-finger-tub	*Phytophthora infestans* (XP_002898661)	Hypho2016_00011754
AGC/RSK/RSKP90 protein kinase	PH-PH-PH-STKc_AGC	*Phytophthora infestans* (XP_008915041)	Hypho2016_00001388
phosphatidylinositol 3 and 4-kinase-like protein	PH-FYVE_scVPS27p-PI3Kc_II	*Phytophthora sojae* (XP_009538551)	Hypho2016_00003770
PAX-interacting protein 1	TPR_11-Prefoldin_2	*Phytophthora nicotianae* (KUF96058)	Hypho2016_00008250
histidine kinase A two component receptor	PAS-PAS-PAS-PAS-PAS-PAS-PAS-PAS-PAS-PAS-PAS-PAS-PAS-PAS-PAS-PAS-PAS-Histidine kinase-like ATPases	*Phytophthora sojae* (XP_009526816.1)	Hypho2016_00014859
TKL protein kinase	S_TKc-DEP	*Phytophthora parasitica* (ETM36095)	Hypho2016_00016538
L-aminoadipate-semialdehyde dehydrogenase large subunit	Abhydrolase_6-A_NRPS-SDR_e1	*Phytophthora infestans* (XP_002905400)	Hypho2016_00000654
