# Supplementary material for: Comparative genomic analysis of the ‘pseudofungus’ Hyphochytrium catenoides
Source: Open Biol. 2018 Jan 10;8(1):170184. doi: 10.1098/rsob.170184 (PMC5795050; doi:10.1098/rsob.170184)
Supplement: Table. S3 [file rsob170184supp19.pdf]

Table S4. Putative orthologue families unique to Pseudofungi in comparison to other eukaryotes.

Gene Name	*Hyphochytrium* gene (Accession number)	Oomycete exemplar (Accession number)	Possible provenance of novel gene [and/or characteristic]
RCC1 repeat domain containing protein	Hypho2016_00014105/6	XP_012200151	Novel domain combination
EF-hand domain containing protein	Hypho2016_00009822	XP_008620138.1	Novel domain combination
Catalytic domain of AGC family Serine/Threonine Kinases (STkc_AGC) containing proteinHypho2016_00001388-RA	Hypho2016_00001388	XP_009830367.1*	Novel domain combination
VWD domain containing protein	Hypho2016_00005547	*Pythium* genome sequence PYU1_G013767-201 from Ensembl.	Novel domain combination
Ankyrin repeat and bacterial Toll-like receptor domain protein	Hypho2016_00009105	ETK72757	Novel domain combination
FKBP-type peptidyl-prolyl cis-trans isomerase domain containing protein	Hypho2016_00003951	XP_009828623.1	Novel domain combination
Cysteine-rich domain containing protein	Hypho2016_00003868	AIG56228	Novel protein (no similarity to non-oomycete taxa)
Conserved hypothetical protein	Hypho2016_00005528	CCI42978	Novel protein (no similarity to non-oomycete taxa)
Large eukaryotic DNA virus major capsid protein	Hypho2016_00000981	ETI39689	Viral integration

*Also detected in domain analyses – see Table S
