# Supplementary material for: Comparative genomic analysis of the ‘pseudofungus’ Hyphochytrium catenoides
Source: Open Biol. 2018 Jan 10;8(1):170184. doi: 10.1098/rsob.170184 (PMC5795050; doi:10.1098/rsob.170184)
Supplement: Table S6 [file rsob170184supp22.pdf]

Table S6. PCR to confirm viral gene presence in the genome assembly.

Primer	Sequence (5’-3’)	Within gene?	Amplicon size (bp)
mg96_3prime_overlap_F1	ATGCAACGCGGTCTATTACC	*mg96*	1283
mg96_3prime_overlap_R1	GTAGGCGCTTCACTGGTCTC	-	
mg96_5prime_overlap_R1	CCATATAGCAGCTTGCGTCA	-	1404
mg96_5prime_overlap_F1	CCTAAAGGGTGCCTCCTACC	-	
DNApolB_3prime_overlap_F1	ATCCCAACTTTCAGCCTCCT	*DNA polB*	1334
DNApolB_3prime_overlap_R1	ACAGGAGGTGCGTCCATTAC	*hypothetical*	
DNApolB_5prime_overlap_R1	TTTCTCAGGACGAGGCAGTT	*DNA polB*	1678
DNApolB_5prime_overlap_F1	TGGTACCATGCGACTAACCA	*Predicted carbohydrate binding protein*	
MCP_Hist_3kb_F1	AAGACTGACCTCCGCTTTCA	*histone (intron) [confirms integration in host genome]*	2837
MCP_Hist_3kb_R1	GCTGGATGCCTGTACCTTGT	*mcp*	
MCP_Hist_3kb_F2	AGATCAACGGCTCCATCTCG	*hnh endonuclease*	Primer walking
MCP_Hist_3kb_R2	CTTCGATCAAACCCGCAGAG	Between *hnh* and *mcp*	Primer walking
MCP_3prime_overlap_F1	ACGCATCCAATCCTAACCAG	*mcp*	1299
MCP_3prime_overlap_R1	TCCGCTTTGGAGAAGACACT	*hypothetical*	
