# Supplementary material for: Comparative genomic analysis of the ‘pseudofungus’ Hyphochytrium catenoides
Source: Open Biol. 2018 Jan 10;8(1):170184. doi: 10.1098/rsob.170184 (PMC5795050; doi:10.1098/rsob.170184)
Supplement: Table S8 [file rsob170184supp24.pdf]

Table S9 List of genomes for contamination check

Acanthamoeba castellanii
Aplanochytrium kerguelense
Arabidopsis thaliana
Aurantiochytrium limacinum
Batrachochytrium dendrobatidis
Bigelowiella natans
Blastocystis hominis
Bodo saltans
Caenorhabditis elegans
Capsaspora owczarzaki
Chlamydomonas reinhardtii
Chondrus crispus
Chromera velia
Ciona intestinalis
Cryptococcus neoformans
Cryptosporidium parvum
Cyanidioschyzon merolae
Cyanophora paradoxa
Dictyostelium discoideum
Drosophila melanogaster
Ectocarpus siliculosus
Emiliania huxleyi
Entamoeba histolytica
Fonticula alba
Giardia intestinalis
Guillardia theta
Homo sapiens
Hyaloperonospora arabidopsidis
Klebsormidium flaccidum
Laccaria bicolor

32	Mastigamoeba balamuthi
33	Monocercomonoides sp.
34	Monosiga brevicollis
35	Mortierella verticillata
36	Mus musculus
37	Naegleria gruberi
38	Nannochloropsis gaditana
39	Neurospora crassa
40	Ostreococcus lucimarinus
41	Perkinsus marinus
42	Phaeodactylum tricornutum
43	Physcomitrella patens
44	Phytophthora ramorum
45	Plasmodium falciparum
46	Populus trichocarpa
47	Porphyridium purpureum
48	Reticulomyxa filosa
49	Rhizophagus irregularis
50	Rozella allomycis
51	Saccharomyces cerevisiae
52	Salpingoeca rosetta
53	Schizosaccharomyces pombe
54	Sphaeroforma arctica
55	Symbiodinium minutum
56	Takifugu rubripes
57	Tetrahymena thermophila
58	Thalassiosira pseudonana
59	Thecamonas trahens
60	Toxoplasma gondii
61	Trichomonas vaginalis
62	Trichoplax adhaerens

63 Trypanosoma brucei

64 Tuber melanosporum

65 Ustilago maydis

66 Vitrella brassicaformis

67 Acidianus hospitalis W1

68 Acidilobus saccharovorans 345-15

69 Aciduliprofundum boonei T469

70 Aeropyrum pernix K1

71 Archaeoglobus fulgidus DSM 4304

72 archaeon Loki

73 Caldisphaera lagunensis DSM 15908

74 Caldivirga maquilingensis IC-167

75 Candidatus Caldiarchaeum subterraneum

76 Candidatus Korarchaeum cryptofilum OPF8

77 Candidatus Micrarchaeum acidiphilum ARMAN-2

78 Candidatus Nanosalina sp. J07AB43

79 Candidatus Nanosalinarum sp. J07AB56

80 Candidatus Nitrosopelagicus brevis

81 Candidatus Parvarchaeum acidophilus

82 Cenarchaeum symbiosum A

83 Desulfurococcus fermentans DSM 16532

84 Ferroglobus placidus DSM 10642

85 Ferroplasma acidarmanus fer1

86 Fervidicoccus fontis Kam940

87 Halalkalicoccus jeotgali B3

88 Haloarcula hispanica ATCC 33960

89 Halobacterium salinarum R1

90 Haloferax mediterranei ATCC 33500

91 Halogeometricum borinquense DSM 11551

92 Halomicrobium mukohataei DSM 12286

93 Halopiger xanaduensis SH-6

94 Haloquadratum walsbyi DSM 16790
95 Halorhabdus utahensis DSM 12940
96 Halorubrum lacusprofundi ATCC 49239
97 Haloterrigena turkmenica DSM 5511
98 Halovivax ruber XH-70
99 Hyperthermus butylicus DSM 5456
100 Ignicoccus hospitalis KIN4/I
101 Ignisphaera aggregans DSM 17230
102 Metallosphaera sedula DSM 5348
103 Methanobacterium formicicum DSM 3637
104 Methanobrevibacter ruminantium M1
105 Methanobrevibacter sp. AbM4
106 Methanocaldococcus fervens AG86
107 Methanocella conradii HZ254
108 Methanococcoides burtonii DSM 6242
109 Methanococcus maripaludis S2
110 Methanocorpusculum labreanum Z
111 Methanoculleus bourgensis MS2
112 Methanofollis liminatans DSM 4140
113 Methanohalobium evestigatum Z-7303
114 Methanohalophilus mahii DSM 5219
115 Methanolobus psychrophilus R15
116 Methanomethylovorans hollandica DSM 15978
117 Methanoplanus limicola DSM 2279
118 Methanopyrus kandleri AV19
119 Methanoregula formicicum SMSP
120 Methanosaeta thermophila PT
121 Methanosalsum zhilinae DSM 4017
122 Methanosarcina acetivorans C2A
123 Methanosphaera stadtmanae DSM 3091
124 Methanosphaerula palustris E1-9c

125 Methanospirillum hungatei JF-1

126 Methanothermobacter marburgensis str. Marburg

127 Methanothermococcus okinawensis IH1

128 Methanothermus fervidus DSM 2088

129 Methanotorris igneus Kol 5

130 Nanoarchaeum equitans Kin4-M

131 Natrialba magadii ATCC 43099

132 Natrinema pellirubrum DSM 15624

133 Natronobacterium gregoryi SP2

134 Natronococcus occultus SP4

135 Natronomonas pharaonis DSM 2160

136 Nitrosopumilus maritimus SCM1

137 Picrophilus torridus DSM 9790

138 Pyrobaculum arsenaticum DSM 13514

139 Pyrococcus yayanosii CH1

140 Pyrolobus fumarii 1A

141 Salinarchaeum sp. Harcht-Bsk1

142 Staphylothermus hellenicus DSM 12710

143 Sulfolobus islandicus M.14.25

144 Thermococcus barophilus MP

145 Thermofilum pendens Hrk 5

146 Thermogladius cellulolyticus 1633

147 Thermoplasma acidophilum DSM 1728

148 Thermoplasmatales archaeon BRNA1

149 Thermoproteus uzoniensis 768-20

150 Thermosphaera aggregans DSM 11486

151 Vulcanisaeta distributa DSM 14429

152 Agrobacterium fabrum str. C58

153 Amycolatopsis mediterranei U32

154 Aquifex aeolicus VF5

155 Azotobacter vinelandii CA

156 Azotobacter vinelandii DJ

157 Bacillus anthracis str. Ames

158 Bacillus anthracis str. Sterne

159 Bacillus cereus ATCC 14579

160 Bacillus subtilis subsp. subtilis str. 168

161 Bacillus thuringiensis serovar konkukian str. 97-27

162 Bacteroides thetaiotaomicron VPI-5482

163 Bifidobacterium longum NCC2705

164 Bordetella pertussis Tohama I

165 Borrelia burgdorferi B31

166 Bradyrhizobium diazoefficiens USDA 110

167 Burkholderia cenocepacia

168 Burkholderia dolosa

169 Burkholderia pseudomallei K96243

170 Campylobacter jejuni subsp. jejuni NCTC 11168 = ATCC 700819

171 Caulobacter crescentus CB15

172 Chlamydia trachomatis 434/Bu

173 Chlamydophila pneumoniae CWL029

174 Chlorobium tepidum TLS

175 Chloroflexus aurantiacus J-10-fl

176 Clostridium acetobutylicum ATCC 824

177 Clostridium botulinum A str. Hall

178 Clostridium difficile 630

179 Corynebacterium glutamicum ATCC 13032

180 Coxiella burnetii RSA 493

181 Deinococcus radiodurans R1

182 Desulfovibrio vulgaris str. Hildenborough

183 Dictyoglomus turgidum DSM 6724

184 Enterobacter cloacae subsp. cloacae ATCC 13047

185 Enterococcus faecalis V583

186 Escherichia coli IAI39

187 Escherichia coli O104 H4 str. 2011C-3493

188 Escherichia coli O157 H7 str. Sakai

189 Escherichia coli O83 H1 str. NRG 857C

190 Escherichia coli str. K-12 substr. MG1655

191 Escherichia coli UMN026

192 Flavobacterium psychrophilum JIP02/86

193 Francisella tularensis subsp. tularensis SCHU S4

194 Fusobacterium nucleatum subsp. nucleatum ATCC 25586

195 Geobacter sulfurreducens PCA

196 Gloeobacter violaceus PCC 7421

197 Haemophilus influenzae Rd KW20

198 Helicobacter pylori 26695

199 Ketogulonicigenium vulgare WSH-001

200 Klebsiella pneumoniae subsp. pneumoniae HS11286

201 Lactobacillus acidophilus NCFM

202 Lactobacillus plantarum WCFS1

203 Lactobacillus salivarius UCC118

204 Lactococcus lactis subsp. lactis Il1403

205 Listeria monocytogenes EGD-e

206 Moorella thermoacetica ATCC 39073

207 Mycobacterium bovis AF2122/97

208 Mycobacterium leprae TN

209 Mycoplasma mycoides subsp. mycoides SC str. PG1

210 Pseudomonas aeruginosa PAO1

211 Rhodobacter sphaeroides 2.4.1

212 Rhodopirellula baltica SH 1

213 Rhodospirillum rubrum ATCC 11170

214 Rickettsia prowazekii str. Madrid E

215 Salinibacter ruber DSM 13855

216 Salmonella enterica subsp. enterica serovar Typhi str. CT18

217 Shewanella oneidensis MR-1

218 Shigella dysenteriae Sd197

219 Shigella flexneri 2a str. 301

220 Streptococcus pneumoniae R6

221 Streptococcus suis BM407

222 Thermanaerovibrio acidaminovorans DSM 6589

223 Thermodesulfovibrio yellowstonii DSM 11347

224 Thermosynechococcus elongatus BP-1

225 Thermotoga maritima MSB8

226 Vibrio cholerae O1 biovar El Tor str. N16961

227 Vibrio fischeri ES114

228 Xanthomonas campestris pv. campestris str. ATCC 33913

229 Yersinia enterocolitica subsp. enterocolitica 8081

230 Yersinia pestis CO92
