# Supplementary material for: Comparative genomic analysis of the ‘pseudofungus’ Hyphochytrium catenoides
Source: Open Biol. 2018 Jan 10;8(1):170184. doi: 10.1098/rsob.170184 (PMC5795050; doi:10.1098/rsob.170184)
Supplement: Table S9 [file rsob170184supp25.pdf]

Table S2. Scaffolds that could be contamination. These 9 scaffolds total 31,406 bp

Scaffold ID	Bp (Coverage)	Primary BlastX hit (Accession number)
FLMG01003949	2256 (423)	membrane protein - *Thaumarchaeota archaeon* (WP_042686520)
FLMG01003818	2142 (514)	Amylosucrase - *Anaerolinaceae* bacterium (KUK961670)
FLMG01003472	1484 (720)	hypothetical protein - *Chroococcales cyanobacterium* (WP_045056520)
FLMG01000834	3368 (406)	Large conductance mechanosensitive channel protein MscL - *Thermomonospora curvata* (WP_012851274.1)
FLMG01000928	5162 (527)	Amylosucrase - gammaproteobacteria bacterium NRL1
FLMG01003411	1272 (689)	Threonine synthase - *Pyrinomonas methylaliphatogenes*
FLMG01001818	6899 (332)	Alpha-amylase - *Psychromonas aquimarina*
FLMG01003558	4080 (579)	Aldehyde-activating protein - *Sinorhizobium fredii*
FLMG01000645	4743 (456)	Acetyl-coenzyme A synthetase - Leminorella grimontii
