# Supplementary material for: Comparative genomic analysis of the ‘pseudofungus’ Hyphochytrium catenoides
Source: Open Biol. 2018 Jan 10;8(1):170184. doi: 10.1098/rsob.170184 (PMC5795050; doi:10.1098/rsob.170184)
Supplement: Table S10 [file rsob170184supp26.pdf]

Table S3 Genomes for standard phylogenetic analyses

Aaosphaeria arxii CBS 175.79
Acanthamoeba castellanii str. Neff
Acanthisitta chloris
Achlya hypogyna
Acidianus hospitalis W1
Acidilobus saccharovorans 345-15
Acidomyces richmondensis
Aciduliprofundum boonei T469
Acremonium alcalophilum
Acromyrmex echinatior
Acropora digitifera
Acyrthosiphon pisum
Acytostelium subglobosum
Aedes aegypti
Aegilops tauschii
Aeropyrum pernix K1
Agaricostilbum hyphaenes
Agaricus bisporus var. bisporus H97
Agaricus bisporus var. burnettii JB137-S8
Agrobacterium fabrum str. C58
Ailuropoda melanoleuca
Ajellomyces capsulatus NAm1
Ajellomyces dermatitidis ATCC 18188
Ajellomyces dermatitidis ATCC 26199
Ajellomyces dermatitidis ER-3
Ajellomyces dermatitidis SLH14081
Albugo laibachii
Alligator mississippiensis
Alligator sinensis
Allomyces macrogynus atcc 38327

32 Alternaria brassicicola

33 Amanita muscaria Koide BX008

34 Amanita thiersii Skay4041

35 Amborella trichopoda

36 Amniculicola lignicola CBS 123094

37 Amorphotheca resinae

38 Amphimedon queenslandica

39 Amycolatopsis mediterranei U32

40 Anas platyrhynchos

41 Angomonas deanei

42 Anolis carolinensis

43 Anopheles darlingi

44 Anopheles gambiae

45 Anthostoma avocetta NRRL 3190

46 Antonospora locustae HM-2013

47 Antrodia sinuosa

48 Apaloderma vittatum

49 Aphanomyces astaci

50 Aphanomyces invadans

51 Apiospora montagnei NRRL 25634

52 Apis dorsata

53 Apis florea

54 Apis mellifera

55 Aplanochytrium kerguelense PBS07

56 Aplosporella prunicola CBS 121167

57 Aplysia californica

58 Aptenodytes forsteri

59 Aquifex aeolicus VF5

60 Aquilegia coerulea

61 Arabidopsis halleri

62 Arabidopsis lyrata

63 Arabidopsis thaliana

64 Arachis duranensis

65 Arachis ipaensis

66 Archaeoglobus fulgidus DSM 4304

67 archaeon Loki

68 Armillaria mellea DSM 3731

69 Arthrobotrys oligospora ATCC 24927

70 Arthroderma benhamiae CBS 112371

71 Artomyces pyxidatus

72 Ascaris suum

73 Ascobolus immersus RN42

74 Ascocoryne sarcoides NRRL50072

75 Ascoidea rubescens NRRL Y17699

76 Ashbya gossypii ATCC 10895

77 Aspergillus acidus

78 Aspergillus aculeatus ATCC 16872

79 Aspergillus brasiliensis

80 Aspergillus carbonarius ITEM 5010

81 Aspergillus clavatus NRRL 1

82 Aspergillus flavus NRRL3357

83 Aspergillus fumigatus A1163

84 Aspergillus fumigatus Af293

85 Aspergillus glaucus

86 Aspergillus kawachii IFO 4308

87 Aspergillus nidulans

88 Aspergillus niger ATCC 1015

89 Aspergillus niger CBS 513.88

90 Aspergillus niger NRRL3

91 Aspergillus oryzae RIB40

92 Aspergillus sydowii

93 Aspergillus terreus FGSC A1156

94 Aspergillus terreus NIH2624
95 Aspergillus tubingensis
96 Aspergillus versicolor
97 Aspergillus wentii
98 Aspergillus zonatus
99 Asterochloris sp. Cgr_DA1phos
100 Astyanax mexicanus
101 Atractiellales sp. 95
102 Atta cephalotes
103 Aulographum hederae
104 Aurantiochytrium limacinum
105 Aureobasidium pullulans
106 Aureobasidium pullulans var. melanogenum
107 Aureobasidium pullulans var. namibiae
108 Aureobasidium pullulans var. pullulans EXF-150
109 Aureobasidium pullulans var. subglaciale EXF-2481
110 Aureococcus anophagefferens
111 Auricularia subglabra
112 Azadirachta indica
113 Azotobacter vinelandii CA
114 Azotobacter vinelandii DJ
115 Babesia bovis
116 Babesia equi strain WA
117 Babesia microti
118 Babjeviella inositovora NRRL Y-12698
119 Bacillus anthracis str. Ames
120 Bacillus anthracis str. Sterne
121 Bacillus cereus ATCC 14579
122 Bacillus subtilis subsp. subtilis str. 168
123 Bacillus thuringiensis serovar konkukian str. 97-27
124 Backusella circina FSU 941

125 Bacteroides thetaiotaomicron VPI-5482

126 Bactrocera cucurbitae

127 Bactrocera dorsalis

128 Balaenoptera acutorostrata scammoni

129 Balearica regulorum gibbericeps

130 Bathycoccus prasinos

131 Batrachochytrium dendrobatidis JAM81

132 Baudoinia compniacensis UAMH 10762

133 Beauveria bassiana ARSEF 2860

134 Beta vulgaris subsp. vulgaris

135 Bifidobacterium longum NCC2705

136 Bigelowiella natans

137 Bison bison bison

138 Bjerkandera adusta

139 Blastocladiella emersonii

140 Blastocystis hominis

141 Blumeria graminis

142 Bodo saltans

143 Boechera stricta

144 Boletus edulis

145 Bombus impatiens

146 Bombus terrestris

147 Bombyx mori

148 Bordetella pertussis Tohama I

149 Borrelia burgdorferi B31

150 Bos grunniens

151 Bos mutus

152 Bos taurus

153 Botryobasidium botryosum

154 Botryosphaeria dothidea

155 Botryotinia fuckeliana

156 Botrytis cinerea T4

157 Brachypodium distachyon

158 Bradyrhizobium diazoefficiens USDA 110

159 Branchiostoma floridae

160 Brassica oleracea var. oleracea

161 Brassica rapa

162 Brugia malayi

163 Bubalus bubalis

164 Buceros rhinoceros silvestris

165 Burkholderia cenocepacia

166 Burkholderia dolosa

167 Burkholderia pseudomallei K96243

168 Bursaphelenchus xylophilus

169 Cadophora sp. DSE1049

170 Caenorhabditis brenneri

171 Caenorhabditis briggsae

172 Caenorhabditis elegans

173 Caenorhabditis japonica

174 Caenorhabditis remanei

175 Cajanus cajan

176 Calcarisporiella thermophila

177 Caldisphaera lagunensis DSM 15908

178 Caldivirga maquilingensis IC-167

179 Calliarthron tuberculosum

180 Callithrix jacchus

181 Callorhinchus milii

182 Calocera cornea

183 Calocera viscosa

184 Calypte anna

185 Camelina sativa

186 Camelus bactrianus

187 Camelus dromedarius

188 Camelus ferus

189 Camponotus floridanus

190 Campylobacter jejuni subsp. jejuni NCTC 11168 = ATCC 700819

191 Candida albicans sc5314

192 Candida albicans WO-1

193 Candida arabinofermentans NRRL YB-2248

194 Candida caseinolytica NRRL Y-17796

195 Candida dubliniensis CD36

196 Candida glabrata

197 Candida guilliermondii

198 Candida lusitaniae

199 Candida orthopsilosis

200 Candida parapsilosis

201 Candida tanzawaensis NRRL Y-17324

202 Candida tenuis NRRL Y-1498

203 Candida tropicalis

204 Candidatus Caldiarchaeum subterraneum

205 Candidatus Korarchaeum cryptofilum OPF8

206 Candidatus Micrarchaeum acidiphilum ARMAN-2

207 Candidatus Nanosalina sp. J07AB43

208 Candidatus Nanosalinarum sp. J07AB56

209 Candidatus Nitrosopelagicus brevis

210 Candidatus Parvarchaeum acidophilus

211 Canis familiaris

212 Capitella teleta

213 Capra hircus

214 Caprimulgus carolinensis

215 Capronia coronata CBS 617.96

216 Capronia epimyces CBS 606.96

217 Capsaspora owczarzaki

218 Capsella grandiflora

219 Capsella rubella

220 Capsicum annuum

221 Capsicum annuum L.

222 Capsicum annuum var. glabriusculum

223 Cariama cristata

224 Carica papaya

225 Castanea mollissima

226 Catenaria anguillulae PL171

227 Catharanthus roseus

228 Cathartes aura

229 Caulobacter crescentus CB15

230 Cavia porcellus

231 Cenarchaeum symbiosum A

232 Cenococcum geophilum 1.58

233 Cerapachys biroi

234 Ceratitis capitata

235 Ceratotherium simum

236 Cercospora zeae-maydis

237 Ceriporiopsis subvermispora B

238 Cerrena unicolor

239 Chaetomium globosum

240 Chaetomium thermophilum

241 Chaetura pelagica

242 Chalara longipes BDJ

243 Charadrius vociferus

244 Chelonia mydas

245 Chinchilla lanigera

246 Chlamydia trachomatis 434_Bu

247 Chlamydomonas reinhardtii

248 Chlamydophila pneumoniae CWL029

249 Chlamydotis undulata macqueenii

250 Chlorella variabilis NC64A

251 Chlorella vulgaris C-169

252 Chlorobium tepidum TLS

253 Chlorocebus sabaeus

254 Chloroflexus aurantiacus J-10-fl

255 Choiromyces venosus 120613-1

256 Choloepus hoffmanni

257 Chondrus crispus

258 Chromera velia CCMP2878

259 Chrysemys picta bellii

260 Chrysochloris asiatica

261 Cicer arietinum

262 Cicer arietinum L.

263 Ciona intestinalis

264 Ciona savignyi

265 Citrullus lanatus

266 Citrullus lanatus subsp. Vulgaris

267 Citrus clementina

268 Citrus sinensis

269 Cladonia grayi

270 Cladophialophora carrionii CBS 160.54

271 Cladophialophora psammophila CBS 110553

272 Cladophialophora yegresii CBS 114405

273 Cladosporium fulvum

274 Clonorchis sinensis

275 Clostridium acetobutylicum ATCC 824

276 Clostridium botulinum A str. Hall

277 Clostridium difficile 630

278 Coccidioides immitis H538.4

279 Coccidioides immitis RMSCC 2394

280 Coccidioides immitis RMSCC 3703

281 Coccidioides immitis RS

282 Coccidioides posadasii C735 delta SOWgp

283 Coccidioides posadasii RMSCC 3488

284 Coccidioides posadasii Silveira

285 Cochliobolus carbonum 26-R-13

286 Cochliobolus heterostrophus C4

287 Cochliobolus heterostrophus C5

288 Cochliobolus lunatus m118

289 Cochliobolus miyabeanus ATCC 44560

290 Cochliobolus sativus ND90Pr

291 Cochliobolus victoriae FI3

292 Coemansia reversa NRRL 1564

293 Colius striatus

294 Colletotrichum gloeosporioides Nara gc5

295 Colletotrichum higginsianum IMI 349063

296 Colletotrichum orbiculare MAFF 240422

297 Columba livia

298 Condylura cristata

299 Conidiobolus coronatus NRRL 28638

300 Coniochaeta ligniaria

301 Coniophora puteana

302 Coniosporium apollinis CBS 100218

303 Coprinopsis cinerea

304 Coprinopsis cinerea AmutBmut pab1-1

305 Cordyceps militaris CM01

306 Cortinarius glaucopus AT 2004 276

307 Corvus brachyrhynchos

308 Corvus cornix cornix

309 Corynebacterium glutamicum ATCC 13032

310 Corynespora cassiicola Philippines

311 Coxiella burnetii RSA 493

312 Crassostrea gigas

313 Cricetulus griseus

314 Cronartium quercuum f. sp. fusiforme G11

315 Cryphonectria parasitica EP155

316 Cryptococcus gattii R265

317 Cryptococcus gattii WM276

318 Cryptococcus neoformans var. grubii H99

319 Cryptococcus neoformans var. neoformans B-3501A

320 Cryptococcus neoformans var. neoformans JEC21

321 Cryptococcus vishniacii

322 Cryptosporidium hominis

323 Cryptosporidium muris

324 Cryptosporidium parvum

325 Cuculus canorus

326 Cucumis melo L.

327 Cucumis sativus

328 Cucurbitaria berberidis CBS 394.84

329 Culex quinquefasciatus

330 Cyanidioschyzon merolae

331 Cyanophora paradoxa

332 Cyberlindnera jadinii NRRL Y-1542

333 Cylindrobasidium torrendii

334 Cynoglossus semilaevis

335 Dacryopinax sp. DJM-731 SS1

336 Daedalea quercina

337 Daldinia eschscholzii

338 Danaus plexippus

339 Danio rerio

340 Daphnia pulex

341 Dasypus novemcinctus

342 Debaryomyces hansenii

343 Deinococcus radiodurans R1

344 Dekkera bruxellensis CBS 2499

345 Delitschia confertaspora ATCC 74209

346 Dendroctonus ponderosae

347 Dendrothele bispora CBS 962.96

348 Desulfovibrio vulgaris str. Hildenborough

349 Desulfurococcus fermentans DSM 16532

350 Diaphorina citri

351 Dichomitus squalens

352 Dictyoglomus turgidum DSM 6724

353 Dictyostelium discoideum

354 Dictyostelium fasciculatum

355 Dictyostelium purpureum

356 Didymella exigua CBS 183.55

357 Dioszegia cryoxerica

358 Dipodomys ordii

359 Dissoconium aciculare

360 Dothidotthia symphoricarpi

361 Dothistroma septosporum NZE10

362 Drosophila ananassae

363 Drosophila erecta

364 Drosophila grimshawi

365 Drosophila melanogaster

366 Drosophila mojavensis

367 Drosophila persimilis

368 Drosophila pseudoobscura

369 Drosophila sechellia

370 Drosophila simulans

371 Drosophila virilis

372 Drosophila willistoni

373 Drosophila yakuba
374 Echinococcus granulosus
375 Echinococcus multilocularis
376 Echinops telfairi
377 Ectocarpus siliculosus
378 Edhazardia aedis
379 Egretta garzetta
380 Eimeria acervulina
381 Eimeria brunetti
382 Eimeria maxima
383 Eimeria mitis
384 Eimeria necatrix
385 Eimeria praecox
386 Eimeria tenella
387 Elaeis guineensis
388 Elephantulus edwardii
389 Emiliania huxleyi CCMP1516
390 Encephalitozoon cuniculi GB-M1
391 Encephalitozoon hellem ATCC 50504
392 Encephalitozoon intestinalis ATCC 50506
393 Encephalitozoon romaleae SJ-2008
394 Entamoeba dispar
395 Entamoeba histolytica
396 Entamoeba histolytica KU27
397 Entamoeba invadens
398 Entamoeba nuttalli
399 Enterobacter cloacae subsp. cloacae ATCC 13047
400 Enterococcus faecalis V583
401 Enterocytozoon bieneusi H348
402 Eptesicus fuscus
403 Equus caballus

404 Equus przewalskii

405 Eremothecium cymbalariae

406 Erinaceus europaeus

407 Escherichia coli IAI39

408 Escherichia coli O104 H4 str. 2011C-3493

409 Escherichia coli O157 H7 str. Sakai

410 Escherichia coli O83 H1 str. NRG 857C

411 Escherichia coli str. K-12 substr. MG1655

412 Escherichia coli UMN026

413 Esox lucius

414 Eucalyptus grandis

415 Eurotium rubrum

416 Eurypyga helias

417 Eutrema parvulum

418 Eutrema salsugineum

419 Eutypa lata UCREL1

420 Exidia glandulosa

421 Exobasidium vaccinii MPITM

422 Exophiala aquamarina CBS 119918

423 Exophiala dermatitidis

424 Falco cherrug

425 Falco peregrinus

426 Felis catus

427 Ferroglobus placidus DSM 10642

428 Ferroplasma acidarmanus fer1

429 Fervidicoccus fontis Kam940

430 Fibroporia radiculosa TFFH 294

431 Fibulorhizoctonia sp. CBS 109695

432 Ficedula albicollis

433 Fistulina hepatica

434 Flavobacterium psychrophilum JIP02_86

435 Fomitiporia mediterranea

436 Fomitopsis pinicola FP-58527 SS1

437 Fonticula alba

438 Fopius arisanus

439 Fragaria vesca

440 Fragilariopsis cylindrus

441 Francisella tularensis subsp. tularensis SCHU S4

442 Fukomys damarensis

443 Fulmarus glacialis

444 Fusarium fujikuroi

445 Fusarium fujikuroi IMI 58289

446 Fusarium graminearum PH-1

447 Fusarium oxysporum f. sp. melonis

448 Fusarium oxysporum Fo47

449 Fusarium oxysporum FOL 4287

450 Fusarium oxysporum NRRL 32931

451 Fusarium pseudograminearum CS3096

452 Fusarium verticillioides 7600

453 Fusobacterium nucleatum subsp. nucleatum ATCC 25586

454 Gadus morhua

455 Gaeumannomyces graminis var. tritici R3-111a-1

456 Galdieria sulphuraria

457 Galeopterus variegatus

458 Galerina marginata

459 Gallus gallus

460 Ganoderma sp. 10597 SS1

461 Gasterosteus aculeatus

462 Gavia stellata

463 Genlisea aurea

464 Geobacter sulfurreducens PCA

465 Geomyces destructans

466 Geospiza fortis

467 Giardia intestinalis

468 Giardia lamblia

469 Gibberella zeae

470 Glarea lozoyensis ATCC 20868

471 Gloeobacter violaceus PCC 7421

472 Gloeophyllum trabeum

473 Glomerella acutata

474 Glomerella cingulata

475 Glomerella graminicola

476 Glycine max

477 Gonapodya prolifera

478 Gorilla gorilla

479 Gossypium hirsutum

480 Gossypium raimondii

481 Gregarina niphandrodes

482 Grosmannia clavigera kw1407

483 Guillardia theta

484 Gymnascella aurantiaca

485 Gymnascella citrina

486 Gymnopilus chrysopellus PR-1187

487 Gymnopus luxurians

488 Gyrodon lividus BX

489 Haemophilus influenzae Rd KW20

490 Halalkalicoccus jeotgali B3

491 Haliaeetus albicilla

492 Haliaeetus leucocephalus

493 Haloarcula hispanica ATCC 33960

494 Halobacterium salinarum R1

495 Haloferax mediterranei ATCC 33500

496 Halogeometricum borinquense DSM 11551

497 Halomicrobium mukohataei DSM 12286

498 Halopiger xanaduensis SH-6

499 Haloquadratum walsbyi DSM 16790

500 Halorhabdus utahensis DSM 12940

501 Halorubrum lacusprofundi ATCC 49239

502 Haloterrigena turkmenica DSM 5511

503 Halovivax ruber XH-70

504 Hammondia hammondi

505 Hanseniaspora valbyensis NRRL Y-1626

506 Haplochromis burtoni

507 Harpegnathos saltator

508 Hebeloma cylindrosporum h7

509 Helicobacter pylori 26695

510 Heliconius melpomene

511 Helobdella robusta

512 Heterobasidion annosum

513 Heterocephalus glaber

514 Heterogastridium pycnidioideum ATCC MYA-4631

515 Histoplasma capsulatum

516 Homo sapiens

517 Homoloaphlyctis polyrhiza JEL 142

518 Hordeum vulgare

519 Hyaloperonospora arabidopsidis

520 Hydnomerulius pinastri

521 Hydra magnipapillata

522 Hydra vulgaris

523 Hymenolepis microstoma

524 Hyperthermus butylicus DSM 5456

525 Hyphochytrium catenoides

526 Hyphochytrium catenoides old

527 Hypholoma sublateritium

528 Hyphopichia burtonii NRRL Y-1933

529 Hypoxylon sp. CI-4A

530 Hypoxylon sp. CO27-5

531 Hypoxylon sp. EC38

532 Hysterium pulicare

533 Ichthyophthirius multifiliis

534 Ictidomys tridecemlineatus

535 Ignicoccus hospitalis KIN4_I

536 Ignisphaera aggregans DSM 17230

537 Ilyonectria radicicola

538 Ixodes scapularis

539 Jaapia argillacea

540 Jaculus jaculus

541 Jatropha curcas

542 Karstenula rhodostoma CBS 690.94

543 Kazachstania africana

544 Kazachstania naganishii

545 Ketogulonicigenium vulgare WSH-001

546 Klebsiella pneumoniae subsp. pneumoniae HS11286

547 Klebsormidium flaccidum

548 Kluyveromyces lactis

549 Komagataella pastoris

550 Laccaria amethystina LaAM-08-1

551 Laccaria bicolor

552 Lachancea kluyveri

553 Lachancea thermotolerans

554 Lachancea waltii

555 Lactobacillus acidophilus NCFM

556 Lactobacillus plantarum WCFS1

557 Lactobacillus salivarius UCC118

558 Lactococcus lactis subsp. lactis Il1403

559 Laetiporus sulphureus var. sulphureus

560 Larimichthys crocea

561 Latimeria chalumnae

562 Leersia perrieri

563 Leishmania braziliensis

564 Leishmania donovani

565 Leishmania infantum

566 Leishmania major

567 Leishmania mexicana

568 Leishmania panamensis

569 Lentinus tigrinus

570 Lentinus tigrinus ALCF2SS1-6

571 Lentinus tigrinus ALCF2SS1-7

572 Lentithecium fluviatile

573 Lepidopterella palustris

574 Lepisosteus oculatus

575 Leptonychotes weddellii

576 Leptosomus discolor

577 Leptosphaeria maculans

578 Leucoagaricus gongylophorus Ac12

579 Leucogyrophana mollusca

580 Lichtheimia corymbifera

581 Lichtheimia hyalospora

582 Linum usitatissimum

583 Lipomyces starkeyi NRRL Y-11557

584 Lipotes vexillifer

585 Listeria monocytogenes EGD-e

586 Loa loa

587 Lodderomyces elongisporus

588 Lophiostoma macrostomum

589 Lophium mytilinum CBS 269.34

590 Lottia gigantea

591 Lotus japonicus

592 Loxodonta africana

593 Lytechinus variegatus

594 Macaca fascicularis

595 Macaca mulatta

596 Macrolepiota fuliginosa

597 Macrophomina phaseolina MS6

598 Macropus eugenii

599 Macroventuria anomochaeta CBS 525.71

600 Magnaporthe grisea

601 Magnaporthe poae ATCC 64411

602 Malassezia globosa

603 Malassezia sympodialis ATCC 42132

604 Malbranchea cinnamomea

605 Malus domestica

606 Manacus vitellinus

607 Manihot esculenta

608 Mastigamoeba balamuthi

609 Maylandia zebra

610 Medicago truncatula

611 Megachile rotundata

612 Megaselia scalaris

613 Melampsora laricis-populina

614 Melanconium sp. NRRL 54901

615 Melanomma pulvis-pyrius

616 Meleagris gallopavo

617 Meliniomyces bicolor E

618 Meliniomyces variabilis F

619 Melitaea cinxia

620 Melopsittacus undulatus

621 Merops nubicus
622 Mesitornis unicolor
623 Mesocricetus auratus
624 Metallosphaera sedula DSM 5348
625 Metarhizium acridum CQMa 102
626 Metarhizium robertsii
627 Metaseiulus occidentalis
628 Methanobacterium formicicum DSM 3637
629 Methanobrevibacter ruminantium M1
630 Methanobrevibacter sp. AbM4
631 Methanocaldococcus fervens AG86
632 Methanocella conradii HZ254
633 Methanococcoides burtonii DSM 6242
634 Methanococcus maripaludis S2
635 Methanocorpusculum labreanum Z
636 Methanoculleus bourgensis MS2
637 Methanofollis liminatans DSM 4140
638 Methanohalobium evestigatum Z-7303
639 Methanohalophilus mahii DSM 5219
640 Methanolobus psychrophilus R15
641 Methanomethylovorans hollandica DSM 15978
642 Methanoplanus limicola DSM 2279
643 Methanopyrus kandleri AV19
644 Methanoregula formicicum SMSP
645 Methanosaeta thermophila PT
646 Methanosalsum zhilinae DSM 4017
647 Methanosarcina acetivorans C2A
648 Methanosphaera stadtmanae DSM 3091
649 Methanosphaerula palustris E1-9c
650 Methanospirillum hungatei JF-1
651 Methanothermobacter marburgensis str. Marburg

652 Methanothermococcus okinawensis IH1

653 Methanothermus fervidus DSM 2088

654 Methanotorris igneus Kol 5

655 Metschnikowia bicuspidata var. bicuspidata NRRL YB-4993

656 Meyerozyma guilliermondii

657 Microbotryum violaceum

658 Microcebus murinus

659 Micromonas pusilla CCMP1545

660 Micromonas pusilla RCC299

661 Microplitis demolitor

662 Microsporum canis

663 Microsporum canis CBS 113480

664 Microsporum gypseum

665 Microtus ochrogaster

666 Millerozyma farinosa

667 Mimulus guttatus

668 Mixia osmundae IAM 14324

669 Mnemiopsis leidyi

670 Monacrosporium haptotylum CBS 200.50

671 Monascus purpureus

672 Monascus ruber NRRL 1597

673 Moniliophthora perniciosa FA553

674 Monocercomonoides sp. PA

675 Monodelphis domestica

676 Monosiga brevicollis

677 Moorella thermoacetica ATCC 39073

678 Morchella conica CCBAS932

679 Mortierella elongata

680 Mortierella verticillata NRRL 6337

681 Mucor circinelloides f. lusitanicus CBS 277.49

682 Mus musculus

683 Musa acuminata

684 Musca domestica

685 Mustela putorius furo

686 Myceliophthora fergusii

687 Myceliophthora sepedonium

688 Myceliophthora thermophila

689 Mycobacterium bovis AF2122_97

690 Mycobacterium leprae TN

691 Mycoplasma mycoides subsp. mycoides SC str. PG1

692 Mycosphaerella fijiensis

693 Mycosphaerella graminicola

694 Myotis brandtii

695 Myotis davidii

696 Myotis lucifugus

697 Myriangium duriaei CBS 260.36

698 Myriococcum thermophilum

699 Nadsonia fulvescens var. elongata DSM 6958

700 Naegleria fowleri

701 Naegleria gruberi

702 Naiadella fluitans ATCC 64713

703 Nannochloropsis gaditana

704 Nannospalax galili

705 Nanoarchaeum equitans Kin4-M

706 Nanorana parkeri

707 Nasonia vitripennis

708 Natrialba magadii ATCC 43099

709 Natrinema pellirubrum DSM 15624

710 Natronobacterium gregoryi SP2

711 Natronococcus occultus SP4

712 Natronomonas pharaonis DSM 2160

713 Naumovozyma castellii

714 Naumovozyma dairenensis

715 Nectria haematococca

716 Nelumbo nucifera

717 Nematocida parisii ERTm1

718 Nematocida parisii ERTm3

719 Nematocida sp. 1 ERTm2

720 Nematocida sp. 1 ERTm6

721 Nematostella vectensis

722 Neofusicoccum parvum UCRNP2

723 Neolamprologus brichardi

724 Neolentinus lepideus

725 Neosartorya fischeri NRRL 181

726 Neospora caninum

727 Nestor notabilis

728 Neurospora crassa OR74A

729 Neurospora discreta FGSC 8579

730 Neurospora tetrasperma FGSC 2508

731 Neurospora tetrasperma FGSC 2509

732 Nicotiana benthamiana

733 Nicotiana sylvestris

734 Nicotiana tomentosiformis

735 Nipponia nippon

736 Nitrosopumilus maritimus SCM1

737 Nomascus leucogenys

738 Nosema apis BRL 01

739 Nosema bombycis CQ1

740 Nosema ceranae BRL01

741 Notothenia coriiceps

742 Obba rivulosa

743 Ochotona princeps

744 Octodon degus

745 Oculimacula yallundae

746 Odobenus rosmarus

747 Ogataea angusta NCYC 495 leu1.1

748 Oidiodendron maius Zn

749 Oikopleura dioica

750 Omphalotus olearius

751 Onchocerca volvulus

752 Ophiobolus disseminans

753 Ophiostoma piceae UAMH 11346

754 Opisthocomus hoazin

755 Orcinus orca

756 Oreochromis niloticus

757 Ornithorhynchus anatinus

758 Orpinomyces sp. C1A

759 Orycteropus afer afer

760 Oryctolagus cuniculus

761 Oryza barthii

762 Oryza brachyantha

763 Oryza glaberrima

764 Oryza glumaepatula

765 Oryza meridionalis

766 Oryza nivara

767 Oryza punctata

768 Oryza rufipogon

769 Oryza sativa

770 Oryza sativa Indica Group

771 Oryzias latipes

772 Ostreococcus lucimarinus

773 Ostreococcus sp. RCC809

774 Ostreococcus tauri

775 Otolemur garnettii

776 Ovis aries

777 Oxytricha trifallax

778 Pachysolen tannophilus NRRL Y-2460

779 Pan paniscus

780 Pan troglodytes

781 Panicum hallii

782 Panicum virgatum

783 Panthera tigris altaica

784 Pantholops hodgsonii

785 Papio anubis

786 Paracoccidioides brasiliensis Pb01

787 Paracoccidioides brasiliensis Pb03

788 Paracoccidioides brasiliensis Pb18

789 Paramecium biaurelia

790 Paramecium caudatum

791 Paramecium multimicronucleatum

792 Paramecium primaurelia

793 Paramecium sexaurelia

794 Paramecium tetraurelia

795 Pararge aegeria

796 Parastagonospora nodorum SN15

797 Patellaria atrata

798 Patiria miniata

799 Paxillus involutus ATCC 200175

800 Paxillus rubicundulus Ve08.2h10

801 Pediculus humanus

802 Pelecanus crispus

803 Pelodiscus sinensis

804 Penicillium bilaiae ATCC 20851

805 Penicillium brevicompactum

806 Penicillium brevicompactum AgRF18

807 Penicillium canescens ATCC 10419

808 Penicillium chrysogenum

809 Penicillium chrysogenum Wisconsin 54-1255

810 Penicillium digitatum PHI26

811 Penicillium expansum ATCC 24692

812 Penicillium fellutanum ATCC 48694

813 Penicillium glabrum DAOM 239074

814 Penicillium janthinellum ATCC 10455

815 Penicillium lanosocoeruleum ATCC 48919

816 Penicillium oxalicum 114-2

817 Penicillium raistrickii ATCC 10490

818 Perkinsus marinus

819 Peromyscus maniculatus bairdii

820 Petromyzon marinus

821 Phaeoacremonium aleophilum UCRPA7

822 Phaeodactylum tricornutum

823 Phaeodactylum tricornutum unmapped

824 Phaeosphaeria nodorum

825 Phaethon lepturus

826 Phalacrocorax carbo

827 Phanerochaete carnosa HHB-10118-Sp

828 Phanerochaete chrysosporium

829 Phanerochaete chrysosporium RP-78

830 Phaseolus vulgaris

831 Phialophora europaea CBS 101466

832 Phlebia brevispora HHB-7030 SS6

833 Phlebiopsis gigantea

834 Phoenicopterus ruber

835 Phoenix dactylifera

836 Phycomyces blakesleeanus

837 Phyllostachys edulis

838 Physcomitrella patens

839 Physeter catodon

840 Phytophthora capsici

841 Phytophthora cinnamomi var. Cinnamomi

842 Phytophthora infestans T30-4

843 Phytophthora kernoviae

844 Phytophthora lateralis MPF4

845 Phytophthora parasitica

846 Phytophthora parasitica P1569

847 Phytophthora ramorum

848 Phytophthora sojae

849 Picea abies

850 Picea sitchensis

851 Pichia membranifaciens

852 Pichia pastoris

853 Pichia sorbitophila

854 Pichia stipitis

855 Picoides pubescens

856 Picrophilus torridus DSM 9790

857 Piedraia hortae

858 Piloderma croceum F 1598

859 Pinctada fucata

860 Pinus taeda

861 Piriformospora indica DSM 11827

862 Piromyces sp. E2

863 Pisolithus microcarpus 441

864 Pisolithus tinctorius Marx 270

865 Plasmodium berghei

866 Plasmodium chabaudi

867 Plasmodium cynomolgi

868 Plasmodium falciparum

869 Plasmodium inui
870 Plasmodium knowlesi
871 Plasmodium reichenowi
872 Plasmodium vinckei petteri
873 Plasmodium vinckei vinckei
874 Plasmodium vivax
875 Plasmodium yoelii
876 Plenodomus tracheiphilus IPT5
877 Pleomassaria siparia
878 Pleurotus ostreatus PC15
879 Pleurotus ostreatus PC9
880 Plicaturopsis crispa
881 Plutella xylostella
882 Pneumocystis jirovecii
883 Pneumocystis murina B123
884 Podiceps cristatus
885 Podospora anserina
886 Podospora anserina S mat+
887 Poecilia formosa
888 Poecilia reticulata
889 Polychaeton citri
890 Polyporus arcularius
891 Polysphondylium pallidum
892 Pongo abelii
893 Populus euphratica
894 Populus trichocarpa
895 Porphyridium purpureum
896 Postia placenta MAD 698-R
897 Postia placenta MAD-698-R-SB12
898 Primula veris
899 Pristionchus pacificus

900 Procavia capensis

901 Prunus mume

902 Prunus persica

903 Pseudo-nitzschia multiseries

904 Pseudomonas aeruginosa PAO1

905 Pseudopoces humilis

906 Pseudozyma antarctica T-34

907 Pseudozyma brasiliensis

908 Pseudozyma flocculosa

909 Pseudozyma hubeiensis SY62

910 Pterocles gutturalis

911 Pteropus alecto

912 Pteropus vampyrus

913 Puccinia graminis

914 Puccinia graminis f. sp. tritici 04KEN156_4

915 Puccinia striiformis

916 Puccinia striiformis f. sp. tritici PST-130

917 Puccinia triticina

918 Punctularia strigosozonata

919 Pundamilia nyererei

920 Pycnoporus coccineus

921 Pycnoporus coccineus BRFM310

922 Pygoscelis adeliae

923 Pyrenophora teres f. teres

924 Pyrenophora tritici-repentis

925 Pyrobaculum arsenaticum DSM 13514

926 Pyrococcus yayanosii CH1

927 Pyrolobus fumarii 1A

928 Pyronema confluens

929 Pyronema confluens CBS100304

930 Pyropia yezoensis

931 Pyrus x bretschneideri

932 Pythium aphanidermatum DAOM BR444

933 Pythium arrhenomanes ATCC 12531

934 Pythium irregulare DAOM BR486

935 Pythium iwayamai DAOM BR242034

936 Pythium ultimum

937 Pythium ultimum var. sporangiiferum BR650

938 Pythium vexans DAOM BR484

939 Python bivittatus

940 Ramaria acris UT-36052-T

941 Rasamsonia byssochlamydoides

942 Rattus norvegicus

943 Reticulomyxa filosa

944 Rhinopithecus roxellana

945 Rhipicephalus pulchellus

946 Rhizoctonia solani AG-1 IB

947 Rhizomucor miehei

948 Rhizomucor pusillus

949 Rhizophagus irregularis DAOM 181602

950 Rhizopogon vinicolor AM-OR11-026

951 Rhizopus delemar RA 99-880

952 Rhizopus microsporus var. chinensis CCTCC M201021

953 Rhizopus microsporus var. microsporus

954 Rhodnius prolixus

955 Rhodobacter sphaeroides 2.4.1

956 Rhodopirellula baltica SH 1

957 Rhodospirillum rubrum ATCC 11170

958 Rhodosporidium toruloides MTCC 457

959 Rhodotorula graminis WP1

960 Rhodotorula minuta MCA 4210

961 Rhytidhysteron rufulum

962 Ricinus communis

963 Rickenella mellea

964 Rickettsia prowazekii str. Madrid E

965 Rozella allomycis CSF55

966 Saccharata proteae CBS 121410

967 Saccharomyces arboricola

968 Saccharomyces bayanus

969 Saccharomyces bayanus var. uvarum

970 Saccharomyces castelli

971 Saccharomyces castellii

972 Saccharomyces cerevisiae M3707

973 Saccharomyces cerevisiae M3836

974 Saccharomyces cerevisiae M3837

975 Saccharomyces cerevisiae M3838

976 Saccharomyces cerevisiae M3839

977 Saccharomyces cerevisiae S288C

978 Saccharomyces cerevisiae YB210

979 Saccharomyces kudriavzevii

980 Saccharomyces kudriavzevii ZP591

981 Saccharomyces mikatae

982 Saccharomyces paradoxus

983 Saccoglossus kowalevskii

984 Saimiri boliviensis

985 Saitoella complicata NRRL Y-17804

986 Salinarchaeum sp. Harcht-Bsk1

987 Salinibacter ruber DSM 13855

988 Salix purpurea

989 Salmonella enterica subsp. enterica serovar Typhi str. CT18

990 Salpingoeca sp. ATCC 50818

991 Saprolegnia declina VS20

992 Saprolegnia parasitica cbs 223.65

993 Sarcophilus harrisii
994 Schistosoma haematobium
995 Schistosoma japonicum
996 Schistosoma mansoni
997 Schizochytrium aggregatum ATCC 28209
998 Schizochytrium limacinum ATCC MYA-1381
999 Schizophyllum commune H4-8
1000 Schizophyllum commune Loenen D
1001 Schizophyllum commune Tattone D
1002 Schizopora paradoxa
1003 Schizosaccharomyces cryophilus
1004 Schizosaccharomyces japonicus
1005 Schizosaccharomyces octosporus
1006 Schizosaccharomyces pombe
1007 Scleroderma citrinum Foug A
1008 Sclerotinia sclerotiorum
1009 Scytalidium thermophilum
1010 Sebacina vermifera MAFF 305830
1011 Selaginella moellendorffii
1012 Septoria musiva SO2202
1013 Septoria populicola
1014 Serinus canaria
1015 Serpula lacrymans var shastensis SHA21-2
1016 Serpula lacrymans var. lacrymans S7.3
1017 Serpula lacrymans var. lacrymans S7.9
1018 Sesamum indicum
1019 Setaria italica
1020 Setosphaeria turcica Et28A
1021 Shewanella oneidensis MR-1
1022 Shigella dysenteriae Sd197
1023 Shigella flexneri 2a str. 301

1024 Sistotremastrum niveocremeum HHB9708
1025 Sistotremastrum suecicum
1026 Sodiomyces alkalinus
1027 Solanum lycopersicum
1028 Solanum pimpinellifolium
1029 Solanum tuberosum
1030 Solenopsis invicta
1031 Sordaria macrospora
1032 Sorex araneus
1033 Sorghum bicolor
1034 Spathaspora passalidarum NRRL Y-27907
1035 Sphaerobolus stellatus
1036 Sphaeroforma arctica jp610
1037 Spirodela polyrhiza
1038 Spizellomyces punctatus daom br117
1039 Sporisorium reilianum SRZ2
1040 Sporobolomyces linderae CBS 7893
1041 Sporobolomyces roseus
1042 Sporormia fimetaria
1043 Sporothrix schenckii
1044 Spraguea lophii
1045 Staphylothermus hellenicus DSM 12710
1046 Stegastes partitus
1047 Stereum hirsutum FP-91666 SS1
1048 Streptococcus pneumoniae R6
1049 Streptococcus suis BM407
1050 Strigamia maritima
1051 Strigomonas culicis
1052 Strongylocentrotus purpuratus
1053 Strongyloides ratti
1054 Struthio camelus

1055 Stylonychia lemnae

1056 Suillus brevipes

1057 Suillus luteus UH-Slu-Lm8-n1

1058 Sulfolobus islandicus M.14.25

1059 Sus scrofa

1060 Symbiodinium minutum

1061 Symbiotaphrina kochii

1062 Taenia solium

1063 Taeniopygia guttata

1064 Takifugu rubripes

1065 Talaromyces aculeatus ATCC 10409

1066 Talaromyces marneffei ATCC 18224

1067 Talaromyces stipitatus ATCC 10500

1068 Taphrina deformans

1069 Tarenaya hassleriana

1070 Tarsius syrichta

1071 Tauraco erythrolophus

1072 Terfezia boudieri S1

1073 Tetrahymena borealis

1074 Tetrahymena elliotti

1075 Tetrahymena malaccensis

1076 Tetrahymena thermophila macronucleus

1077 Tetrahymena thermophila micronucleus

1078 Tetranychus urticae

1079 Tetraodon nigroviridis

1080 Tetrapisispora blattae

1081 Tetrapisispora phaffii

1082 Thalassiosira oceanica

1083 Thalassiosira pseudonana

1084 Thecamonas trahens ATCC 50062

1085 Theileria annulata

1086 Theileria parva

1087 Theobroma cacao

1088 Thermanaerovibrio acidaminovorans DSM 6589

1089 Thermoascus aurantiacus

1090 Thermoascus crustaceus

1091 Thermococcus barophilus MP

1092 Thermodesulfovibrio yellowstonii DSM 11347

1093 Thermofilum pendens Hrk 5

1094 Thermogladius cellulolyticus 1633

1095 Thermomyces dupontii

1096 Thermomyces lanuginosus

1097 Thermoplasma acidophilum DSM 1728

1098 Thermoplasmatales archaeon BRNA1

1099 Thermoproteus uzoniensis 768-20

1100 Thermosphaera aggregans DSM 11486

1101 Thermosynechococcus elongatus BP-1

1102 Thermotoga maritima MSB8

1103 Thielavia antarctica CBS 123565

1104 Thielavia appendiculata CBS 731.68

1105 Thielavia arenaria CBS 508.74

1106 Thielavia australiensis

1107 Thielavia hyrcaniae CBS 757.83

1108 Thielavia terrestris

1109 Thraustotheca clavata

1110 Tilletiaria anomala UBC 951

1111 Tinamus guttatus

1112 Torulaspora delbrueckii

1113 Toxocara canis

1114 Toxoplasma gondii

1115 Trachipleistophora hominis

1116 Trametes cinnabarina BRFM137

1117 Trametes sanguinea

1118 Trametes versicolor

1119 Trematosphaeria pertusa CBS 122368

1120 Tremella mesenterica

1121 Tribolium castaneum

1122 Trichaptum abietinum

1123 Trichechus manatus latirostris

1124 Trichinella spiralis

1125 Trichoderma asperellum CBS 433.97

1126 Trichoderma atroviride

1127 Trichoderma citrinoviride

1128 Trichoderma harzianum CBS 226.95

1129 Trichoderma longibrachiatum ATCC 18648

1130 Trichoderma reesei

1131 Trichoderma reesei RUT C-30

1132 Trichoderma virens

1133 Trichoderma virens Gv29-8

1134 Tricholoma matsutake 945

1135 Trichomonas vaginalis G3

1136 Trichophyton equinum

1137 Trichophyton interdigitale H6

1138 Trichophyton interdigitale MR816

1139 Trichophyton rubrum CBS 118892

1140 Trichophyton rubrum D6

1141 Trichophyton rubrum MR1448

1142 Trichophyton rubrum MR1459

1143 Trichophyton rubrum MR850

1144 Trichophyton tonsurans

1145 Trichophyton verrucosum HKI 0517

1146 Trichoplax adhaerens

1147 Trichosporon asahii var. asahii CBS 2479

1148 Trichosporon asahii var. asahii CBS 8904

1149 Trichosporon oleaginosus

1150 Trichuris muris

1151 Triticum aestivum

1152 Triticum urartu

1153 Tritirachium sp. CBS 265.96

1154 Trypanosoma brucei

1155 Trypanosoma congolense

1156 Trypanosoma cruzi

1157 Trypanosoma vivax

1158 Trypethelium eluteriae

1159 Tuber melanosporum

1160 Tulasnella calospora AL13_4D

1161 Tupaia belangeri

1162 Tupaia chinensis

1163 Tursiops truncatus

1164 Tyto alba

1165 Umbelopsis ramanniana AG

1166 Uncinocarpus reesii 1704

1167 Ursus maritimus

1168 Ustilago hordei

1169 Ustilago maydis

1170 Vaccinium corymbosum

1171 Vanderwaltozyma polyspora

1172 Vavraia culicis floridensis

1173 Verticillium alfalfae VaMs.102

1174 Verticillium dahliae

1175 Vibrio cholerae O1 biovar El Tor str. N16961

1176 Vibrio fischeri ES114

1177 Vicugna pacos

1178 Vitis vinifera

1179 Vitrella brassicaformis CCMP3155

1180 Vittaforma corneae ATCC 50505

1181 Volvariella volvacea V23

1182 Volvox carteri

1183 Vulcanisaeta distributa DSM 14429

1184 Wallemia ichthyophaga EXF-994

1185 Wallemia sebi

1186 Wickerhamomyces anomalus NRRL Y-366-8

1187 Wilcoxina mikolae CBS 423.85

1188 Wolfiporia cocos MD-104 SS10

1189 Wuchereria bancrofti

1190 Xanthomonas campestris pv. campestris str. ATCC 33913

1191 Xanthoria parietina 46-1-SA22

1192 Xenopus laevis

1193 Xenopus tropicalis

1194 Xiphophorus maculatus

1195 Xylona heveae TC161

1196 Yarrowia lipolytica CLIB122

1197 Yersinia enterocolitica subsp. enterocolitica 8081

1198 Yersinia pestis CO92

1199 Zasmidium cellare ATCC 36951

1200 Zea mays

1201 Zonotrichia albicollis

1202 Zopfia rhizophila

1203 Zygosaccharomyces bailii CLIB 213

1204 Zygosaccharomyces bailii ISA1307

1205 Zygosaccharomyces rouxii

1206 Zymoseptoria tritici
