# Supplementary material for: Comparative genomic analysis of the ‘pseudofungus’ Hyphochytrium catenoides
Source: Open Biol. 2018 Jan 10;8(1):170184. doi: 10.1098/rsob.170184 (PMC5795050; doi:10.1098/rsob.170184)
Supplement: Figure S3 [file rsob170184supp4.pdf]

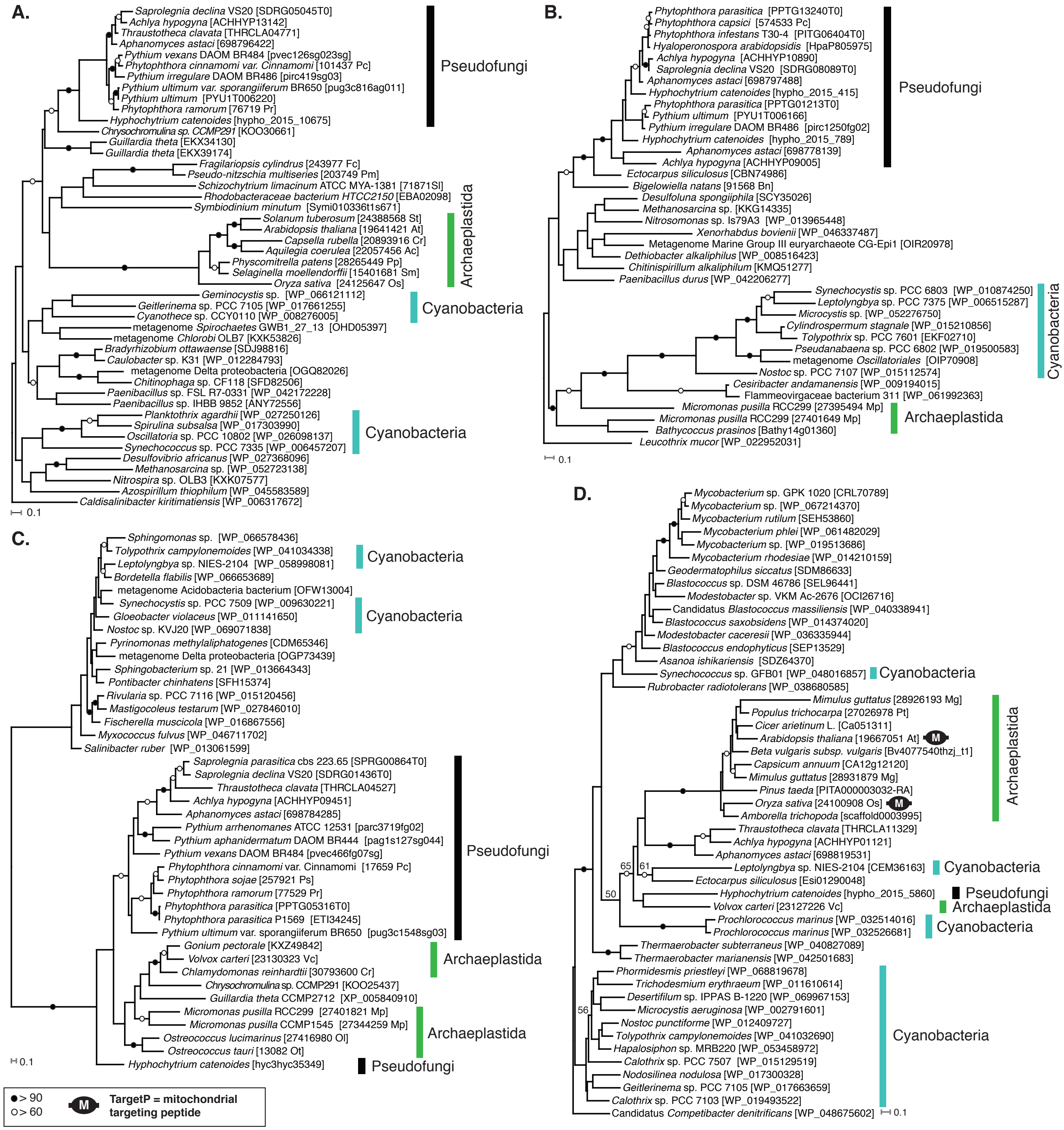
A.
Saprolegnia declina VS20 [SDRG05045T0]
Achlya hypogyna [ACHHYP13142]
Thraustotheca clavata [THRCLA04771]
Aphanomyces astaci [698796422]
Pythium vexans DAOM BR484 [pvec126sg023sg]
Phytophthora cinnamomi var. Cinnamomi [101437 Pc]
Pythium irregulare DAOM BR486 [pirc419sg03]
Pythium ultimum var. sporangiiferum BR650 [pug3c816ag011]
Pythium ultimum [PYU1T006220]
Phytophthora ramorum [76719 Pr]
Hyphochytrium catenoides [hypho_2015_10675]
Chrysochromulina sp. CCMP291 [KOO30661]
Guillardia theta [EKX34130]
Guillardia theta [EKX39174]
Pseudofungi
Fragilariopsis cylindrus [243977 Fc]
Pseudo-nitzschia multiseries [203749 Pm]
Schizochytrium limacinum ATCC MYA-1381 [71871Sl]
Rhodobacteraceae bacterium HTCC2150 [EBA02098]
Symbiodinium minutum [Symi010336t1s671]
Solanum tuberosum [24388568 St]
Arabidopsis thaliana [19641421 At]
Capsella rubella [20893916 Cr]
Aquilegia coerulea [22057456 Ac]
Physcomitrella patens [28265449 Pp]
Selaginella moellendorffii [15401681 Sm]
Oryza sativa [24125647 Os]
Archaeplastida
Geminocystis sp. [WP_066121112]
Geitlerinema sp. PCC 7105 [WP_017661255]
Cyanothece sp. CCY0110 [WP_008276005]
metagenome Spirochaetes GWB1_27_13 [OHD05397]
metagenome Chlorobi OLB7 [KXK53826]
Cyanobacteria
Bradyrhizobium ottawaense [SDJ98816]
Caulobacter sp. K31 [WP_012284793]
metagenome Delta proteobacteria [OGQ82026]
Chitinophaga sp. CF118 [SFD82506]
Paenibacillus sp. FSL R7-0331 [WP_042172228]
Paenibacillus sp. IHBB 9852 [ANY72556]
Planktothrix agardhii [WP_027250126]
Spirulina subsalsa [WP_017303990]
Oscillatoria sp. PCC 10802 [WP_026098137]
Synechococcus sp. PCC 7335 [WP_006457207]
Cyanobacteria
Desulfovibrio africanus [WP_027368096]
Methanosarcina sp. [WP_052723138]
Nitrospira sp. OLB3 [KXK07577]
Azospirillum thiophilum [WP_045583589]
Caldisalinibacter kiritimatiensis [WP_006317672]
0.1
B.
Phytophthora parasitica [PPTG13240T0]
Phytophthora capsici [574533 Pc]
Phytophthora infestans T30-4 [PITG06404T0]
Hyaloperonospora arabidopsidis [HpaP805975]
Achlya hypogyna [ACHHYP10890]
Saprolegnia declina VS20 [SDRG08089T0]
Aphanomyces astaci [698797488]
Hyphochytrium catenoides [hypho_2015_415]
Phytophthora parasitica [PPTG01213T0]
Pythium ultimum [PYU1T006166]
Pythium irregulare DAOM BR486 [pirc1250fg02]
Hyphochytrium catenoides [hypho_2015_789]
Aphanomyces astaci [698778139]
Achlya hypogyna [ACHHYP09005]
Ectocarpus siliculosus [CBN74986]
Pseudofungi
Bigelowiella natans [91568 Bn]
Desulfoluna spongiiphila [SCY35026]
Methanosarcina sp. [KKG14335]
Nitrosomonas sp. Is79A3 [WP_013965448]
Xenorhabdus bovienii [WP_046337487]
Metagenome Marine Group III euryarchaeote CG-Epi1 [OIR20978]
Dethiobacter alkaliphilus [WP_008516423]
Chitinispirillum alkaliphilum [KMQ51277]
Paenibacillus durus [WP_042206277]
Synechocystis sp. PCC 6803 [WP_010874250]
Leptolyngbya sp. PCC 7375 [WP_006515287]
Microcystis sp. [WP_052276750]
Cylindrospermum stagnale [WP_015210856]
Tolypothrix sp. PCC 7601 [EKF02710]
Pseudanabaena sp. PCC 6802 [WP_019500583]
metagenome Oscillatoriales [OIP70908]
Nostoc sp. PCC 7107 [WP_015112574]
Cesiribacter andamanensis [WP_009194015]
Flammeovirgaceae bacterium 311 [WP_061992363]
Cyanobacteria
Micromonas pusilla RCC299 [27395494 Mp]
Micromonas pusilla RCC299 [27401649 Mp]
Bathycoccus prasinos [Bathy14g01360]
Archaeplastida
Leucothrix mucor [WP_022952031]
0.1
C.
Sphingomonas sp. [WP_066578436]
Tolypothrix campylonemoides [WP_041034338]
Leptolyngbya sp. NIES-2104 [WP_058998081]
Bordetella flabilis [WP_066653689]
Cyanobacteria
metagenome Acidobacteria bacterium [OFW13004]
Synechocystis sp. PCC 7509 [WP_009630221]
Gloeobacter violaceus [WP_011141650]
Nostoc sp. KVJ20 [WP_069071838]
Cyanobacteria
Pyrinomonas methylaliphatogenes [CDM65346]
metagenome Delta proteobacteria [OGP73439]
Sphingobacterium sp. 21 [WP_013664343]
Pontibacter chinhatens [SFH15374]
Rivularia sp. PCC 7116 [WP_015120456]
Mastigocoleus testarum [WP_027846010]
Fischerella muscicola [WP_016867556]
Myxococcus fulvus [WP_046711702]
Salinibacter ruber [WP_013061599]
Saprolegnia parasitica cbs 223.65 [SPRG00864T0]
Saprolegnia declina VS20 [SDRG01436T0]
Thraustotheca clavata [THRCLA04527]
Achlya hypogyna [ACHHYP09451]
Aphanomyces astaci [698784285]
Pythium arrhenomanes ATCC 12531 [parc3719fg02]
Pythium aphanidermatum DAOM BR444 [pag1s127sg044]
Pythium vexans DAOM BR484 [pvec466fg07sg]
Phytophthora cinnamomi var. Cinnamomi [17659 Pc]
Phytophthora sojae [257921 Ps]
Phytophthora ramorum [77529 Pr]
Phytophthora parasitica [PPTG05316T0]
Phytophthora parasitica P1569 [ETI34245]
Pythium ultimum var. sporangiiferum BR650 [pug3c1548sg03]
Pseudofungi
Gonium pectorale [KXZ49842]
Volvox carteri [23130323 Vc]
Chlamydomonas reinhardtii [30793600 Cr]
Archaeplastida
Chrysochromulina sp. CCMP291 [KOO25437]
Guillardia theta CCMP2712 [XP_005840910]
Micromonas pusilla RCC299 [27401821 Mp]
Micromonas pusilla CCMP1545 [27344259 Mp]
Ostreococcus lucimarinus [27416980 Ol]
Ostreococcus tauri [13082 Ot]
Archaeplastida
Hyphochytrium catenoides [hyc3hyc35349]
Pseudofungi
0.1
D.
Mycobacterium sp. GPK 1020 [CRL70789]
Mycobacterium sp. [WP_067214370]
Mycobacterium rutilum [SEH53860]
Mycobacterium phlei [WP_061482029]
Mycobacterium sp. [WP_019513686]
Mycobacterium rhodesiae [WP_014210159]
Geodermatophilus siccatus [SDM86633]
Blastococcus sp. DSM 46786 [SEL96441]
Modestobacter sp. VKM Ac-2676 [OCI26716]
Candidatus Blastococcus massiliensis [WP_040338941]
Blastococcus saxobsidens [WP_014374020]
Modestobacter caceresii [WP_036335944]
Blastococcus endophyticus [SEP13529]
Asanoa ishikariensis [SDZ64370]
Synechococcus sp. GFB01 [WP_048016857]
Cyanobacteria
Rubrobacter radiotolerans [WP_038680585]
Mimulus guttatus [28926193 Mg]
Populus trichocarpa [27026978 Pt]
Cicer arietinum L. [Ca051311]
Arabidopsis thaliana [19667051 At]
M
Beta vulgaris subsp. vulgaris [Bv4077540thzj_t1]
Capsicum annuum [CA12g12120]
Mimulus guttatus [28931879 Mg]
Pinus taeda [PITA000003032-RA]
Oryza sativa [24100908 Os]
M
Amborella trichopoda [scaffold0003995]
Archaeplastida
Thraustotheca clavata [THRCLA11329]
Achlya hypogyna [ACHHYP01121]
Aphanomyces astaci [698819531]
65
61
Leptolyngbya sp. NIES-2104 [CEM36163]
Cyanobacteria
Ectocarpus siliculosus [Esi01290048]
50
Hyphochytrium catenoides [hypho_2015_5860]
Pseudofungi
Volvox carteri [23127226 Vc]
Archaeplastida
Prochlorococcus marinus [WP_032514016]
Prochlorococcus marinus [WP_032526681]
Cyanobacteria
Thermaerobacter subterraneus [WP_040827089]
Thermaerobacter marianensis [WP_042501683]
Phormidesmis priestleyi [WP_068819678]
Trichodesmium erythraeum [WP_011610614]
Desertifilum sp. IPPAS B-1220 [WP_069967153]
56
Microcystis aeruginosa [WP_002791601]
Nostoc punctiforme [WP_012409727]
Tolypothrix campylonemoides [WP_041032690]
Hapalosiphon sp. MRB220 [WP_053458972]
Calothrix sp. PCC 7507 [WP_015129519]
Nodosilinea nodulosa [WP_017300328]
Geitlerinema sp. PCC 7105 [WP_017663659]
Calothrix sp. PCC 7103 [WP_019493522]
Cyanobacteria
Candidatus Competibacter denitrificans [WP_048675602]
0.1
> 90
> 60
M
TargetP = mitochondrial targeting peptide
